# Supplementary material for: Dual Plasmepsin-Targeting Antimalarial Agents Disrupt Multiple Stages of the Malaria Parasite Life Cycle
Source: Cell Host Microbe. 2020 Apr 8;27(4):642–658.e12. doi: 10.1016/j.chom.2020.02.005 (PMC7146544; doi:10.1016/j.chom.2020.02.005)
Supplement: Document S1. Figures S1–S7 and Supplemental References [file mmc1.pdf]

Cell Host & Microbe, Volume *27*

Supplemental Information

Dual Plasmepsin-Targeting Antimalarial Agents

Disrupt Multiple Stages

# of the Malaria Parasite Life Cycle

Paola Favuzza, Manuel de Lera Ruiz, Jennifer K. Thompson, Tony Triglia, Anna Ngo, Ryan W.J. Steel, Marissa Vavrek, Janni Christensen, Julie Healer, Christopher Boyce, Zhuyan Guo, Mengwei Hu, Tanweer Khan, Nicholas Murgolo, Lianyun Zhao, Jocelyn Sietsma Penington, Kitsanapong Reaksudsan, Kate Jarman, Melanie H. Dietrich, Lachlan Richardson, Kai-Yuan Guo, Sash Lopaticki, Wai-Hong Tham, Matthias Rottmann, Tony Papenfuss, Jonathan A. Robbins, Justin A. Boddey, Brad E. Sleebs, Hélène Jousset Sabroux, John A. McCauley, David B. Olsen, and Alan F. Cowman

Supplemental Information

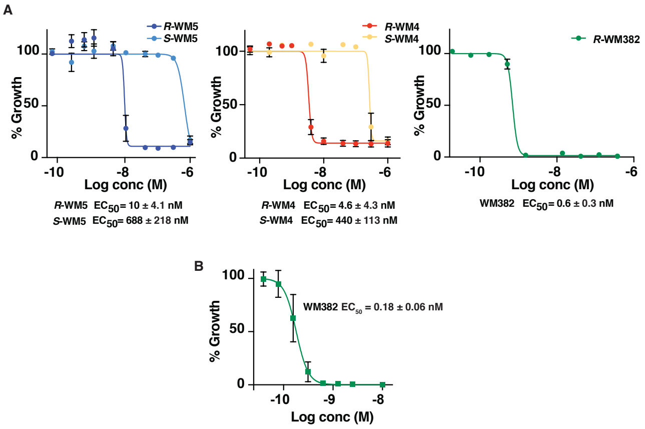

C

Parasite line	Start of amplicon	End of amplicon	Size of amplicon (kb)	Sequence end, start	Copy Number	EC_{50} (nM)	
						WM5	WM4
3D7	-	-	-	-	1	11.1±3.7*	14.2±6.5#
3D7-WM5.1	08: 409,250	08: 429,220	20	45xT, 31xT	8-10	106.8±2.2*	115.0±18.8#
3D7-WM5.2	08: 393,290	08: 426,970	34	51xT, 30xA	7-8	116.9±6.4*	141.7±5.5#
3D7-WM5.3	08: 409,250	08: 429,210	20	45xT, 31xT	8-10	117.9± 4.7*	135.3±3.8#
3D7-WM4.1	08: 398,500	08: 429,200	31	27xT, 31xT	6	51.82±19.7#	65.9±34.8#
3D7-WM4.2	08: 415,500	08: 429,200	14	20xT, 31xT	8	143.77± 46.6#	139.5± 52.7#

* ± SEM # ± SD

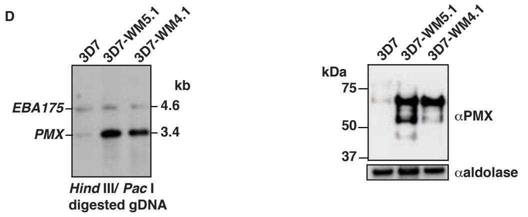

Supplementary Figure 1. Related to Figure 1. Growth inhibition of WM4, WM5 and WM382 enantiomers against *P. falciparum* and analysis of WM4 and WM5-resistant parasite lines. A. The 3D7 parasite line was used to derive growth inhibition plots for *R* and *S* enantiomers of WM5 (left), *R* and *S* enantiomers of WM4 (middle) and the *R* enantiomer of WM382 (right). Each experiment was performed in triplicate; mean with SEM are shown. **B.** *P. knowlesi* blood stage parasites were used to derive a growth inhibition plot for WM382. Dots represent the average of 3 repeats carried out in triplicate; error bars represent SD. **C.** The breakpoints in the genome of *P. falciparum* parasites selected for resistance to WM5 and WM4. The genomes of the WM5-resistant parasites 3D7-WM5.1, 3D7-WM5.2, 3D7-WM5.3 and WM4-resistant parasites 3D7-WM4.1 and 3D7-WM5.2 were sequenced and amplification events defined. The specific breakpoints for each were determined to define the start and end of the amplicon as well as the size of each amplification unit. Also determined were the EC_{50} of each parasite line for both WM4 and WM5. **D.** Left panel: southern blot of genomic DNA from 3D7-WM5.1 and 3D7-WM4.1 to confirm amplification of the *PMX* gene, compared to 3D7 wild type parasites. gDNA was digested with *Hind* III/*Pac* I and probed with both *PMX* and EBA175 genes. The expected 3.4 kb band for *PMX* and the expected 4.6 kb band for the EBA175 gene are shown. Right panel: immuno-blot of 3D7 WT, 3D7-WM5.1 and 3D7-WM4.1 schizont parasites with anti-PMX antibodies to show that this enzyme was overexpressed in the WM5- and WM4-resistant parasite lines, compared to 3D7 wild type parasites. An aldolase panel is shown at the bottom as a loading control. Aldolase was detected with anti-aldolase antibodies.

A

	EC_{50} (nM)				
**Parasite**	**Chloroquine**	**Atovaquone**	**Mefloquine**	**WM4**	**WM382**
3D7	14.5 ± 0.7	1.95 ± 0.1	10.4 ± 2.2	7 ± 0.5	0.48 ± 0.3
3D7-WM4.1	10.65 ± 3.3	0.97 ± 0.03	5.9 ± 1.13	58 ± 1.4	0.68 ± 0.1
CAM3R539T	114.5 ± 21.9	2.05 ± 0.06	9.15 ± 2.6	13 ± 1.4	0.25 ± 0.04
CAM3 REV	102.5 ± 38.8	1.05 ± 0.07	3.8 ± 0.0	12 ± 1.4	0.22 ± 0.01
Dd2	99.5 ± 14.8	1.85 ± 0.07	11.5 ± 2.1	8.55 ± 0.3	0.26 ± 0.01
Dd2-ATV	120 ± 28.2	28.5 ± 3.5	3.2 ± 0.1	5.85 ± 0.3	0.23 ± 0.02
K1	160 ± 14.1	2.4 ± 0.4	4.75 ± 2.7	13.5 ± 2.1	0.65 ± 0.4
K1Mef[2]	120 ± 14.1	5.45 ± 4.1	19.5 ± 13.4	11 ± 0	0.36 ± 0.09
W2Mef[3]	99.5 ± 28.9	2.95 ± 0.35	25 ± 2.8	9.4 ± 2.2	0.25 ± 0
PNG1776	69 ± 21.2	1.1 ± 0.14	1.95 ± 0.2	5.6 ± 1.4	0.21 ± 0

B

		EC_{50} (nM)	
	**RSA survival**	**WM5**	**WM4**
3D7	7.77%	12	13
3D7-WM4.1	9.96%	103	102
3D7-WM5.1	9.72%	36	69
Cam3 R539T	32.81%	90	29
Cam3 Rev	11.43%	73	33

C

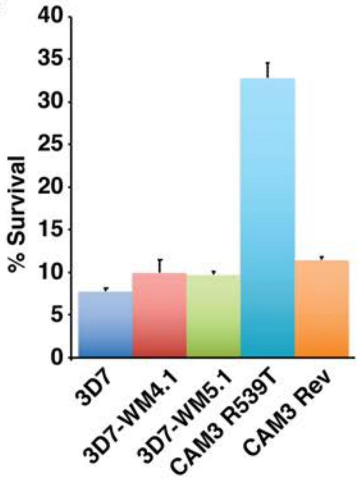

Supplementary Figure 2. Related to Figure 1. WM4 and WM382 do not show cross-resistance with *P. falciparum* lines resistant to chloroquine, atovaquone, mefloquine and artemisinin. A. EC_{50} determination for different *P. falciparum* lines shown (Parasite) to chloroquine, atovaquone, mefloquine, WM4 and WM382. Experiments have been done twice independently with three replicas per experiment. Shown is mean and standard deviation. **B** and **C.** Ring-stage survival assay to determine resistance to artemisinin. Shown are experiments done three independent times.

A

Parasite lines	Day 0 IC_{50} (nM)	Day 14 IC_{50} (nM)	Day 28 IC_{50} (nM)
3D7	10.96	13.95	11.65
3D7-WM5.1	202.8	228.5	177.5
3D7 + 3D7-WM5.1	87.9	120.6	17.33
3D7	5.9	5.2	5.4
3D7-WM4.1	78.8	89.9	79.4
3D7 + 3D7-WM4.1	34.1	42.1	7.3

B

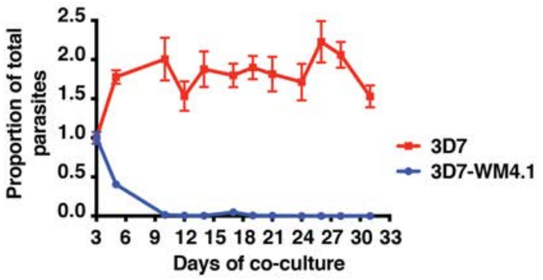

C

Experiment 1 (90 days)

Compound	Selection Pressure (nM)	Initial Inoculum	Outcome
Atovaquone	10	$1x10^9$<br>$1x10^8$<br>$1x10^7$<br>$1x10^6$<br>$1x10^5$	-<br>-<br>1 of 1 (day 56)<br>-<br>-
WM382	1.5	$1x10^9$<br>$1x10^8$<br>$1x10^7$<br>$1x10^6$<br>$1x10^5$	-<br>-<br>-<br>-<br>-

Experiment 2 (60 days)

Compound	Selection Pressure (nM)	Initial Inoculum	Outcome
Atovaquone	10	$1x10^8$<br>$1x10^7$<br>$1x10^6$	1 of 3 (day 41)<br>-<br>-
WM4	80	$1x10^8$<br>$1x10^7$<br>$1x10^6$	-<br>-<br>-
WM382	1.5	$1x10^8$<br>$1x10^7$<br>$1x10^6$	-<br>-<br>-

Supplementary Figure 3. Related to Figure 1. Time to resistance and fitness studies. A. 3D7-WM5.1 and 3D7-WM4.1 parasites are less fit then the 3D7 parental parasite line. 3D7 and 3D7-WM5.1 or 3D7-WM4.1 were mixed together in a 1:1 ratio and EC_{50} determined at day 0, 14 and 28 as a measure of the proportion of each parasite in the mixture over time. **B.** 3D7 and 3D7-WM4.1 were mixed together in a 1:1 ratio and the level of each parasite in the population monitored over 33 days by real time quantitative polymerase chain reaction. The proportion of each population was determined relative to the control RNA actin. **C.** Time to resistance studies comparing the selection of atovaquone resistance to that of WM4 and WM382. The initial inoculum of *P. falciparum* parasites was varied from $1x10^5$ to $1x10^9$ and these were selected on a concentration equivalent to the EC_{90} of WM4, WM382 and atovaquone and recrudescence of parasitemia followed over 96 and 60 days for each experiment respectively. Shown are two independent experiments.

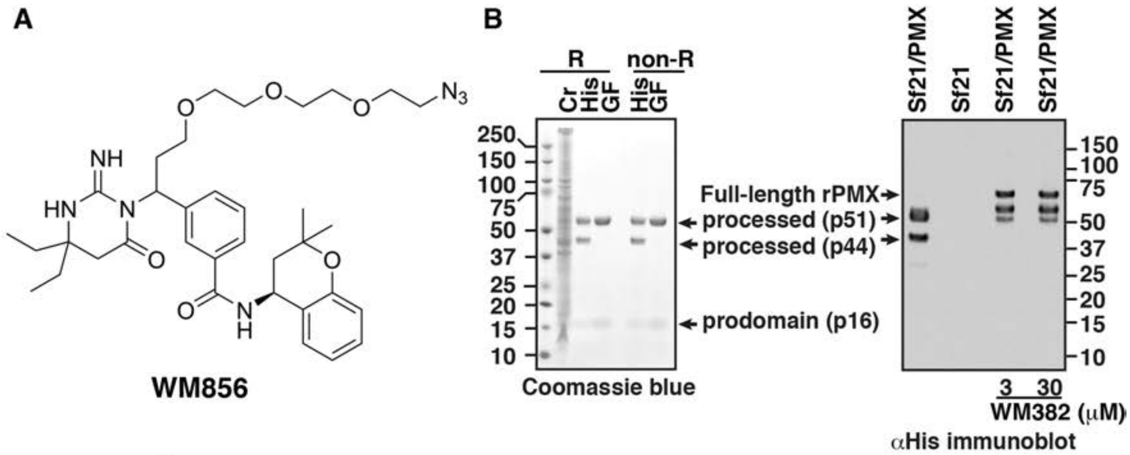

C

PROTEASE	PROTEIN	SEQUENCE									
PMX	SUB1	D	Y	K	S	M	L	E	V	E	N
PMX	AMA1	N	L	F	S	S	I	E	I	V	E
PMX	Rh2N	S	F	H	S	F	I	Q	E	G	K
PMX	Rh2C	H	T	A	S	L	V	Q	I	T	Q
PMX	Rh1	K	P	F	F	F	I	Q	L	N	T
PMX	Rh4	I	L	N	S	F	V	Q	I	N	S
PMX	Rh5	K	N	V	N	F	L	Q	Y	H	F
PMIX	Ripr	N	N	I	S	M	L	E	I	Q	N
PMX	EBA181	N	R	N	S	F	V	Q	R	S	Y
PMX	EBA175	R	I	L	S	F	L	D	S	R	I
PMX	EBA140	K	F	N	S	L	S	E	N	E	D
PMX	PMX	K	N	F	I	A	L	E	N	K	N
PMX	PMX	S	D	N	S	S	I	E	K	N	F
PMX	SUB2	S	K	R	S	N	I	E	K	E	I
PMIX	PMIX	D	N	T	N	T	S	Q	E	N	F
PMIX	RON3	K	E	I	S	F	L	E	R	R	E
PMIX	RAP1	S	S	E	S	F	L	E	N	K	S
PMIX	ASP	K	F	L	S	L	L	Q	L	N	L

Supplementary Figure 4. Related to Figure 2. Expression and purification of active recombinant *P. falciparum* PMX enzyme and analysis of cleavage site consensus sequence. A. Chemical structure of WM856. The compound was used for cross-linking to sepharose beads and affinity purification of PMX. **B.** Expression and purification of *P. falciparum* PMX from insect cells. PMX was purified from insect cell crude extract (Cr) by Histidine affinity purification (His) and size exclusion chromatography (GF). Three bands were identified by Coomassie stained gel electrophoresis. Mass spectrometry of each band identified them as the full-length enzyme with prodomain and catalytic domain (pro-cat) of approximately 60 kDa, the catalytic domain (cat) of 45 kDa and the prodomain (prodom) of 16 kDa. R, reduced; non-R, nonreduced. **C.** PMX and PMIX cleavage sites for SUB1 and RAP (Pino *et al.*, 2017) and potential cleavage sites of other proteins from *P. falciparum*. The conserved amino acids that make up the consensus sequence are shown in red.

A

Substrates	Parent	Processed by PMX	
SUB1	DABCYL-G-SMLEVENDAE-GE-EDANS<br>$[M+H]^+$ 1878 (Expected 1878)	DABCYL-G-SM<br>$[M+H]^+$ 545 (Expected 545)	LEVENDAE-GE-EDANS<br>$[M+H]^+$ 1352 (Expected 1352)
RAP1	DABCYL-G-FSSESFLENK-E-EDANS<br>$[M+H]^+$ 1873 (Expected 1873)	DABCYL-G-FSSESF<br>$[M+H]^+$ 1011 (Expected 1011)	LENK-E-EDANS<br>$[M+H]^+$ 879 (Expected 879)
Rh2N	DABCYL-HSFIQEGKE-E-EDANS<br>$[M+H]^+$ 1703 (Expected 1703)	DABCYL-HSF<br>$[M+H]^+$ 641 (Expected 641)	IQEGKE-E-EDANS<br>$[M+H]^+$ 1080 (Expected 1080)
RON3	DABCYL-KEISFLERR-E-EDANS<br>$[M+2H]^{2+}$ 903 (Expected 903)	DABCYL-KEISF<br>$[M+H]^+$ 874 (Expected 874)	LERR-E-EDANS<br>$[M+H]^+$ 950 (Expected 950)
EBA175	DABCYL-RILSFLDSRI-E-EDANS<br>$[M+2H]^{2+}$ 924 (Expected 924)	DABCYL-RILSF<br>$[M+H]^+$ 886 (Expected 886)	LDSRI-E-EDANS<br>$[M+H]^+$ 980 (Expected 980)
EBA181	DABCYL-NRNSFVQRSY-E-EDANS<br>$[M+2H]^{2+}$ 950 (Expected 950)	DABCYL-NRNSF<br>$[M+H]^+$ 888 (Expected 888)	VQRSY-E-EDANS<br>$[M+2H]^{2+}$ 514 (Expected 514)

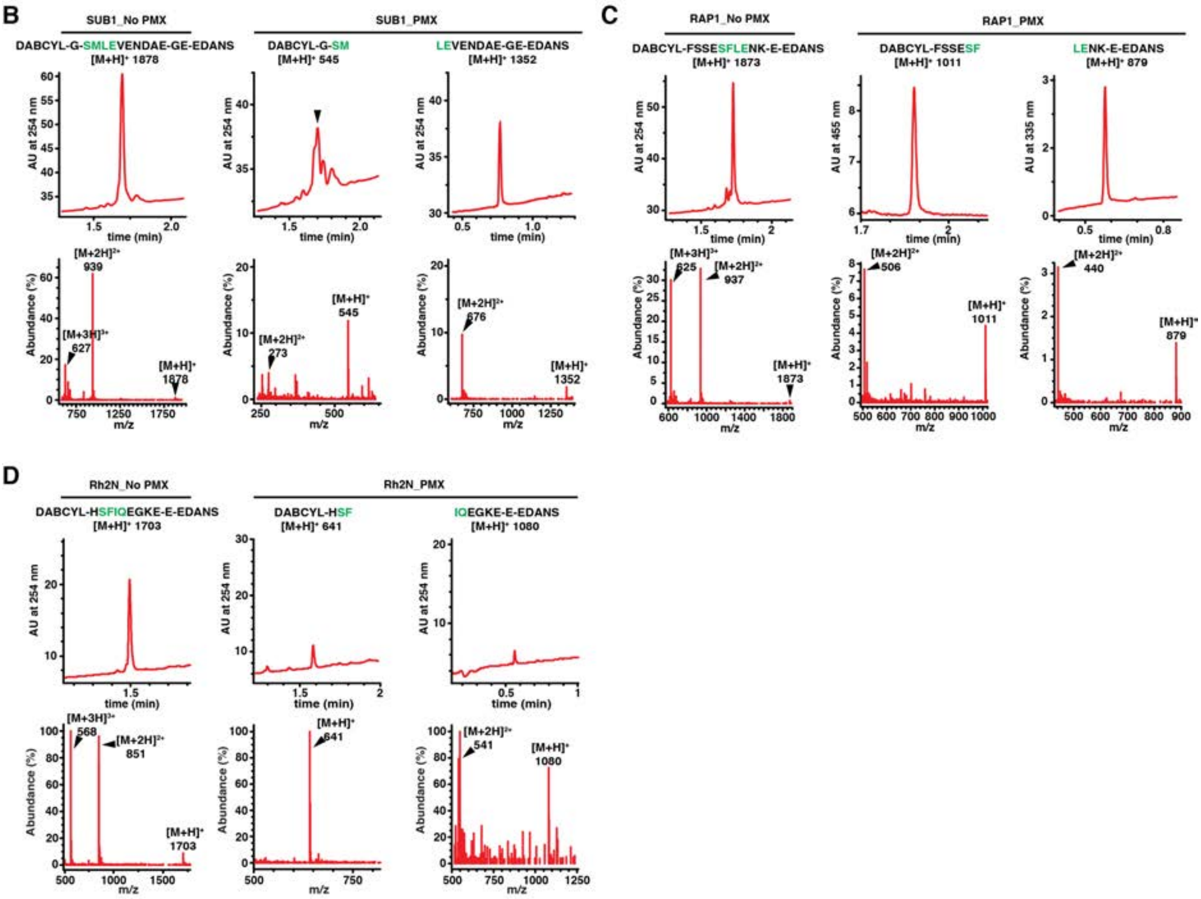

Supplementary Figure 5. Related to Figure 2. Mass spectrometry identification of *P. falciparum* peptide species before and after recombinant PMX cleavage. A. LC-ES/MS analysis of peptides and cleavage products after incubation with recombinant PMX (rPMX). Shown are the experimental and expected (in brackets) mass of each molecular species processed by rPMX. **B.** SUB1peptide without and with rPMX and analysed by LC-ES/MS to identify the molecular species present. **C.** RAP1 peptide incubated without and with rPMX and analysed by LC-ES/MS to identify the molecular species present. **D.** Rh2N peptide incubated without and with rPMX and analysed by LC-ES/MS to identify the molecular species present. For all substrates, SUB1, RAP1 and Rh2N, RON3, EBA175 and EBA181 fluorogenic peptides (50 µM) were incubated with and without rPMX (50 nM) and the products detected LC-ES/MS, indicating cleavage occurs on the C-terminal side of a hydrophobic amino acid (usually Phe).

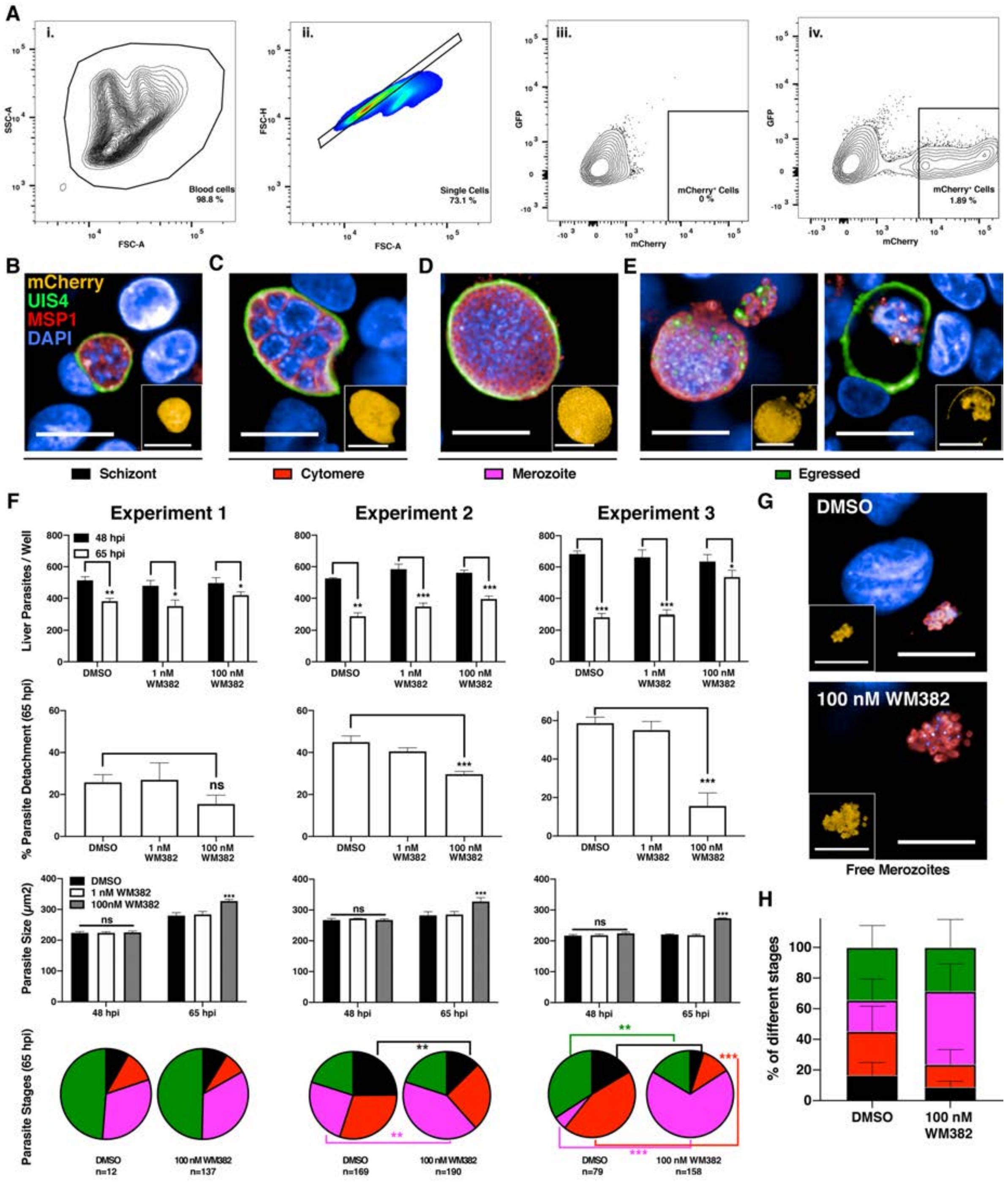

Supplementary Figure 6. Related to Figure 5. Flow cytometry analysis of *Pb*mCherryLuci infected whole blood and *in vitro* analysis of WM382 activity against *P. berghei* liver parasites (data supporting Fig. 5). A. Using a drop of tail blood, at least 1 x 10^6 events in the whole blood cells gate were collected (i) from which single cells were gated for analysis (ii). The mCherry$^+$ gate was set using an uninfected sample (iii) with a positive control shown (iv). **B-H.** Classification of *Pb*mCherryLuci liver parasites from infected HepG2 cultures was performed at 65 hpi and results from 3 independent experiments are shown. **B.** Example of a schizont stage without plasma membrane invaginations (MSP1, red) and intact UIS4$^+$ parasitophorous vacuole membrane (PVM, green) that was quantitated below (black) in the pie charts (F, bottom panels); inset shows mCherry fluorescence (orange, cytoplasmic).

C. Example of a cytomere stage with intact PVM (UIS4, green) and clear plasma membrane invaginations (MSP1, red) surrounding multiple nuclei (DAPI, blue) that was quantitated below (blueblue) in the pie charts (F, bottom panels). **D.** Example of a merozoite stage with extensive membrane invaginations (MSP1, red) surrounding single nuclei (DAPI, blue) with well-defined cytoplasm (mCherry, orange) and intact PVM (UIS4, green) that was quantitated below (magenta) in the pie charts (F, bottom panels) **E.** Example of egressed stages with evidence for PVM degradation (UIS4, left) or significant loss of parasite material within $UIS4^{+}$ vacuoles (right) that was quantitated below (yellowyellow) in the pie charts (F, bottom panels). **F.** Three independent experiments examining the effects of DMSO (control) and WM382 (1 nM and 100 nM) treatment from 24 hpi on *Pb*mCherryLuci liver parasite growth and egress using HepG2 cells *in vitro.* **1st row:** Treatment with 1 nM or 100 nM WM382 did not reduce the number of liver parasites at 48 hpi, indicating no parasite killing by WM382. The number of parasites per well decreased from 48 hpi to 65 hpi in all conditions, indicating that WM382 treated parasites are able to detach from the monolayer to be released into the supernatant. **2nd row:** Quantitation of parasite detachment and loss from the monolayer between 48 and 65 hpi suggests a decrease in detachment when treated with 100 nM WM382. **3rd row:** There was no difference in parasite size at 48 hpi consistent with there being no cidal or static effects of WM382 on liver parasite growth. There was however an increased size of parasites treated with 100 nM WM382 at 65 hpi consistent with an inhibitory effect on egress and detachment of mature *P. berghei*-infected hepatocytes. **4th row:** Quantification of schizont, cytomere, mature merozoite and egressed forms shows that developmentdevelopment to merozoite stages is normal in 100 nM WM382 treated cultures, and that at least some parasites treated with 100 nM WM382 successfully egress. Consistent with a decrease in cell detachment (second row) and increase in parasite size (third row), 100 nM WM382 had an increased proportion of merozoite stage parasites indicating an inhibition of parasite egress that precedes cell detachment. **G.** Free merozoites (DAPI, blue; MSP1, red; inset, mCherry cytoplasm) were seen in both control and 100 nM WM382 treated wells, demonstrating a level of successful exoerythrocytic merozoite egress was taking place in the presence of 100 nM WM382. **H.** Averaged liver parasite staging data from 3 independent experiments with mean ± SD shown. Data in (F) represent the mean ± SD from technical replicates with analysis by t-test (top row), One-way ANOVA with Dunnett's multiple comparison test (middle rows) and Fisher's exact test (bottom row). *$p<0.05$, **$p<0.01$, ***$p<0.001$; scale bar 20 μm. This analysis shows that there is an incomplete inhibitory effect of WM382 on egress of *P. berghei* exoerythrocytic merozoites from hepatocytes.

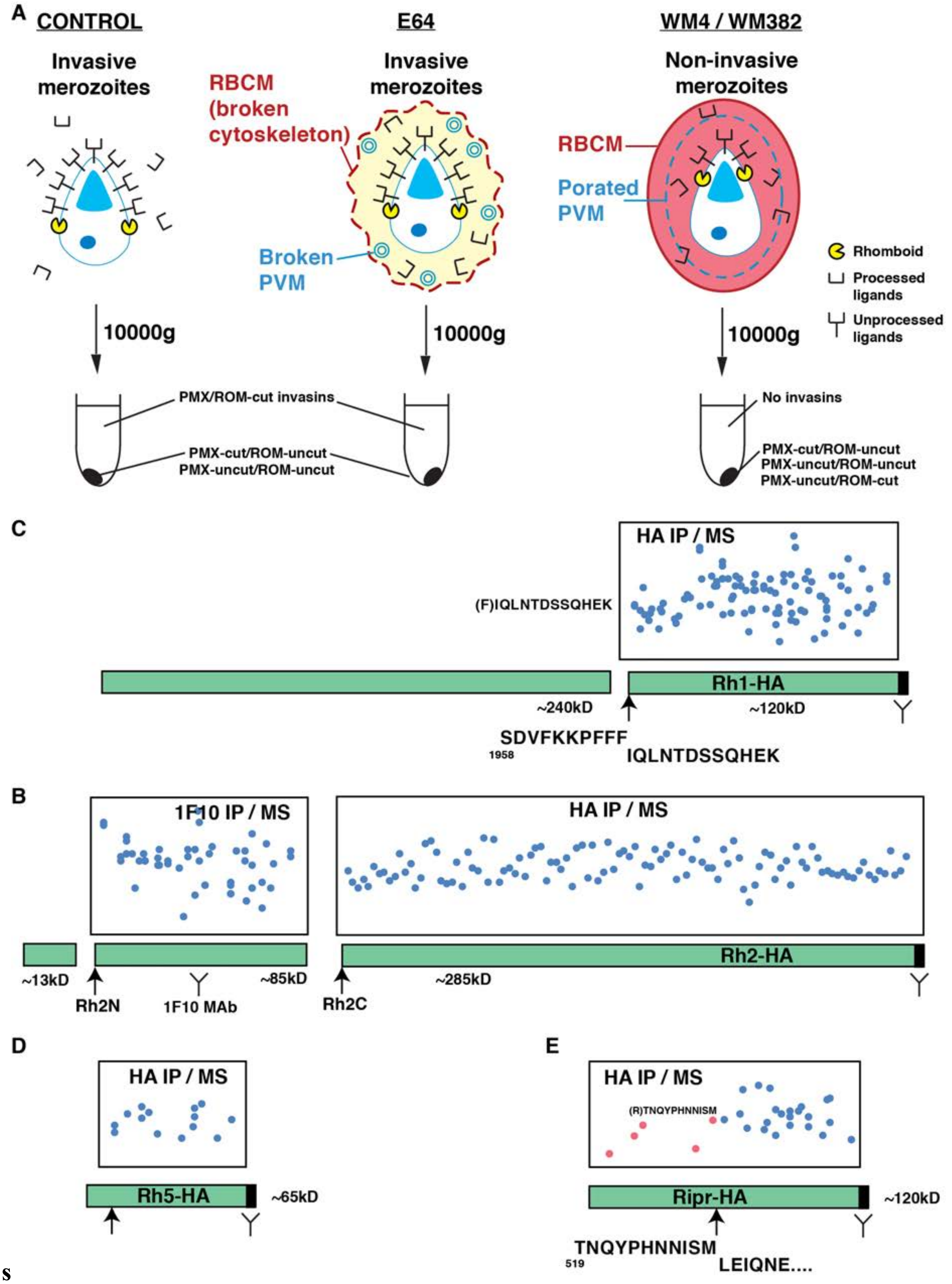

s

Supplementary Figure 7. Related to Figure 6. Model of drug treated schizonts and mass spectrometry analysis of affinity purified *P. falciparum* parasites. A. Untreated (control) or drug-treated schizont parasites were purified using magnetic columns and then placed in 96 well trays with medium and incubated for a further 16 hr. Merozoites egress from untreated control schizonts (left of panel) with release of PMX and PMIX processed apical organelle contents as well as shedding of proteins from the surface due to the action of SUB2 and

rhomboid (ROM) protease. This releases the ligands into the supernatant and proteins remaining in the merozoite are centrifuged in the pellet. E64-treated schizonts (middle of panel) have a broken parasitophorous vacuole (PVM) but an intact – but leaky- red blood cell membrane (RBCM), as a result of blocking of SERA6 activity, consequently, ligands are able to move through to the supernatant including ROM and PMX- cleaved proteins. Proteins uncleaved by PMX and ROM are not released from the merozoite and are centrifuged to the pellet. WM4 or WM382 schizonts have an intact RBCM, since SUB1 (which is required to activate multiple proteins including not only SERA6, but also possibly pore-forming proteins and phospholipases implicated in egress of other parasite stages) (Thomas *et al.*, 2018) is not proteolytically active when it has not been cleaved by PMX, so that most proteins are retained and centrifuged to the pellet. PMX or PMIX inhibit processing of some proteins and these are detected in the pellet in immunoblots. **B.** Rh2a/b processed proteins were affinity purified with monoclonal antibody 1F10 and Rh2b with anti-HA antibodies and specific protein bands separated by SDS/PAGE and trypsin digested and analysed by mass spectrometry. The dots refer to individual peptides identified. The arrows refer to the site of the PMX consensus cleavage sequence for both Rh2N and Rh2C peptides that are cleaved by recombinant PMX (Fig. 2 and Fig. S4). **C.** The Rh1120 kDa protein band was affinity purified with anti-HA antibodies and separated by SDS/PAGE, trypsin and chymotrypsin digested and analysed by mass spectrometry. The dots refer to individual peptides identified. The arrow refers to the site of the cleavage identified from both a semi-tryptic and a semi-chymotryptic peptide by mass spectrometry and corresponds to the PMX consensus cleavage sequence for Rh1. The amino acid position in the Rh1 protein is indicated below the peptide. **D.** Rh5 was affinity purified with anti-HA antibodies and the 45 kDa processed protein separated by SDS/PAGE, trypsin digested and analysed by mass spectrometry. The dots refer to individual peptides identified. The arrow refers to the approximate site of the cleavage identified by mass spectrometry and corresponds to the PMX consensus cleavage sequence for Rh5. **E.** The Ripr 60 kDa processed protein band was affinity purified with anti-HA antibodies and separated by SDS/PAGE, trypsin digested and analysed by mass spectrometry. The dots refer to individual peptides identified. The two processed bands of 60 kDa co-migrate so that there are some peptides from the N-terminus from which a semi-tryptic peptide was identified and shown with an arrow and corresponds to the PMX consensus cleavage sequence for Ripr. The amino acid position in the Rh5 protein is indicated below the peptide.

References

Pino, P., Caldelari, R., Mukherjee, B., Vahokoski, J., Klages, N., Maco, B., *et al*. (2017). A multistage antimalarial targets the plasmepsins IX and X essential for invasion and egress. *Science* **358,** 522-528.

Thomas, J.A., Tan, M.S.Y., Bisson, C., Borg, A., Umrekar, T.R., Hackett, F., *et al*. (2018). A protease cascade regulates release of the human malaria parasite *Plasmodium falciparum* from host red blood cells. *Nat Microbiol* **3,** 447-455.
