# Supplementary material for: Dual Plasmepsin-Targeting Antimalarial Agents Disrupt Multiple Stages of the Malaria Parasite Life Cycle
Source: Cell Host Microbe. 2020 Apr 8;27(4):642–658.e12. doi: 10.1016/j.chom.2020.02.005 (PMC7146544; doi:10.1016/j.chom.2020.02.005)
Supplement: Table S1. Oligonucleotides [file mmc2.docx]

**OLIGONUCLETIDES TABLE S1**

| **Oligonucleotide Sequence** | **Description** | **Primer name** |
| --- | --- | --- |
| TAAGTATATAATATTatcttttaatagtgtcccaaGTTTTAGAGCTAGAA | PMIX guide | PF1 |
| TTCTAGCTCTAAAACttgggacactattaaaagatAATATTATATACTTA | PMIX guide | PF5 |
| GAATTCAAATCGAATATTTCTTTCGTGTCG | PMIX 3’ flank | PF3 |
| CTGCAGATATCTCATAAACGTTGCATTCCC | PMIX 3’ flank | PF4 |
| CCGCGGAATAAAGCACAAGATATATCTGACC | PMX 5’ flank | TT967 |
| GGATCCTGATCCAAATACTATGTGTAAATTTTTTTC | PMX 5’ flank | TT968 |
| GAATTCAGATACATTTATGTTAGGAAAG | PMX 3’ flank | TT969 |
| CTGCAGATAGTTTTGTTCAGTACATGCAG | PMX 3’ flank | TT970 |
| TAAGTATATAATATTggttctatatcaggttctgtGTTTTAGAGCTAGAA | PMX guide | TT979 |
| TTCTAGCTCTAAAACacagaacctgatatagaaccAATATTATATACTTA | PMX guide | TT980 |
| CATCATGAGTCTCTAAAATTAGGGGACG | PMX Southern | PMXS1 |
| CACTCTCTACTAATCCAAAAGTCTG | PMX Southern | PMXS2 |
| CAAGAAGCAGTTCCTGAGGAAA | EBA175 Southern | 175S1 |
| CCCAGAATTTCCCCCCCGATCCTG | EBA175 Southern | 175S2 |
| TAAGTATATAATATTgatagtgaaaaagcatatggGTTTTAGAGCTAGAA | RON3 guide | TT927 |
| TTCTAGCTCTAAAACccatatgctttttcactatcAATATTATATACTTA | RON3 guide | TT928 |
| CTGCAGCCAAAAGCAGATATTATATCTTTATATAAAATTGTGG | RON3 3’ flank | TT925 |
| ACTAGTATTCTTCTAACGTCAATACTGG | RON3 3’ flank | TT926 |
| GCGGCCGCAAAATATAACTAAACCATCAGATC | RON3 5’ flank | TT923 |
| TATTGTATCTGGACTAACCATGG | RON3 5’ flank | TT924 |
| TAAGTATATAATATTaatgctagaggttatagaggGTTTTAGAGCTAGAA | MTRAP guide | TT774 |
| TTCTAGCTCTAAAACcctctataacctctagcattAATATTATATACTTA | MTRAP guide | TT775 |
| CTGCAGAATATAGAGGGTGATAATATAAC | MTRAP 3’ flank | TT768 |
| ACTAGTTATTCTTGCTCATTCGAGTGCCC | MTRAP 3’ flank | TT769 |
| GCGGCCGCCGTGCTATATAAACATATTTACG | Rh5 5’ flank | TT683 |
| TCAGATTTATCATCGATTTC | Rh5 5’ flank | TT684 |
| GAATTCGAAGATAGTATACAAGATAC | Rh5 3’ flank | TT681 |
| ACTAGTGACAGATGATGAAACCGAAGAGGAATAAATAAAGA  ATATTCATTTGACAT | Rh5 3’ flank | TT919 |
| TAAGTATATAATATTgacagatgatgaaaccgaagGTTTTAGAGCTAGAA | Rh5 guide | TT685 |
| TTCTAGCTCTAAAACcttcggtttcatcatctgtcAATATTATATACTTA | Rh5 guide | TT686 |
| GCGGCCGCTTTTTGTATCTTACAGCTGCTCC | Ripr 5’ flank | TT934 |
| CATTCACCGCGGGATGATCTATAATAATGTTC | Ripr 5’ flank | TT935 |
| GAATTCAAATGTGTTTTAGAAGATAAATGTG | Ripr 3’ flank | TT936 |
| ACTAGTTACATGTTTGATGATCTACTTGG | Ripr 3’ flank | TT937 |
| TAAGTATATAATATTcaaggtcatgtagctgtcaaGTTTTAGAGCTAGAA | Ripr guide | TT938 |
| TTCTAGCTCTAAAACttgacagctacatgaccttgAATATTATATACTTA | Ripr guide | TT939 |
| TTGAACACATGGCAAGGAAA | Aldolase forward | PfAldoF |
| ATTTTCACCACCTGCACCTC | Aldolase rev | PfAldoR |
| TTGAAGAATGCCTTTTCATTTT | PMX Amplification forward | PMXAmpF |
| TGGTTTAGGGATGAGGGTTA | PMX Amplification Rev | PMXAmpR |
| CGGGTCAGAAAGAATGGTGT | DiCre forward | DiCreF |
| TGATTTCAGGGATGGACACA | DiCre Rev | DiCreR |

* 20 nucleotide guide sequences are shown in lower case
