# Supplementary material for: Dual Plasmepsin-Targeting Antimalarial Agents Disrupt Multiple Stages of the Malaria Parasite Life Cycle
Source: Cell Host Microbe. 2020 Apr 8;27(4):642–658.e12. doi: 10.1016/j.chom.2020.02.005 (PMC7146544; doi:10.1016/j.chom.2020.02.005)
Supplement: Document S2. Article plus Supplemental Information [file mmc5.pdf]

Clinical and Translational Report
Cell Host & Microbe

Dual Plasmepsin-Targeting Antimalarial Agents Disrupt Multiple Stages of the Malaria Parasite Life Cycle

Graphical Abstract

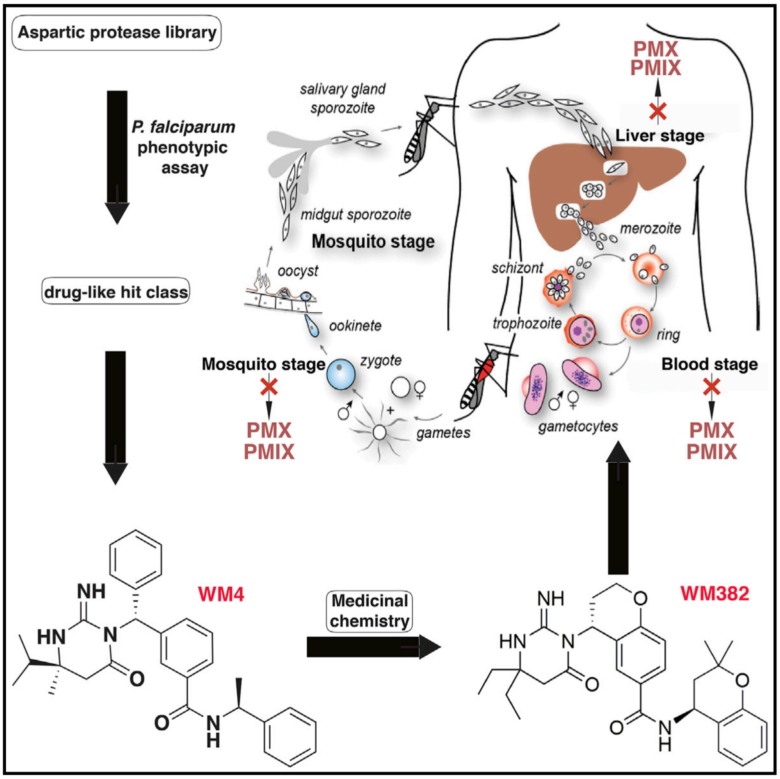

Authors

Paola Favuzza, Manuel de Lera Ruiz, Jennifer K. Thompson, ..., John A. McCauley, David B. Olsen, Alan F. Cowman

Correspondence

david_olsen@merck.com (D.B.O.), cowman@wehi.edu.au (A.F.C.)

In Brief

We describe inhibitors of essential aspartic proteases from malaria parasites that block multiple life cycle stages. PMIX and PMX are master modulators processing proteins required for invasion, development, and egress. Administration of WM382 cured mice of malaria infection, showing that these inhibitors are promising candidates for malaria treatment and prevention.

Highlights

- Specific compounds against *P. falciparum* Plasmepsin IX and X were identified
- PMIX and PMX are modulators of parasite proteins for egress, invasion, and development
- Anti-PMIX and anti-PMX compounds inhibit liver, blood, and mosquito stages of *Plasmodium*
- One compound, WM382, can clear mouse models of *P. berghei* and *P. falciparum* parasites

Favuzza et al., 2020, Cell Host & Microbe *27*, 642–658
April 8, 2020 © 2020 The Authors. Published by Elsevier Inc.
https://doi.org/10.1016/j.chom.2020.02.005

Dual Plasmepsin-Targeting Antimalarial Agents Disrupt Multiple Stages of the Malaria Parasite Life Cycle

Paola Favuzza,[1,2,5] Manuel de Lera Ruiz,[3,5] Jennifer K. Thompson,[1] Tony Triglia,[1] Anna Ngo,[1] Ryan W.J. Steel,[1,2] Marissa Vavrek,[3] Janni Christensen,[1,2] Julie Healer,[1,2] Christopher Boyce,[3] Zhuyan Guo,[3] Mengwei Hu,[3] Tanweer Khan,[3] Nicholas Murgolo,[3] Lianyun Zhao,[3] Jocelyn Sietsma Penington,[1] Kitsanapong Reaksudsan,[1,2] Kate Jarman,[1] Melanie H. Dietrich,[1,2] Lachlan Richardson,[1,2] Kai-Yuan Guo,[1,2] Sash Lopaticki,[1] Wai-Hong Tham,[1,2] Matthias Rottmann,[4] Tony Papenfuss,[1,2] Jonathan A. Robbins,[3] Justin A. Boddey,[1,2] Brad E. Sleebs,[1,2] Hélène Jousset Sabroux,[1,2] John A. McCauley,[3] David B. Olsen,[3,6,*] and Alan F. Cowman[1,2,6,7,*]

[1]The Walter and Eliza Hall Institute of Medical Research, Parkville, VIC 3052, Australia
[2]University of Melbourne, Melbourne, VIC 3010, Australia
[3]Merck & Co., Inc., 770 Sumneytown Pike, West Point, PA 19486, USA
[4]Swiss Tropical and Public Health Institute, Basel 4002, Switzerland
[5]These authors contributed equally
[6]Senior author
[7]Lead Contact
*Correspondence: david_olsen@merck.com (D.B.O.), cowman@wehi.edu.au (A.F.C.)
https://doi.org/10.1016/j.chom.2020.02.005

SUMMARY

Artemisin combination therapy (ACT) is the main treatment option for malaria, which is caused by the intracellular parasite *Plasmodium.* However, increased resistance to ACT highlights the importance of finding new drugs. Recently, the aspartic proteases Plasmepsin IX and X (PMIX and PMX) were identified as promising drug targets. In this study, we describe dual inhibitors of PMIX and PMX, including WM382, that block multiple stages of the *Plasmodium* life cycle. We demonstrate that PMX is a master modulator of merozoite invasion and direct maturation of proteins required for invasion, parasite development, and egress. Oral administration of WM382 cured mice of *P. berghei* and prevented blood infection from the liver. In addition, WM382 was efficacious against *P. falciparum* asexual infection in humanized mice and prevented transmission to mosquitoes. Selection of resistant *P. falciparum in vitro* was not achievable. Together, these show that dual PMIX and PMX inhibitors are promising candidates for malaria treatment and prevention.

INTRODUCTION

Several hundred million infections and 430,000–618,700 deaths each year occur because of malaria: the most lethal disease is caused by *Plasmodium falciparum* with the major burden of mortality and morbidity in Africa (Weiss et al., 2019). A relapsing form of malaria, caused by *P. vivax*, is a major problem outside Africa (Battle et al., 2019). *P. knowlesi* malaria is found in Southeast Asia (Singh et al., 2004). Artemisinin combination therapy (ACT) is the mainstay for treatment and control of malaria. However, the decreasing efficacy of ACT highlights the need for discovery of new drugs with novel mechanisms of action that can be used to control, eliminate, and eradicate malaria (Menard and Dondorp, 2017).

A series of proteolytic events are essential for egress from and invasion of host cells by *P. falciparum* (Alaganan et al., 2017). The subtilisin-like protease subtilisin 1 (SUB1) plays a key role and is involved in remodeling the merozoite surface and egress from the host erythrocyte (Collins et al., 2017; Silmon de Monerri et al., 2011). SUB1 processes the serine-repeat antigens 5 and 6 (SERA5 or 6), that are also involved in host cell egress (Collins et al., 2017; Thomas et al., 2018). SUB2 is a sheddase releasing proteins, including MSP1, AMA1, and PTRAMs, from the merozoite surface during invasion (Olivieri et al., 2011). Although the downstream events mediated by these subtilisins are well described, there remains an incomplete understanding of how they are activated.

Erythrocyte invasion in malaria involves two essential protein families, *P. falciparum* reticulocyte-binding protein homologs (PfRhs) and erythrocyte binding-like (EBL) proteins (Lopaticki et al., 2011). Engagement of Rh and EBL proteins with receptors initiates a phosphorylation cascade leading to increased deformability of the erythrocyte membrane (Koch et al., 2017; Sisquella et al., 2017). Additionally, receptor ligation is important for signaling downstream for invasion (Singh et al., 2010; Tham et al., 2015). Following these events, Rh5, in complex with CyRPA and Ripr, binds to basigin on the erythrocyte membrane (Crosnier et al., 2011) and is involved in formation of a membrane pore through which Ca^{2+} can flow (Volz et al., 2016; Weiss et al., 2015; Wong et al., 2019). EBL and Rh proteins are processed at the parasite membrane during invasion by a rhomboid (ROM) protease releasing them for movement of the merozoite into the erythrocyte (Baker et al., 2006).

 © 2020 The Authors. Published by Elsevier Inc.
This is an open access article under the CC BY license (http://creativecommons.org/licenses/by/4.0/).

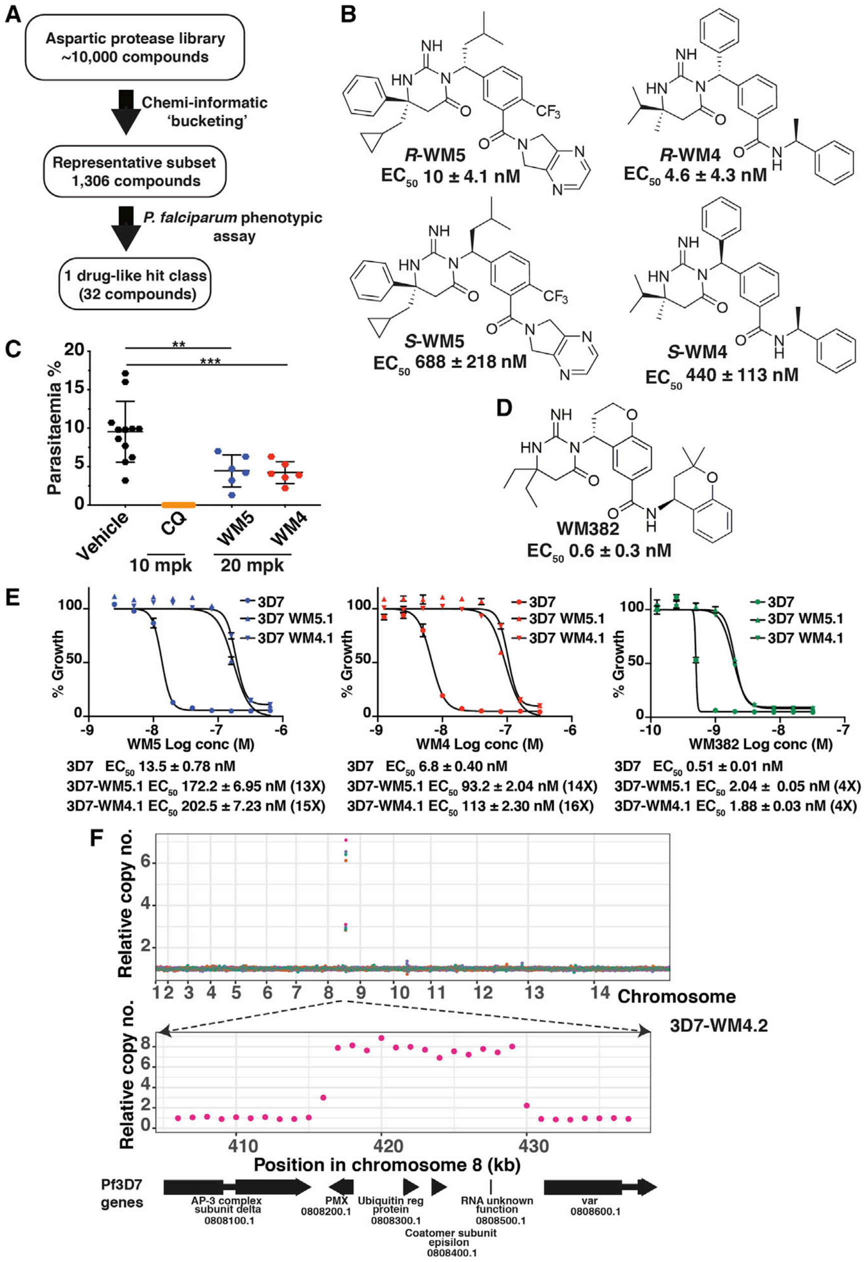

Figure 1. Identification of Compounds that Inhibit *P. falciparum*

(A) Screen using an aspartyl protease compound library to identify inhibitors of *P. falciparum* growth.

(B) Chemical structure of compounds and EC_{50}.

(C) WM5 and WM4 suppress *P. berghei* infection. Two independent experiments; n = 6, mean ± SD. $^{**}p < 0.005$, $^{***}p < 0.0005$.

(D) Structure and EC_{50} of *R*-WM382.

(E) Growth curves for WM5 and 4 resistant *P. falciparum*. Experiments in triplicate, mean ± SEM.

(F) Genome of *P. falciparum* WM5 and four resistant lines. Top: copy number (10 kb bins) of 3D7-WM4.2 compared to 3D7 for chromosomes, four replicates in four colors. Bottom: 30 kb region (1 kb bins). Gene numbers found at PlasmoDB: https://plasmodb.org/.

Because of the increasing spread of ACT drug resistance, development of new antimalarials is a priority. A drug regimen acting on novel targets at multiple life cycle stages would enhance its utility and longevity for malaria elimination, because there is a reduced likelihood of parasites with pre-existing resistance mutations being present in the population. In the antimalarial drug space, the essential *P. falciparum* aspartic proteases, plasmepsins IX and X (PMIX and PMX), are potential targets since inhibitors block parasite egress and invasion and prevent maturation of rhoptry and micronemal proteins required for this process (Nasamu et al., 2017; Pino et al., 2017).

Here, an orally bioavailable lead compound with potent *in vitro* and *in vivo* activity against malaria was discovered along with selective PMX and dual PMIX/X inhibitors. Using these compounds, we have identified previously unknown substrates of PMIX/PMX crucial for parasite infection.

RESULTS

Hit Compounds Identification

An aspartic protease inhibitor library was screened to identify hit compounds targeting *P. falciparum* (Figure 1A). This identified 32 compounds that inhibited growth of *P. falciparum*. The two most potent screening hits, *R,S*-WEHI/Merck5 (WM5) and *R,S*-WEHI/Merck4 (WM4) (Figure 1B), consisted of two stereoisomers and each enantiomer was purified to determine their potency (Figures 1B and S1). This showed *R*-WM5 and *R*-WM4 were active enantiomers with an EC_{50} of 10 nM and 4.6 nM respectively (Figures 1B and S1). *RS*-WM5 and *RS*-WM4 were intraperitoneally administered to mice to determine *in vivo* activity against *P. berghei* infection (Figure 1C). Both compounds had partial efficacy and suppressed *P. berghei* parasitemia; however, they did not have desirable pharmacokinetic attributes, and a molecular model-guided program was mounted to derive compounds with required drug-like properties. Optimization studies resulted in the identification of WM382, a compound inhibiting *P. falciparum* and *P. knowlesi* growth with EC_{50}s of 0.6 nM and 0.2 nM, respectively (Figures 1D and S1).

WM4, WM5, and WM382 Target Plasmepsin X

The target of these compounds was identified by selecting *P. falciparum* for resistance to WM4 and WM5. Five independent lines for both WM5 (3D7-WM5.1, 2, and 3) and WM4 (3D7-WM4.1 and 2) were selected and two examples are shown: 3D7-WM5.1, which was 13-fold resistant compared with 3D7 (EC_{50} 172 nM), and 3D7-WM4.1 (EC_{50} 93 nM), which was 14-fold

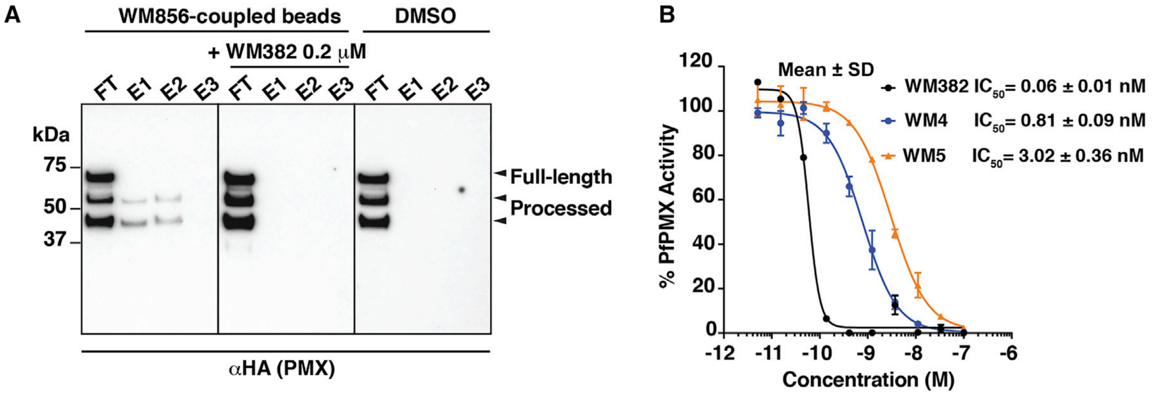

C

	Ki		Fold Selectivity	EC_{50}	
	PMV (μM)	PMX (nM)	PMX v PMV	*P. falciparum* (nM)	HepG2 (μM)
WM382	13.4 ± 0.62	0.035 ± 0.008	382,857	0.6 ± 0.3	24.8 ± 2.2
WM4	19.8 ± 0.5	0.5 ± 0.2	36,000	7.4 ± 0.8	14.2 ± 2.3
WM5	3.3 ± 0.54	1.9 ± 0.6	1,755	9.3 ± 2.7	17.9 ± 2.0

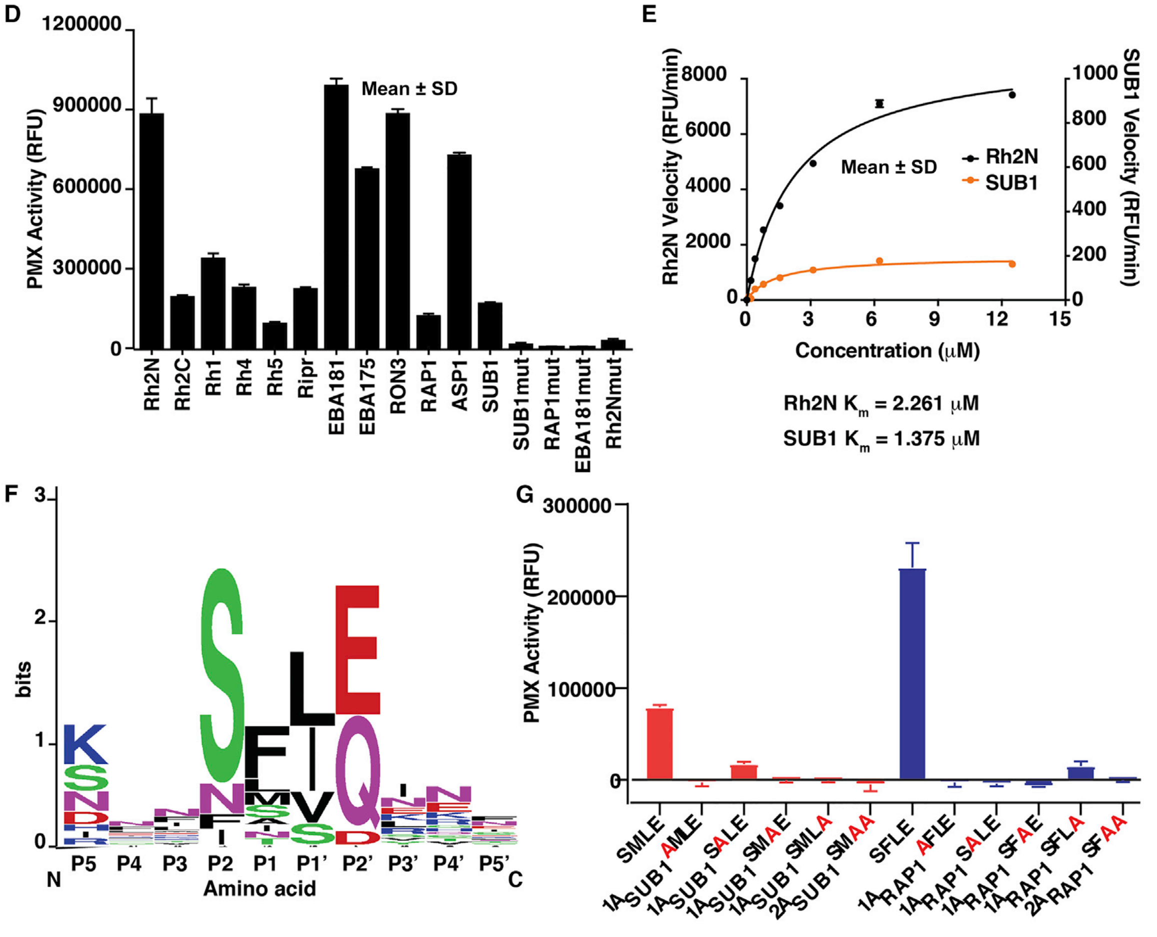

Figure 2. Enzyme Activity, Selectivity, and Substrate Specificity of rPMX

(A) PMX-HA immuno-detection after pull-down with WM856. FT, flow through; E1, E2, E3, elution fractions. Left: unbound and eluted proteins from WM856-coupled beads. Center: with 0.2 μM WM382. Right: Uncross-linked beads.

(B) IC_{50} for WM382, WM4, and WM5 inhibition of PMX with Rh2N peptide. Mean ± SD.

(C) $K_{i,}$ for WM382,WM4, and WM5 with PMV and PMX, EC_{50} for *P. falciparum* and HepG2 cells. Mean ± SD (three experiments).

(legend continued on next page)

resistant (Figure 1E). The selected lines exhibited cross-resistance between WM5, WM4, and WM382 suggesting WM5 and WM4 are hitting the same target(s) and/or had the same mechanism of resistance (Figures 1E and S1). WM382 showed a low level of cross-resistance with both 3D7-WM5.1 and 3D7-WM4.1 (4-fold) (Figure 1E), and this, together with the compound's anti-parasite potency, suggested that WM382 was inhibiting an additional target(s).

Genome sequencing of the resistant parasites did not identify any point mutations. However, copy number analysis revealed an amplification event on chromosome 8 (Figure 1F). The smallest amplified chromosome unit (14 kb) occurred in 3D7-WM4.2 and included the gene encoding the aspartic protease PMX (PlasmoDB: PF3D7_0808200) (Figure 1F). The breakpoints of each amplification were established, and the copy number of *PMX* ranged from 6 to 10 with protein expression also increased in the resistant lines (Figure S1). These data suggested PMX was the direct target and/or involved in the mechanism of resistance for both WM5 and WM4.

WM382, WM4, and WM5 Are Not Cross-Resistant to Other Antimalarial Drugs

WM382 represents a novel class of antimalarials, and we determined whether parasites selected for resistance to the parent compounds displayed cross-resistance to known antimalarials. The EC_{50} of WM4 and WM382 for *P. falciparum* strains resistant to chloroquine, mefloquine, artemisinin, and atovaquone was determined (Figure S2). The resistance of these parasite strains to the respective antimalarials was confirmed, and no cross-resistance to either WM4 or WM382 was observed (similar results were obtained for WM5). Therefore, the potencies of WM4, WM5, and WM382 were not affected by pre-existing resistance mutations in drug resistant parasites.

While it was possible to select resistance to WM4 and WM5, these parasites had a selective growth disadvantage compared with the parental line (Figure S3). Additionally, ''time to resistance'' selections were performed for WM4 and WM382 and compared with atovaquone as control (Figure S3) (Ding et al., 2012). Parasites resistant to atovaquone were selected, but no parasite recrudescence was observed for up to 90 days when using WM4 or WM382 (Figure S3). Currently, selection with WM382 has not yielded resistant parasites consistent with WM382 exerting its action through more than one target.

WM4, WM5, and WM382 Inhibit *P. falciparum* PMX

To determine whether WM4, WM5, and WM382 bound directly to PMX, an azide-functionalized analog of WM382 (WM856: EC_{50} 1.1 nM; Figure S4) was synthesized. PMX from 3D7-PMX-hemagglutinin (HA) parasites was eluted from WM856-coupled beads, but not in the presence of WM382 as a competitor, indicating specific PMX binding (Figures 2A and S4). These results were consistent with PMX being the direct target of WM856, WM4, WM5, and WM382.

To determine whether WM382, WM4, and WM5 inhibited protease activity, recombinant PMX protein (rPMX) was expressed and purified (Figure S4). rPMX was obtained in a processed form comprising the catalytic (51 kDa and 44 kDa, respectively) and pro-domain (16 kDa) as shown by proteomic analysis, and this cleavage was inhibited by WM4 or WM382. To measure rPMX protease enzyme activity, a peptide substrate from the *P. falciparum* invasion ligand Rh2a/b was used (see below). WM382, WM4, and WM5 potently inhibited rPMX activity, with an IC_{50} of 0.06 nM, 0.81 nM, and 3.02 nM, respectively (Figure 2B). The rank order of potency for WM382, WM4, and WM5 against the enzyme was the same as for inhibition of *P. falciparum* growth (Figure 2C). The selectivity of WM5, WM4, and WM382 was analyzed against enzyme activity of the *P. falciparum* aspartic protease, PMV (Hodder et al., 2015). This showed WM382 was more selective for PMX than PMV (382,857-fold) compared with WM4 (36,000-fold) and WM5 (1,755-fold), consistent with PMX being a specific target of these inhibitors.

To identify substrates of PMX, a bioinformatic search was performed using a consensus sequence and substrates synthesized and assayed for cleavage (Figures 2D and S4). This suggested the Rh and EBL proteins, including the essential protein Rh5 and its complex partner Ripr (Wong et al., 2019), and RON3 (Low et al., 2019) were processed by PMX. While cleavage of the substrates was clear compared with a range of corresponding mutant peptides, there was a large variation in efficiency. The rate of cleavage of the Rh2N peptide was substantially faster than that of SUB1 (Figure 2E).

While some PMIX (PlasmoDB: PF3D7_1430200) and PMX substrates, such as those of SUB1, MSP1, and RAP1, have been previously identified, the cleavage position within the tetrapeptide was unknown (Nasamu et al., 2017; Pino et al., 2017). The cleavage products of the peptide substrates SUB1, RAP1, Rh2N, RON3, EBA175, and EBA181 were analyzed by mass spectrometry (Figure S5) to show PMX cleaves after the P1 and before the P1′ position (Figure 2F). Alanine replacement of the tetrapeptide showed each position was important (Figure 2G), suggesting a constrained consensus between PMIX and X. Interestingly, PMX cleaves RAP1 and apical sushi protein (ASP) peptides, which are known PMIX substrates (Pino et al., 2017), suggesting that these proteases have similar substrate specificities and other factors, such as the subcellular localization of the protease, are important determinants for processing.

WM382 Has Dual Activity against PMIX and PMX

To confirm that PMX was a target of WM382 and WM4, and to test whether WM382 also targets PMIX, we generated *P. falciparum* lines to knockdown expression of PMIX and PMX using the GlcN inducible *glmS* ribozyme (Prommana et al., 2013). The level of PMIX and X expression was decreased in 3D7-PMX-HA and 3D7-PMIX-HA, respectively, in the presence of GlcN (Figure 3A). The EC_{50} of WM4 and WM382 was significantly decreased when PMX expression was decreased

(D) rPMX activity for peptide cleavage. Mutant controls, mut; RFU, relative fluorescence units. Mean ± SD.

(E) Rate of enzyme activity and K_m of rPMX for Rh2a/b and SUB1. Mean ± SD.

(F) Sequence logo of amino acids P5-P5′ positions.

(G) Cleavage of peptides containing SUB1 (red) and RAP1 (blue) sequences and alanine substitutions by rPMX. Mean ± SD.

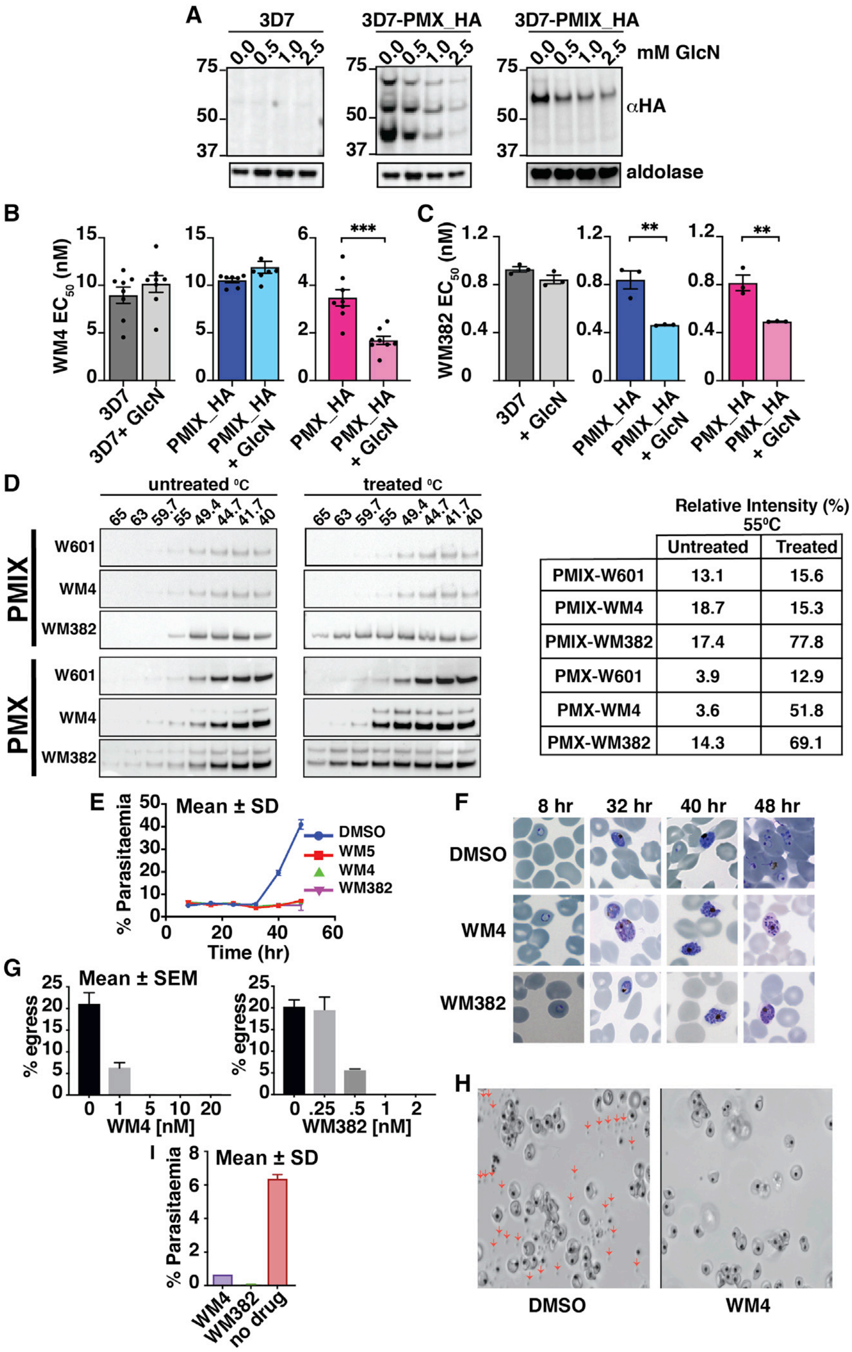

	Untreated	Treated
PMIX-W601	13.1	15.6
PMIX-WM4	18.7	15.3
PMIX-WM382	17.4	77.8
PMX-W601	3.9	12.9
PMX-WM4	3.6	51.8
PMX-WM382	14.3	69.1

Figure 3. WM382 and WM4 Target Engagement, Parasite Growth, Egress, and Invasion

(A) Knockdown of PMIX and PMX expression in *P. falciparum* using *glms* ribozyme and glucosamine (GlcN). 3D7, 3D7-PMX_HA, and 3D7-PMIX_HA treated with GlcN and probed with anti-HA.

(B) EC_{50} for WM4 with *P. falciparum* in presence (light) and absence (dark) of 2.5 mM GlcN. Mean ± SD (eight experiments).

(C) WM382 EC_{50} for *P. falciparum* in presence (light) and absence (dark) of 2.5 mM GlcN. Mean ± SE (five experiments).

(D) CETSA with WM4 and WM382. Immuno-blots probed with αHA in 3D7-PMIX_HA and 3D7-PMX_HA. Right: relative intensity at 55°C.

(E) Time of parasite killing for WM5, WM4, and WM382 in blood stage. DMSO, control. Mean ± SD.

(F) *P. falciparum*-infected erythrocytes with DMSO, WM4, or WM382.

(G) WM4 and WM382 block merozoite egress (percentage). Mean ± SEM.

(H) Stills of schizonts visualizing egress (20 nM WM4). Arrows, free merozoites. Supplemental Information includes Video S1 (control with no drug) and Video S2 (plus 20 nM WM4).

(I) Parasites grown in WM4 or WM382 are shown and percentage parasitemia. Mean ± SD.

(Figure 3B). However, there was no significant change in EC_{50} for WM4 when expression of PMIX was decreased (WM5 gave similar results to WM4; data not shown). In contrast, both PMX-HA and PMIX-HA parasites were significantly more sensitive to WM382 when expression of PMX or PMIX was decreased (Figure 3C). This suggests WM4 targets PMX while WM382 acts as a dual inhibitor of PMIX and X.

Additional data confirming that WM382 engages with both PMIX and X, while WM4 binds selectively to PMX, were provided by cellular thermal shift assays (CETSA) (Martinez Molina and Nordlund, 2016). The compound W601, which binds to and inhibits the aspartic protease PMV (Nguyen et al., 2018), had no effect on the thermal stability of either PMX or PMIX (Figure 3D). Similarly, WM4 had no effect on the thermal stability of PMIX, while it stabilized PMX. In contrast, PMIX and X were significantly more thermostable with WM382, consistent with this compound engaging both targets. Therefore, WM4 binds PMX, whereas WM382 has dual binding activity for PMIX and X.

WM4, WM5, and WM382 Block Egress and Treated Merozoites Are Incapable of Invading Erythrocytes

To determine the point at which these compounds block blood-stage growth, WM382 was added to early rings and development followed (Figures 3E and 3F). Both parasite development and growth continued normally for the control, however, WM5-, WM4-, and WM382-treated parasites arrested at late schizont stage. Previously, it has been shown compounds inhibiting PMX block egress of merozoites from infected erythrocytes (Nasamu et al., 2017; Pino et al., 2017). Both WM4 and WM382 potently inhibited egress of merozoites from the erythrocyte compared with control (Figures 3G and 3H).

It has been shown that inhibitors of PMIX and PMX render merozoites incapable of invading erythrocytes (Nasamu et al., 2017; Pino et al., 2017). To test whether WM4 and WM382 affected merozoite invasion, they were added to schizont stage parasites and merozoites were released by mechanical rupture (Figure 3I).

Addition of 40 nM WM4 resulted in merozoites significantly less able to invade, while with 2.5 nM of WM382 the merozoites were unable to invade erythrocytes. Therefore, both WM382 and WM4 inhibit egress of parasites from schizonts and result in the development of merozoites unable to invade erythrocytes.

WM382 Clears Mice of *P. berghei* and *P. falciparum* Blood Infection

The *in vivo* efficacy of orally delivered WM382 was evaluated in mice infected with *P. berghei*, and this compound displayed significantly greater *in vivo* efficacy than WM4 and WM5 at 20 mg/kg (mpk) using b.i.d. (*bis in die*, i.e., twice a day) treatment regimen (Figure 4A). WM382 was tested in dose-ranging experiments (b.i.d. dosage over 4 days), where parasitemia was monitored for 30 days to detect any parasite recrudescence (Figure 4B). Parasites were detectable in chloroquine-treated controls on day 10 post-infection (pi). In mice treated with 1 mpk WM382, parasites were detected from day 2 pi, while those treated with 3 mpk had detectable parasites only after day 12 with one mouse remaining parasite-free on day 30 pi (Figure 4B). Notably, all mice treated with 10 mpk and 30 mpk of WM382 remained parasite-free, indicating that they were cured (Figure 4B). Then, a daily dosing (*q.d.*; *quaque die*, once a day) of WM382 was tested and cured all mice (Figure 4C).

The *in vivo* efficacy of WM382 against *P. falciparum,* was evaluated in the humanized nonobese diabetic-severe combined immunodeficiency (NOD-*scid*) *IL2R*γ^{null} mouse model (NSG) (Angulo-Barturen et al., 2008). The immune-deficient mice were injected with *P. falciparum*-infected human erythrocytes on day 0 and, by day 3, developed a parasitemia of 0.55%± 0.09% (Figure 4D). Daily doses of chloroquine or WM382 were orally administered for 4 days (*q.d.*). The chloroquine-treated mice were cleared of parasitemia by day 7. Similarly, mice treated with WM382 were cleared of parasitemia by day 6 (30 mpk) or 7 (3 mpk and 10 mpk) (Figure 4D). WM382 blood levels were in alignment with anticipated efficacy based on pharmacokinetics of the drugs and *in vitro* potency against *P. falciparum* (Figures 4E and 4F). The *in vivo* and *in vitro* efficacy of WM382 against *P. falciparum,* including its parasitemia clearance rate, was at least equivalent to chloroquine.

WM382 Blocks Transmission of *P. falciparum* Gametocytes and *P. berghei* Liver-to-Blood Transition

To determine if WM4 and WM382 blocked transmission of *P. falciparum* gametocytes to mosquitoes, standard membrane feeding assays (SMFA) were performed (Figure 5A). Mosquitoes were fed WM4- or WM382-treated *P. falciparum* gametocytes and developing oocysts counted to determine infection rate. There was no significant reduction in oocyst numbers with WM4, however, WM382 treatment potently inhibited oocyst development. The ability of WM382 to block transmission is an important attribute for an antimalarial drug, as this could reduce both the incidence and spread of malaria.

To determine the effect of WM382 against liver-stage parasites, liver egress, and transition to blood infection, mice were infected with *P. berghei* sporozoites constitutively expressing mCherry and luciferase reporters (Pino et al., 2017; Prado et al., 2015). Animals were untreated or orally treated with 20 mpk or 100 mpk WM382 b.i.d. at 36 and 48 h post infection (hpi) (Figure 5B). There was no effect of WM382 on parasite liver infection at 52 hpi (peak infection), nor did WM382 attenuate the decline in bioluminescent signal from the liver as parasites egressed by 55 hpi (Figures 5C–5E). These data show WM382 did not kill *P. berghei* liver stages or markedly prevent liver egress *in vivo*. Using HepG2 cells *in vitro*, treatment of *P. berghei* liver stages with 100 nM WM382 from 24 hpi likewise did not reduce parasite number or size at 48 hpi (Figure S6), again indicating WM382 did not kill liver parasites. *In vitro*, liver parasite egress results in detached parasitized cells and merozoite-containing merosomes being found in the supernatant following degradation of the parasitophorous vacuole membrane (PVM) that occurs between 48 hpi and 65 hpi (Sturm et al., 2006). Treatment with 100 nM WM382 reduced detachment of parasitized HepG2 cells at 65 hpi. Merozoite formation by liver stages was normal, and although we did observe evidence for PVM breakdown and egress in 100 nM WM382 treated cultures, there was an accumulation of larger, mature liver parasites containing merozoites without PVM degradation (Figure S6). These data demonstrate liver-stage parasites develop normally and egress with WM382, albeit at reduced rates *in vitro*.

We next sought to determine if treatment of liver-stage parasites with WM382 would affect viability of exoerythrocytic merozoites and protect mice from developing a blood infection (Figure 5B). Erythrocytic infection was detected in control, but not in WM382-treated mice using whole-body bioluminescence imaging and analysis of peripheral blood by flow cytometry (Figures 5F and 5G). There was a >4 day delay to patency ($p < 0.001$) and 50% sterile protection against blood infection in mice treated with 2 × 20 mpk (indicating a small number of viable merozoites in some mice) and 100% sterile protection in mice treated with 2 × 100 mpk ($p < 0.001$) (Figure 5H).

To exclude the possibility that failure to initiate blood infection following liver egress was because of residual activity of WM382 on blood-stage parasites, we tested whether this compound, acting on liver parasites alone, was sufficient to prevent subsequent blood infection. Supernatants containing detached cells and merosomes from infected HepG2 cultures treated with WM382 or DMSO were collected at 65 hpi, washed to remove compound, and i.v. injected into naive mice. Blood infection was initiated in mice using supernatants from DMSO and 1 nM WM382 treatment groups but not those that received 100 nM WM382 supernatants (Figure 5I). Thus WM382 was not lethal to liver-stage *P. berghei* parasites, nor could its action be explained by preventing merozoite egress alone. Rather protection from blood infection, when treatment is given at the liver stage, occurs because the exoerythrocytic merozoites are not viable and non-infectious.

To identify minimum doses of WM382 preventing liver to blood infection, we used the mosquito bite infection model and performed a dose de-escalation study. Two doses of compound were not required, since a single dose of 100 mpk WM382 given at 36 hpi was sufficient to protect all mice from developing patent infection. WM382 given at 50 mpk (36 hpi) resulted in a 5-day delay to patency with 80% sterile protection ($p < 0.001$), while 20 mpk (36 hpi) gave a 2-day delay to patency, but all mice developed blood infection ($p < 0.01$). Prophylactic WM382 treatment as a single dose of 50 mpk 4 h before mosquito bite infection did not confer

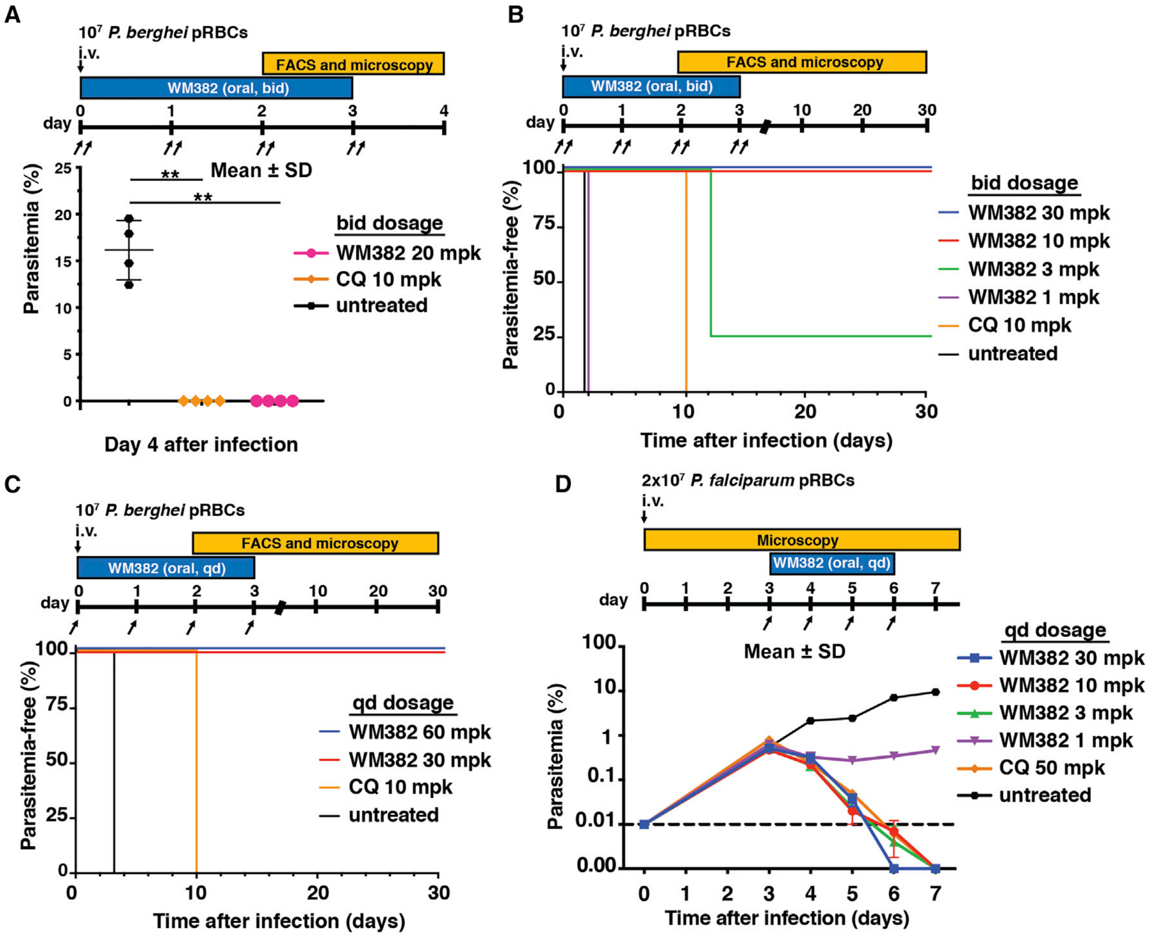

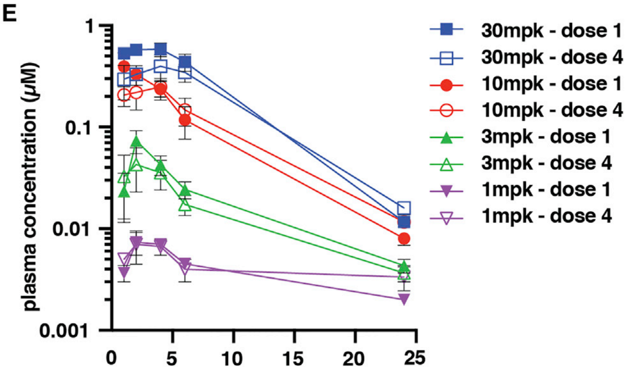

F

P. falciparum	AUC dose 1	AUC dose 4
Oral Daily Dose mg/kg	μM*hr	μM*hr
1	0.06 ± 0.03	0.10 ± 0.04
3	0.50 ± 0.18	0.37 ± 0.21
10	2.61± 1.00	2.62± 1.03
30	7.04 ± 1.53	5.17 ± 1.22

Figure 4. WM382 Cures Mice of *P. berghei* and *P. falciparum* Infection

(A) WM382 suppresses *P. berghei* infection, orally administered twice daily (b.i.d. dosage) for 4 days (n = 4). WM382 (20 mpk) compared with 10 mpk chloroquine (CQ). **$p < 0.005$: Mean ± SD.

(B) *P. berghei* infected Swiss mice are cured with oral WM382. Mice (n = 4) treated for 4 days (b.i.d.) with WM382 (mpk) or chloroquine (10 mpk).

(C) *P. berghei* infected mice (n = 4) cured with single, oral, daily doses (q.d.) of WM382.

(D) Humanized NOD-*scid* $IL2R\gamma^{null}$ mouse model shows WM382 suppresses *P. falciparum* infection. Mice (n = 3), orally treated for 4 days (q.d.) with WM382 (mpk) or chloroquine (50 mpk). Mean ± SD.

(E and F) Concentration of WM382 at 1, 2, 4, 6, and 24 h post first and last drug administration in blood from huSCID mice determined by LC-MS/MS, and (F) corresponding AUC values. Mean ± SD. Parasitemia monitored by fluorescence-activated cell sorting (FACS) and microscopy.

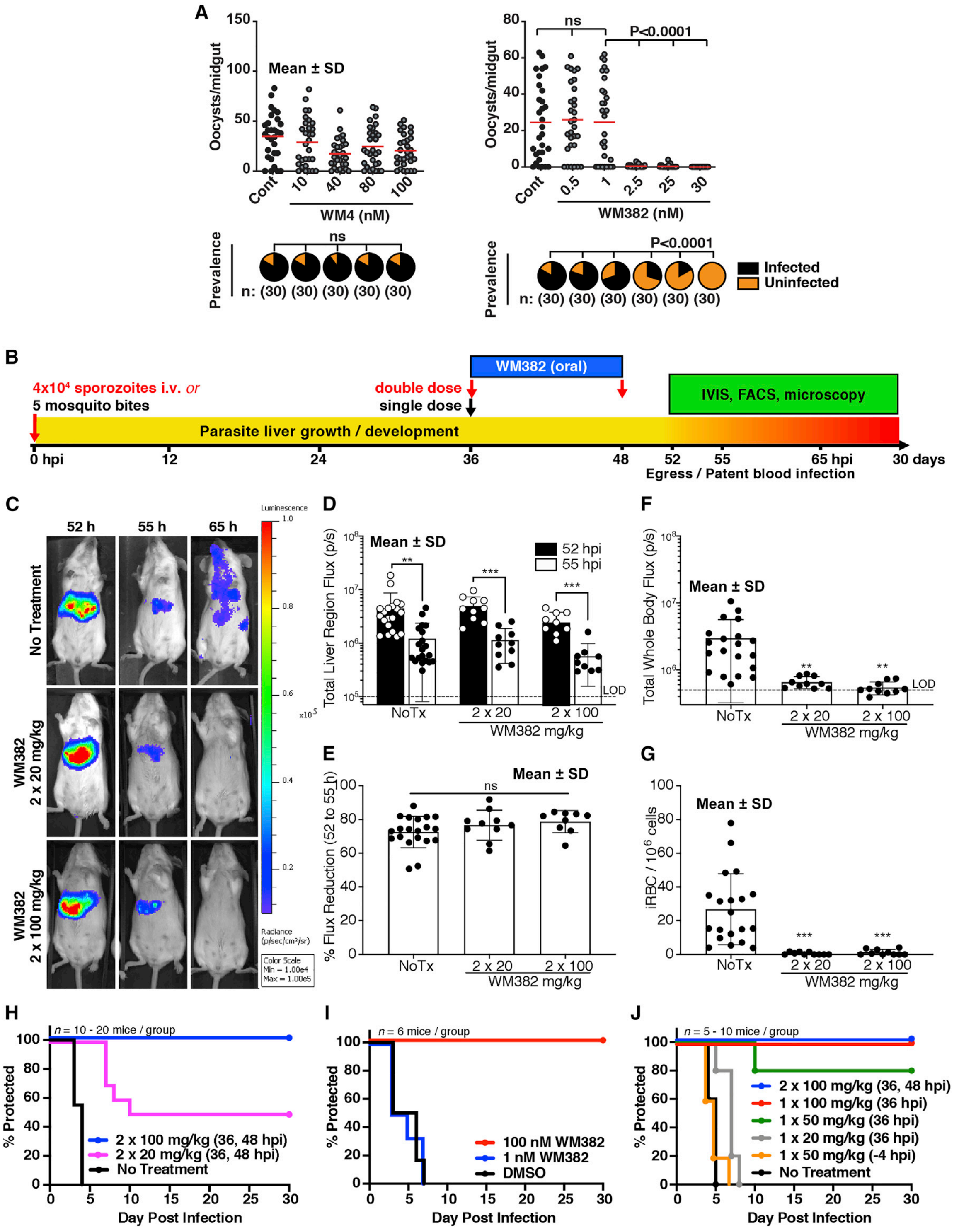

Figure 5. WM382 Prevents Transmission to Mosquitoes and Transition from Liver to Blood Infection

(A) Oocyst counts from *P. falciparum*-infected mosquito guts for gametocytes treated with WM4 or WM382. Prevalence pies, proportions of mosquitoes with oocysts (black). Mean ± SD.

(B) Mice were infected with five infectious mosquito bites (MB) or intravenous (i.v.) injection of 40,000 *Pb*mCherryLuci sporozoites. From 52 h post infection (hpi), bioluminescence measured liver infection and egress, while bioluminescence and flow cytometry measured blood infection (Figure S6).

(C) Bioluminescent images showing peak liver infection (52 hpi), liver egress (55 hpi), and blood infection (65 hpi) in WM382 treated and untreated mice.

(legend continued on next page)

any protection against subsequent blood infection (Figure 5J). Thus, a single dose of 100 mpk WM382 given during liver infection can provide protection against blood infection and disease.

WM382 Inhibits Protein Processing by PMIX and X, but WM4 Specifically Blocks PMX Function

The discovery of PMX-specific (WM4) and PMIX/X (WM382) dual inhibitors provided specific tools to investigate the function of these aspartic proteases. SERA5 is required for merozoite egress and processed by SUB1 (Figure 6A) (Pino et al., 2017). Protease inhibitor E64, which prevents schizont rupture but not SERA5 processing, was a control (Salmon et al., 2001). Following incubation with WM4 and WM382, there was an accumulation of unprocessed SERA5, confirming that SUB1 activation requires prior processing by PMX (Figure 6A). SERA5 was included for subsequent experiments as a proxy for PMX-mediated activation of SUB1 and a loading control for proteins released into the supernatant (Figure 6A).

To confirm WM382 was a dual inhibitor of PMIX and PMX, we tested its ability to inhibit cleavage of PMIX substrate RAP1 (Figure 6B) (Pino et al., 2017). RAP1 is a merozoite rhoptry protein localized to the parasitophorous vacuole (PV) after invasion and processed by PMIX and SUB1 (Pino et al., 2017). Two processed forms of RAP1 (p82 and p69) are detected in untreated and E64-treated merozoites. Because SUB1 is a substrate of PMX, processing of RAP1 to p69 was inhibited by WM4. By contrast, WM382 prevented production of both processed forms, leaving unprocessed RAP1 protein (p87). Similar results were obtained for ASP, which is cleaved by PMIX (Pino et al., 2017). While ASP was processed in untreated and E64- and WM4-treated parasites (Figure 6C), cleavage was inhibited by WM382, indicating PMIX processing. These findings confirm that WM4 specifically blocks PMX function, whereas WM382 blocks both PMX and PMIX activity.

To determine whether PMIX was autocatalytically activated, as occurs for rPMX (Figure S4), we analyzed the effect of WM4 and WM382 in *P. falciparum* parasites. Processing of PMIX was not inhibited by either E64 or WM4 (Figure 6D). However, WM382 inhibited processing, confirming that autocatalytic cleavage of PMIX was required for its activation.

RON3, a protein inserted into the PV at merozoite invasion, plays an essential role in the development of the ring stage (Low et al., 2019). E64 and WM4 did not block processing of RON3 (Figure 6E). However, WM382 did and the full-length protein, as well as an additional processed product (p55), were detected. A PMX-consensus cleavage site was identified in RON3, and it was shown to be efficiently cleaved by rPMX (Figure 2D). Therefore, RON3 is processed by PMIX in *P. falciparum* (Low et al., 2019).

MTRAP is expressed in asexual and sexual stages and is essential for gamete egress from erythrocytes for transmission to mosquitoes (Bargieri et al., 2016). E64 and WM4 did not inhibit its processing (Figure 6F); however, WM382 inhibited processing, although not completely, suggesting the involvement of PMIX in processing of MTRAP in blood stages.

PMX Processing Is Autocatalytic and Required for Activation of SUB2

SUB2 is an essential protease responsible for shedding of proteins from invading merozoites, including AMA1 and MSP1 (Hackett et al., 1999). SUB2 processing is mediated by autocatalytic activity; however, a second cleavage event occurs via an unknown protease (Child et al., 2013). Both WM4 and WM382 inhibited this second processing event in SUB2 (Figure 6G), showing this cleavage is mediated by PMX. The full-length protein also accumulates in the presence of inhibitors, suggesting that the autocatalytic processing of SUB2 has been affected. These results suggest that PMX processes SUB2 and this cleavage may be essential for its activity and function.

AMA1 plays an essential role in merozoite invasion by forming the tight junction with RON2 (Cao et al., 2009; Tonkin et al., 2011), and it is processed and shed from the invading merozoite surface by SUB2 (Olivieri et al., 2011). In merozoites, AMA1 was processed (Figure 6H), and these events were not affected by E64. However, both WM4 and WM382 inhibited cleavage. Similar experiments with MSP1, a key protein on the merozoite surface required for egress and invasion (Silmon de Monerri et al., 2011), showed cleavage of the known SUB1 sites are inhibited by both WM4 and WM382 because PMX activates SUB1 (Figure 6I). Additionally, cleavage of MSP1 by SUB2 was also inhibited by both compounds with WM382 having higher activity (Figure 6I). These results show that PMX activates SUB2, which is required for the subsequent processing of AMA1 and MSP1.

PMX in *P. falciparum* parasite extracts is processed from a 66 kDa (p66) polypeptide into two fragments of 51 kDa (p51) and 44 kDa (p44) (Figure 6J). Similar processing was observed for rPMX, where autocatalytic activity cleaved the protein at two positions to release the prodomain (Figure S4). To show that PMX is autocatalytically activated, we tested the ability of WM382 and WM4 to block its processing in the parasite. Both compounds blocked cleavage of PMX demonstrating that it is processed at two sites within the enzyme and that these cleavage events are required for its catalytic activity.

PMX Is Responsible for Proteolytic Processing of the Rh Family and Ripr

Rh proteins are key ligands playing a role in merozoite invasion, and they are proteolytically cleaved by ROM4, resulting in release and shedding from the parasite membrane during invasion (O'Donnell et al., 2006). However, additional processing events occur by an unknown protease(s). WM4 and WM382

(D) Liver infection (52 hpi) was similar in all treatment groups and was reduced at 55 hpi. Mean ± SD.
(E) Percentage loss of bioluminescence (egress) between 52 and 55 h. Mean ± SD.
(F) Whole-body luminescence for blood infection at 65 hpi. Mean ± SD.
(G) Blood parasitemia (FACS) at 65 hpi. Mean ± SD.
(H) Time to patent blood infection for mice infected i.v. with sporozoites.
(I) Time to patent blood infection following injection of 65 hpi supernatants from DMSO and WM382-treated *P. berghei*-infected HepG2 *in vitro* cultures.
(J) Time-to-patent blood infection for mice infected with sporozoites by mosquito bite. Mean ± SD shown. *p < 0.05, **p < 0.01, ***p < 0.001.
Data in H–J represent 5 to 15 mice per group. LOD, limit of detection.

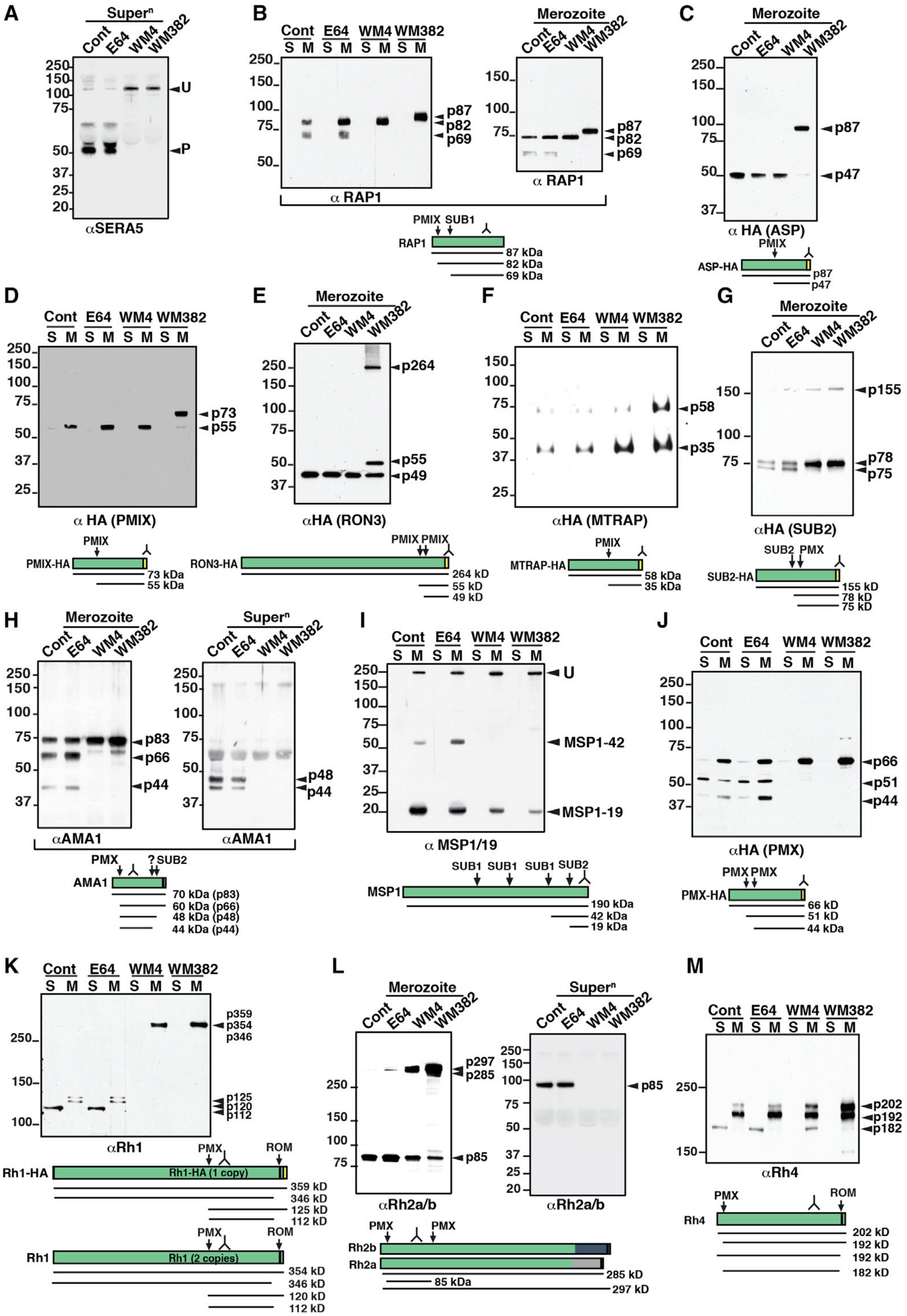

Figure 6. PMIX and PMX Process Invasion Proteins

(A) SERA5 processing inhibited by WM4 and WM382.

(B) RAP1 processed by PMIX and SUB1 (left) and separated further for merozoites (right).

(C) Merozoites probed with αASP inhibited by WM382.

(D) PMIX autocatalytically processed and inhibited by WM382.

(E) RON3 processing inhibited by WM382.

(F) MTRAP processing inhibited by WM382.

(G) SUB2 processing by PMX.

(legend continued on next page)

were used to determine whether processing of Rh proteins was occurring through PMX or PMIX (Figures 6K–6M). In untreated or E64-treated merozoites, anti-PfRh1 antibody detected the processed forms of PfRh1 (Figure 6K). In E64-treated parasites, the processed fragments were released into the supernatant: the PVM remains in place but is porous, so PV proteins escape into the supernatant (Hale et al., 2017) (Figure S7). In contrast, in parasites treated with WM4 or WM382, the large fragment cleaved by ROM4 was not released into the supernatant, suggesting the PVM remained intact with proteins retained within the PV. Affinity purification of Rh1 from parasites and mass spectrometry identified a cleavage site (Figure S7), and a peptide corresponding to the consensus sequence was cleaved by rPMX (Figure 2D), confirming that Rh1 was processed at this site by PMX in the parasite.

Similarly, WM4 and WM382 significantly inhibited processing of other members of the Rh protein family including Rh2a, Rh2b (Figure 6L), and Rh4 (Figure 6M). These results identified two PMX processing sites within Rh2a and Rh2b and one in PfRh4. Purification of Rh2a and Rh2b proteins from *P. falciparum* and mass spectrometry identified the cleavage sites (Figure S7), and peptides corresponding to these sequences were efficiently cleaved by rPMX (Rh2N and Rh2C) (Figure 2D), showing that Rh2a and Rh2b are processed by PMX in *P. falciparum*. A PMX consensus cleavage sequence was also identified at the Rh4 processing site, and a peptide was shown to be efficiently cleaved by rPMX (Figure 2D). These results show that Rh2a, Rh2b, and Rh4 are proteolytically processed by PMX.

Rh5 is an essential member of the Rh family responsible for binding to basigin (CD147) (Crosnier et al., 2011) in the form of a complex with CyRPA and Ripr (Wong et al., 2019). Treatment of *P. falciparum* parasites with WM4 or WM382 blocked processing of Rh5, showing that it was also processed by PMX (Figure 7A). Rh5 was affinity purified from *P. falciparum* parasites, and mass spectrometry identified the site of cleavage and a PMX consensus sequence identified (Figure S7). A Rh5 peptide was cleaved by rPMX (Figure 2D), confirming that PMX proteolytically processes Rh5. We next tested whether Ripr and CyRPA were also processed by PMX and/or PMIX and whether WM4 and WM382 blocked release of the processed forms of Ripr into the supernatant (Figure 7B). Since Ripr is known to be partially processed unlike most other invasins, we conclude that it is likely proteolytically cleaved by PMX but may also be a substrate of PMIX. Ripr was affinity purified from *P. falciparum* parasites and mass spectrometry identified the site of cleavage (Figure S7). A Ripr peptide containing this consensus sequence was cleaved by rPMX (Figure 2D). CyRPA was not proteolytically processed (data not shown). These results demonstrate that PMX and PMIX play an important role in proteolytic processing of proteins of the Rh5 complex.

PMX Is Responsible for Proteolytic Processing of the EBL Family of Invasion Ligands

The EBL proteins play an important and overlapping role with the Rh family in the initial interaction of the merozoite with the erythrocyte membrane during invasion of *P. falciparum* (Koch et al., 2017; Lopaticki et al., 2011; Sisquella et al., 2017; Weiss et al., 2015). None of the EBL family members have been reported to be proteolytically cleaved within their ectodomains, although they are released from the merozoite surface by ROM4 (Baker et al., 2006). To determine whether the EBA proteins were further processed, and whether PMX or PMIX played a role, we tested the effect of WM4 and WM382. Treatment with WM4 and WM382 revealed a processing event of EBA140 inhibited by both compounds and resulted in accumulation of full-length protein (Figure 7C). Similarly, for EBA175 (Figure 7D), WM4 and WM382 inhibited a cleavage event with accumulation of the full-length protein (p175). An antibody to the ectodomain identified the ROM4-cleaved proteins, consistent with a PMX processing site approximately 10 kDa from the signal peptidase cleavage (Figure 7E). In similar experiments, we showed EBA181 was present as a full-length protein in untreated and E64-treated parasites (Figure 7F). When WM4- and WM382-treated parasites were tested, a predominant unprocessed form was evident, consistent with PMX processing at the N terminus. A PMX consensus cleavage sequence was identified for EBA140, EBA175, and EBA181 approximately 10 kDa from the signal sequence, and EBA175 and EBA181 peptides corresponding to this sequence were cleaved by rPMX (Figure 2D). These results show that PMX was responsible for processing of EBA140, EBA175, and EBA181 at the N terminus of the protein ectodomain at the boundary of the F1 region that defines the receptor binding domains.

DISCUSSION

A phenotypic high throughput screen of a library of small molecules that inhibit aspartic proteases, performed against asexual blood stages of *P. falciparum,* identified a novel class of antimalarial compounds. During optimization studies, WM382, an orally available lead compound that has potent activity *in vitro* against *P. falciparum* and *P. knowlesi* and can cure *P. berghei* and *P. falciparum* infections in mice, has been identified. This compound has dual activity against both PMIX and PMX and is active against blood, liver, and mosquito stages of the life cycle. Because of the novel mechanism of action, there was no cross-resistance against these compounds in *P. falciparum* parasite lines resistant to current antimalarials. Additionally, its activity against two essential targets creates a high threshold against the development of resistance. Therefore, efforts to identify an efficacious and safe antimalarial drug from this series of compounds are currently ongoing.

(H) αAMA1 detection after treatment with E64, WM4, and WM382. Left: merozoites. Right: supernatant.
(I) MSP1 detection (αMSP1/19) from merozoites treated with E64, WM4, and WM382.
(J) PMX autocatalytically processed and inhibited by WM4 and WM382.
(K) Rh1 processed by PMX. W2mef-HA parasites have three *Rh1* genes with one HA tagged.
(L). Rh2a/b are processed by PMX. Left: merozoites. Right: supernatants. Rh2a and Rh2b are identical but differ at C terminus (blue and gray domains).
(M) Rh4 (αRh4) processed by PMX.
A summary schematic for each protein is shown (HA-tag, yellow box; transmembrane, black box. Supernatants (Super^{n} or S) released from merozoites. M, merozoites. Position of detecting antibody signified by ⅄. Molecular weight kDa.

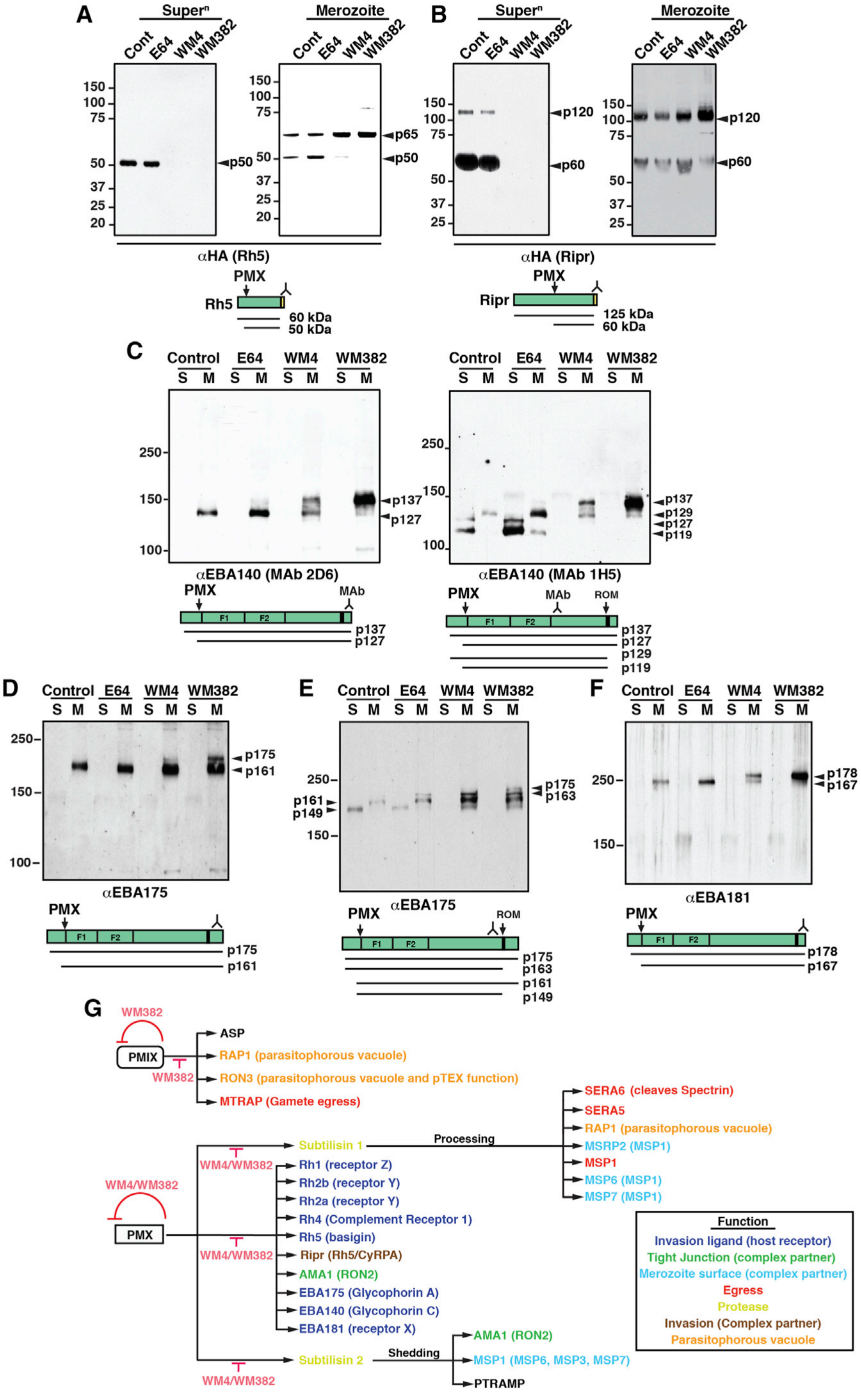

Figure 7. Rh5 Complex and EBA Proteins Processed by PMX or PMIX

(A) Rh5 processed by PMX and inhibited by WM4 and WM382. Left: supernatant. Right: merozoites.
(B) Ripr processed by PMX or PMIX inhibited by WM4 and WM382. Left: supernatant. Right: merozoites.
(C) EBA140 processed by PMX and inhibited by WM4 and WM382. Left: αEBA140 monoclonal antibody (mAb) (2D6). Right: αEBA140 mAb (1H5).
(D) EBA175 processed by PMX and inhibited by WM4 and WM382.
(E) EBA175 processed by PMX and inhibited by WM4 and WM382.
(F) EBA181 processed by PMX and inhibited by WM4 and WM382.
(G) Summary of PMX function.
A summary schematic for each protein is shown (HA-tag, yellow box; transmembrane, black box). Supernatants (Super^n or S) released from merozoites. M, merozoites. Position of detecting antibody signified by ⅄. C–F schematics show positions of F1 and F2 receptor binding domains. Molecular weight kDa.

Discovery of compounds that specifically block PMX function, as well as those that are dual inhibitors of PMIX and X, has provided tools to interrogate function of these proteases in *Plasmodium* biology. The dual activity of WM382 against PMIX and PMX was demonstrated using a number of functional criteria. First, inducible knockdown lines of PMIX or X were both more sensitive than parental 3D7 to WM382. Second, WM382 binding increased the thermal stability (Martinez Molina and Nordlund, 2016) of both PMIX and X, indicating direct engagement of the compound with these proteases. Third, WM382 inhibited processing of known PMIX substrates (Pino et al., 2017), as well as processing of PMX substrates. In contrast, WM4 and WM5 are specific inhibitors of PMX, as functional knockdown rendered parasites more sensitive to WM4 and WM5, whereas PMIX functional knockdown had no effect. Also, WM4 binding increased the thermal stability of PMX but not PMIX. Lastly, WM4 does not inhibit processing of PMIX substrates, whereas it blocks processing of proteins processed by PMX (Pino et al., 2017).

Interestingly, rPMX protease cleaves both PMIX and PMX peptide substrates efficiently, and mutational analysis has shown the cleavage consensus sequence to be similar for both enzymes. These results show that the selectivity observed for PMIX and PMX activity against different protein substrates in parasites is not because of substrate sequence motifs per se, but other substrate-specific factors, such as their subcellular localization. PMIX localizes to the rhoptries while PMX is present in the exonemes suggesting that substrates for each protease would be in these subcellular localizations (Nasamu et al., 2017). However, this does not conform with the known subcellular localizations of the protein substrates of PMIX and PMX that we have identified, including the PfRh and EBL invasion ligands which are localized in the rhoptries and micronemes respectively (Healer et al., 2002). This suggests that that exposure of different proteins to PMX and PMIX before and during merozoite invasion is complex, involving a cascade of events that release essential proteins at distinct times.

Although inhibition of PMX activity is evident initially as an erythrocyte egress phenotype, this protease plays a key role in processing members of the EBL and Rh protein families required for invasion of erythrocytes. EBL proteins are processed by PMX at the boundary of their N terminus and the F1-F2 receptor binding domain. At this stage, it is not known how these PMX-mediated processing events affect the function of these proteins; however, it may play an important role in maturation of the proteins for display on the merozoite surface as well as their ability to interact with their cognate receptors. The individual Rh proteins are processed by PMX at different positions along their length, and this is likely critical for their function. Rh5 is a leading malaria vaccine candidate and plays an essential role, in complex with CyRPA and Ripr (Volz et al., 2016; Wong et al., 2019) and in binding to the erythrocyte surface protein basigin (Crosnier et al., 2011). Processing by PMX results in cleavage of the N-terminal 15 kDa domain of Rh5 (Figure 7G). It has been reported that this N-terminal region binds the GPI-anchored protein Pf113, tethering the Rh5/CyRPA/Ripr complex to the parasite plasma membrane, which may be critical for its function in the merozoite-erythrocyte interaction and invasion (Galaway et al., 2017). Cleavage of Rh5 by PMX at the N terminus would release the Rh5/CyRPA/Ripr complex from Pf113 at the merozoite surface. Insertion of processed Rh5/CyRPA/Ripr complex into the erythrocyte membrane occurs during invasion (Wong et al., 2019), and PMX-mediated processing of Rh5 may be crucial for this step.

Release of the EBL and Rh protein families, as well as the Rh5/CyRPA/Ripr complex, into the supernatant during merozoite invasion was blocked by WM4 and WM382, whereas E64 treatment had no effect on this process. E64 is a protease inhibitor that also blocks merozoite egress from the erythrocyte via inhibition of SERA6, a process occurring downstream of PMX (Hale et al., 2017). Treatment of *Plasmodium*-infected cells with E64 results in a leaky PVM, allowing release of SUB2 and rhomboid protease-cleaved proteins from the merozoite into the supernatant. In contrast, treatment of *P. falciparum*-infected cells with WM4 and WM382 prevents PVM breakdown, and, while rhomboid proteases cleave Rh, EBL, and other proteins and releases them from the merozoite surface, they remain trapped in the PV. The role of PMX in rupture of the PVM is unknown at this point, however, our results suggest this process is upstream of the proteolytic events blocked by E64 and likely to be through its role in activating SUB1 (Pino et al., 2017; Thomas et al., 2018).

Blocking PMX function by WM382 has identified new protein substrates of this protease. Previously, it has been shown that PMX processes SUB1, which is required for maturation of AMA1, MSP1, and SERA5 (Pino et al., 2017). These results have been confirmed and, furthermore, we have shown that PMX processes and activates SUB2, whose inhibition then blocks shedding of AMA1, MSP1, and PTRAMP during invasion (Olivieri et al., 2011). Additionally, we have shown that PMX directly processes AMA1 at a previously unidentified site and, further, both PMX and PMIX are responsible for their own maturation and activation, in contrast to a previous study with inhibitors of PMX and IX (Nasamu et al., 2017).

While PMIX and PMX are responsible for the processing of key proteins involved in egress and invasion, they also process proteins important for ring-stage development after invasion, including RAP1 (Baldi et al., 2000) and RON3 (Low et al., 2019). RAP1 is released into the PV during invasion and, together with its complex partners RAP2 or RAP3, may play a role in establishing this compartment. Similarly, RON3 is deposited in the PV; it is essential for development beyond the ring stage, functions with the PTEX translocon in the export of parasite proteins to the erythrocyte cytosol, and is required for transport of glucose from the host cell into the PV (Low et al., 2019). While RAP1 and RON3 have been identified as substrates of PMIX that function in ring-stage development, there are likely to be others not yet identified. Together, PMIX and PMX have a broad, both direct and indirect, function in the activation of many proteins involved in egress and invasion as well as early parasite development, emphasizing their importance as drug targets (Nasamu et al., 2017; Pino et al., 2017).

In *P. berghei*, PMX is expressed in mature gametocytes (Pino et al., 2017), and the inhibitor 49c blocked gamete maturation from gametocytes, providing evidence that PMX was required for transmission. In the present study, we examined the effect of the PMX-specific inhibitor WM4 on *P. falciparum* transmission and found that there was no effect on the development of gametocytes or on *P. falciparum* infection of mosquitoes. In contrast, WM382, which inhibits both PMIX and PMX, potently blocked transmission. These results indicate that in *P. falciparum,* PMIX function is important for oocyst development in mosquitoes. We cannot rule out the possibility that WM382 is active against other plasmepsins such as PMVI, which has been shown to be essential for *P. berghei* transmission to mosquitoes (Ecker et al., 2008).

Interestingly, we have shown that processing of MTRAP, a protein essential for gamete egress from erythrocytes and transmission to mosquitoes (Bargieri et al., 2016), does not appear to be dependent on PMX, suggesting that PMIX is involved. Previously, it has been shown PMIX was not expressed in *P. berghei* gametocytes (Pino et al., 2017), but its translation may be initiated at gamete formation and important for processing and function of proteins such as MTRAP for gamete release (Lasonder et al., 2012). This would be consistent with our results showing that WM382 and not WM4 was able to block MTRAP processing and transmission of *P. falciparum* to mosquitoes, suggesting that PMIX plays a key role in processing essential proteins for this step.

A single dose of WM382 following sporozoite infection of the liver prevented mice from developing patent blood infection. This protection was superior to a previous study which examined the same *P. berghei* mCherry-luciferase parasite line also in BALB/c mice treated with the PMX/IX inhibitor 49c (Pino et al., 2017). In their study, although 49c blocked merosome release *in vitro* all 49c-treated mice went on to develop blood infection, clearly showing an incomplete *in vivo* block. Our study supports a further role for PMIX/PMX in the maturation of exoerythrocytic merozoites for the first erythrocyte infection cycle. This is because WM382-treated mice had an undetectable first wave of erythrocyte infection and because WM382 prevented infection of mice by *in vitro* HepG2-derived merosomes. This confirms that PMX, and likely PMIX, are critical in the maturation of liver merozoites beyond enabling merosome formation or egress from hepatocytes, highlighting commonality in the developmental pathways of both liver and blood merozoites for

erythrocyte infection. It is possible that 49c may also inhibit another protease required for merosome release that are not targeted by WM382 (Pino et al., 2017).

While it has been possible to select drug resistance in *P. falciparum* to both WM4 and WM5, this was done by slow, incremental increases in drug concentration over a 6-month period *in vitro*. Similar experiments have not, as of yet, selected resistance to WM382, most likely because this would require amplification of (or mutation in) both *PMIX* and *PMX* genes, which are on different chromosomes. Treatment of large numbers of *P. falciparum* parasites with drug, in time to resistance studies (Ding et al., 2012) have failed to select resistance to either WM4 or WM382, whereas resistant parasites were obtained for atovaquone, which requires a single point mutation in the cytochrome b1 gene (Korsinczky et al., 2000). While we cannot conclude from these experiments that it is impossible to generate resistance to WM382, it indicates that the survival afforded via *PMX* amplifications do not confer protection against a dual inhibitor that targets both PMIX and PMX. This demonstrates a high barrier for the development of resistance against this compound. A high resistance threshold is a valuable feature for an antimalarial drug, and these findings prioritize this compound class for progression to clinical trials.

STAR★METHODS

Detailed methods are provided in the online version of this paper and include the following:

- KEY RESOURCES TABLE
- LEAD CONTACT AND MATERIALS AVAILABILITY
- EXPERIMENTAL MODELS AND SUBJECT DETAILS
  - Ethics Statement
  - *Plasmodium falciparum* Culture
  - *Plasmodium knowlesi* Culture
  - *Plasmodium berghei* Maintenance
  - *P. falciparum* Gametocyte Culture
  - Mouse Models
- METHOD DETAILS
  - Construction of *P. falciparum* Lines Expressing HA-Tagged Proteins
  - High Throughput Screening and EC_{50} Determination of *P. falciparum* Growth
  - Determination of EC_{50} in Knock down Parasite Lines
  - *P. knowlesi* Drug Sensitivity Assay
  - *In Vitro* Selection of Parasites Resistant to WM4 and WM5
  - Genome Sequencing and Bioinformatics Analysis
  - Southern Blot Analysis
  - PMX Pulldown with WM856
  - PMX Expression and Purification
  - Identification of Potential PMX and PMIX Substrates
  - FRET Based Assay for PMX Cleavage of Peptide Substrates
  - CETSA Thermal Stability Assays
  - Time of Drug Killing
  - Invasion Assay
  - Ring Stage Survival Assay for Artesunate
  - Determination of Time to Resistance
  - *P. falciparum* Competition Growth Assays
  - Peters' 4-Day Suppressive Test for *P. berghei* Infection in Mice
  - Dose Ranging Test for *P. berghei* Infection in Mice
  - *P. falciparum* Humanized NOD-*scid* IL2R_null Mouse Model
  - Bioanalytical Determination of W382 in Mouse Blood Samples by LC-MS/MS
  - *In Vivo* Analysis of *P. berghei* Liver Parasite Growth and Transition to Blood Infection
  - *In Vitro* Analysis of *P. berghei* Liver Parasite Detachment and Merosome Transfer to Mice
  - Immunofluorescent Assay for *P. berghei* Liver Parasites
  - Peptide Cleavage Assays
  - Rationale for Identification of Potential PMX and PMIX Protein Substrates
  - Processing Inhibition Assays
  - *P. falciparum* Schizont Egress Inhibition Assay
- QUANTIFICATION AND STATISTICAL ANALYSIS
- DATA AND CODE AVAILABILITY

SUPPLEMENTAL INFORMATION

Supplemental Information can be found online at https://doi.org/10.1016/j.chom.2020.02.005.

ACKNOWLEDGMENTS

We thank E. Loza, L. Spencer, J. Curtis, and A. Schäfer for assistance with mouse experiments; the Victorian Red Cross Blood Bank for blood; and L. Dagley, T. Nebl, and A. Webb for mass spectroscopy. We acknowledge A. Stamford, B. Mckittrick, and J. Cumming for support of initial screening efforts. This work was possible through support of The Wellcome Trust [109662/Z/15/Z and 202749/Z/16/Z]; Victorian State Government Operational Infrastructure Support and Australian Government NHMRC IRIISS; National Health and Medical Research Council of Australia.

AUTHOR CONTRIBUTIONS

P.F. and R.W.J.S. designed and performed *in vivo* PD studies; L.Z., J.A.M., M.R., and B.E.S. designed WM382; A.F.C., A.N., C.B., D.B.O., H.J.S., J.A.B., J.A.M., J.A.R., J.C., J.H., J.K.T., J.S.P., K.J., K.-Y.G., K.R., L.R., M.H., M.H.D., M.R., M.V., N.M., P.F., R.W.J.S., S.L., T.K., T.P., T.T., W.-H.T., and Z.G. designed, interpreted, and/or performed assays or experiments; A.F.C., D.B.O., J.H., P.F., and R.W.J.S. drafted manuscript; A.F.C., D.B.O., M.R., and P.F. were Team Leads. All authors read and edited the manuscript.

DECLARATION OF INTERESTS

A.F.C., M.R., P.F., Z.G., Z.L., J.M., D.B.O., B.E.S., J.K.T, T.T., and L.Z. have a patent Antimalarial Agents PCT/CN2019/100781 based on the compounds in this manuscript.

Received: October 25, 2019
Revised: December 22, 2019
Accepted: February 11, 2020
Published: February 23, 2020

REFERENCES

Alaganan, A., Singh, P., and Chitnis, C.E. (2017). Molecular mechanisms that mediate invasion and egress of malaria parasites from red blood cells. Curr. Opin. Hematol. *24*, 208–214.

Angulo-Barturen, I., Jiménez-Díaz, M.B., Mulet, T., Rullas, J., Herreros, E., Ferrer, S., Jiménez, E., Mendoza, A., Regadera, J., Rosenthal, P.J., et al. (2008). A murine model of *falciparum*-malaria by *in vivo* selection of competent strains in non-myelodepleted mice engrafted with human erythrocytes. PLoS One *3*, e2252.

Bacon, D.J., Latour, C., Lucas, C., Colina, O., Ringwald, P., and Picot, S. (2007). Comparison of a SYBR green I-based assay with a histidine-rich protein II enzyme-linked immunosorbent assay for in vitro antimalarial drug efficacy testing and application to clinical isolates. Antimicrob. Agents Chemother. *51*, 1172–1178.

Bahl, A., Brunk, B., Crabtree, J., Fraunholz, M.J., Gajria, B., Grant, G.R., Ginsburg, H., Gupta, D., Kissinger, J.C., Labo, P., et al. (2003). PlasmoDB: the Plasmodium genome resource. A database integrating experimental and computational data. Nucleic Acids Res. *31*, 212–215.

Baker, R.P., Wijetilaka, R., and Urban, S. (2006). Two *Plasmodium* rhomboid proteases preferentially cleave different adhesins implicated in all invasive stages of malaria. PLoS Pathog. *2*, e113.

Baldi, D.L., Andrews, K.T., Waller, R.F., Roos, D.S., Howard, R.F., Crabb, B.S., and Cowman, A.F. (2000). RAP1 controls rhoptry targeting of RAP2 in the malaria parasite *Plasmodium falciparum*. EMBO J. *19*, 2435–2443.

Bargieri, D.Y., Thiberge, S., Tay, C.L., Carey, A.F., Rantz, A., Hischen, F., Lorthiois, A., Straschil, U., Singh, P., Singh, S., et al. (2016). *Plasmodium* Merozoite TRAP Family Protein Is Essential for Vacuole Membrane Disruption and Gamete Egress from Erythrocytes. Cell Host Microbe *20*, 618–630.

Battle, K.E., Lucas, T.C.D., Nguyen, M., Howes, R.E., Nandi, A.K., Twohig, K.A., Pfeffer, D.A., Cameron, E., Rao, P.C., Casey, D., et al. (2019). Mapping the global endemicity and clinical burden of Plasmodium vivax, 2000-17: a spatial and temporal modelling study. Lancet *394*, 332–343.

Boyle, M.J., Richards, J.S., Gilson, P.R., Chai, W., and Beeson, J.G. (2010). Interactions with heparin-like molecules during erythrocyte invasion by *Plasmodium falciparum* merozoites. Blood *115*, 4559–4568.

Cameron, D.L., Schröder, J., Penington, J.S., Do, H., Molania, R., Dobrovic, A., Speed, T.P., and Papenfuss, A.T. (2017). GRIDSS: sensitive and specific genomic rearrangement detection using positional de Bruijn graph assembly. Genome Res. *27*, 2050–2060.

Cao, J., Kaneko, O., Thongkukiatkul, A., Tachibana, M., Otsuki, H., Gao, Q., Tsuboi, T., and Torii, M. (2009). Rhoptry neck protein RON2 forms a complex with microneme protein AMA1 in *Plasmodium falciparum* merozoites. Parasitol. Int. *58*, 29–35.

Child, M.A., Harris, P.K., Collins, C.R., Withers-Martinez, C., Yeoh, S., and Blackman, M.J. (2013). Molecular determinants for subcellular trafficking of the malarial sheddase PfSUB2. Traffic *14*, 1053–1064.

Coley, A.M., Campanale, N.V., Casey, J.L., Hodder, A.N., Crewther, P.E., Anders, R.F., Tilley, L.M., and Foley, M. (2001). Rapid and precise epitope mapping of monoclonal antibodies against Plasmodium falciparum AMA1 by combined phage display of fragments and random peptides. Protein Eng. *14*, 691–698.

Collins, C.R., Hackett, F., Atid, J., Tan, M.S.Y., and Blackman, M.J. (2017). The *Plasmodium falciparum* pseudoprotease SERA5 regulates the kinetics and efficiency of malaria parasite egress from host erythrocytes. PLoS Pathog. *13*, e1006453.

Crosnier, C., Bustamante, L.Y., Bartholdson, S.J., Bei, A.K., Theron, M., Uchikawa, M., Mboup, S., Ndir, O., Kwiatkowski, D.P., Duraisingh, M.T., et al. (2011). Basigin is a receptor essential for erythrocyte invasion by *Plasmodium falciparum*. Nature *480*, 534–537.

Ding, X.C., Ubben, D., and Wells, T.N. (2012). A framework for assessing the risk of resistance for anti-malarials in development. Malar. J. *11*, 292.

Dziekan, J.M., Yu, H., Chen, D., Dai, L., Wirjanata, G., Larsson, A., Prabhu, N., Sobota, R.M., Bozdech, Z., and Nordlund, P. (2019). Identifying purine nucleoside phosphorylase as the target of quinine using cellular thermal shift assay. Sci. Transl. Med. *11*, eaau3174.

Ecker, A., Bushell, E.S., Tewari, R., and Sinden, R.E. (2008). Reverse genetics screen identifies six proteins important for malaria development in the mosquito. Mol. Microbiol. *70*, 209–220.

Fairlie, W.D., Spurck, T.P., McCoubrie, J.E., Gilson, P.R., Miller, S.K., McFadden, G.I., Malby, R., Crabb, B.S., and Hodder, A.N. (2008). Inhibition of malaria parasite development by a cyclic peptide that targets the vital parasite protein SERA5. Infect. Immun. *76*, 4332–4344.

Ferreri, A.J., Illerhaus, G., Zucca, E., and Cavalli, F.; International Extranodal Lymphoma Study Group (2010). Flows and flaws in primary central nervous system lymphoma. Nat. Rev Clin Oncol. *7*, https://doi.org/10.1038/nrclinonc.2010.9-c1.

Franke-Fayard, B., Trueman, H., Ramesar, J., Mendoza, J., van der Keur, M., van der Linden, R., Sinden, R.E., Waters, A.P., and Janse, C.J. (2004). A *Plasmodium berghei* reference line that constitutively expresses GFP at a high level throughout the complete life cycle. Mol. Biochem. Parasitol. *137*, 23–33.

Galaway, F., Drought, L.G., Fala, M., Cross, N., Kemp, A.C., Rayner, J.C., and Wright, G.J. (2017). P113 is a merozoite surface protein that binds the N terminus of *Plasmodium falciparum* RH5. Nat. Commun. *8*, 14333.

Gamo, F.J., Sanz, L.M., Vidal, J., de Cozar, C., Alvarez, E., Lavandera, J.L., Vanderwall, D.E., Green, D.V., Kumar, V., Hasan, S., et al. (2010). Thousands of chemical starting points for antimalarial lead identification. Nature *465*, 305–310.

Ghorbal, M., Gorman, M., Macpherson, C.R., Martins, R.M., Scherf, A., and Lopez-Rubio, J.J. (2014). Genome editing in the human malaria parasite *Plasmodium falciparum* using the CRISPR-Cas9 system. Nat. Biotechnol. *32*, 819–821.

Gilberger, T.W., Thompson, J.K., Reed, M.B., Good, R.T., and Cowman, A.F. (2003a). The cytoplasmic domain of the Plasmodium falciparum ligand EBA-175 is essential for invasion but not protein trafficking. J. Cell Biol. *162*, 317–327.

Gilberger, T.W., Thompson, J.K., Triglia, T., Good, R.T., Duraisingh, M.T., and Cowman, A.F. (2003b). A novel erythrocyte binding antigen-175 paralogue from Plasmodium falciparum defines a new trypsin-resistant receptor on human erythrocytes. J. Biol. Chem. *278*, 14480–14486.

Graewe, S., Rankin, K.E., Lehmann, C., Deschermeier, C., Hecht, L., Froehlke, U., Stanway, R.R., and Heussler, V. (2011). Hostile takeover by *Plasmodium*: reorganization of parasite and host cell membranes during liver stage egress. PLoS Pathog. *7*, e1002224.

Hackett, F., Sajid, M., Withers-Martinez, C., Grainger, M., and Blackman, M.J. (1999). PfSUB-2: a second subtilisin-like protein in *Plasmodium falciparum* merozoites. Mol. Biochem. Parasitol. *103*, 183–195.

Hale, V.L., Watermeyer, J.M., Hackett, F., Vizcay-Barrena, G., van Ooij, C., Thomas, J.A., Spink, M.C., Harkiolaki, M., Duke, E., Fleck, R.A., et al. (2017). Parasitophorous vacuole poration precedes its rupture and rapid host erythrocyte cytoskeleton collapse in *Plasmodium falciparum* egress. Proc. Natl. Acad. Sci. USA *114*, 3439–3444.

Healer, J., Crawford, S., Ralph, S., McFadden, G., and Cowman, A.F. (2002). Independent translocation of two micronemal proteins in developing *Plasmodium falciparum* merozoites. Infect. Immun. *70*, 5751–5758.

Hodder, A.N., Sleebs, B.E., Czabotar, P.E., Gazdik, M., Xu, Y., O'Neill, M.T., Lopaticki, S., Nebl, T., Triglia, T., Smith, B.J., et al. (2015). Structural basis for plasmepsin V inhibition that blocks export of malaria proteins to human erythrocytes. Nat. Struct. Mol. Biol. *22*, 590–596.

Jiménez-Díaz, M.B., Mulet, T., Viera, S., Gómez, V., Garuti, H., Ibáñez, J., Alvarez-Doval, A., Shultz, L.D., Martínez, A., Gargallo-Viola, D., and Angulo-Barturen, I. (2009). Improved murine model of malaria using Plasmodium falciparum competent strains and non-myelodepleted NOD-scid IL2Rgammanull mice engrafted with human erythrocytes. Antimicrob. Agents Chemother. *53*, 4533–4536.

Kite, W.A., Melendez-Muniz, V.A., Moraes Barros, R.R., Wellems, T.E., and Sá, J.M. (2016). Alternative methods for the *Plasmodium falciparum* artemisinin ring-stage survival assay with increased simplicity and parasite stage-specificity. Malar. J. *15*, 94.

Koboldt, D.C., Zhang, Q., Larson, D.E., Shen, D., McLellan, M.D., Lin, L., Miller, C.A., Mardis, E.R., Ding, L., and Wilson, R.K. (2012). VarScan 2: somatic mutation and copy number alteration discovery in cancer by exome sequencing. Genome Res. *22*, 568–576.

Koch, M., Wright, K.E., Otto, O., Herbig, M., Salinas, N.D., Tolia, N.H., Satchwell, T.J., Guck, J., Brooks, N.J., and Baum, J. (2017). *Plasmodium falciparum* erythrocyte-binding antigen 175 triggers a biophysical change in the red blood cell that facilitates invasion. Proc. Natl. Acad. Sci. USA *114*, 4225–4230.

Korsinczky, M., Chen, N., Kotecka, B., Saul, A., Rieckmann, K., and Cheng, Q. (2000). Mutations in *Plasmodium falciparum* cytochrome b that are associated with atovaquone resistance are located at a putative drug-binding site. Antimicrob. Agents Chemother. *44*, 2100–2108.

Langmead, B., and Salzberg, S.L. (2012). Fast gapped-read alignment with Bowtie 2. Nat. Methods *9*, 357–359.

Lasonder, E., Green, J.L., Camarda, G., Talabani, H., Holder, A.A., Langsley, G., and Alano, P. (2012). The *Plasmodium falciparum* schizont phosphoproteome reveals extensive phosphatidylinositol and cAMP-protein kinase A signaling. J. Proteome Res. *11*, 5323–5337.

Lopaticki, S., Maier, A.G., Thompson, J., Wilson, D.W., Tham, W.H., Triglia, T., Gout, A., Speed, T.P., Beeson, J.G., Healer, J., and Cowman, A.F. (2011). Reticulocyte and erythrocyte binding-like proteins function cooperatively in invasion of human erythrocytes by malaria parasites. Infect. Immun. *79*, 1107–1117.

Low, L.M., Azasi, Y., Sherling, E.S., Garten, M., Zimmerberg, J., Tsuboi, T., Brzostowski, J., Mu, J., Blackman, M.J., and Miller, L.H. (2019). Deletion of *Plasmodium falciparum* Protein RON3 Affects the Functional Translocation of Exported Proteins and Glucose Uptake. MBio *10*, e01460-19.

Makler, M.T., Ries, J.M., Williams, J.A., Bancroft, J.E., Piper, R.C., Gibbins, B.L., and Hinrichs, D.J. (1993). Parasite lactate dehydrogenase as an assay for *Plasmodium falciparum* drug sensitivity. Am. J. Trop. Med. Hyg. *48*, 739–741.

Martinez Molina, D., and Nordlund, P. (2016). The Cellular Thermal Shift Assay: A Novel Biophysical Assay for In Situ Drug Target Engagement and Mechanistic Biomarker Studies. Annu. Rev. Pharmacol. Toxicol. *56*, 141–161.

Menard, D., and Dondorp, A. (2017). Antimalarial Drug Resistance: A Threat to Malaria Elimination. Cold Spring Harb. Perspect. Med. *7*, a025619.

Moon, R.W., Hall, J., Rangkuti, F., Ho, Y.S., Almond, N., Mitchell, G.H., Pain, A., Holder, A.A., and Blackman, M.J. (2013). Adaptation of the genetically tractable malaria pathogen *Plasmodium knowlesi* to continuous culture in human erythrocytes. Proc. Natl. Acad. Sci. USA *110*, 531–536.

Nasamu, A.S., Glushakova, S., Russo, I., Vaupel, B., Oksman, A., Kim, A.S., Fremont, D.H., Tolia, N., Beck, J.R., Meyers, M.J., et al. (2017). Plasmepsins IX and X are essential and druggable mediators of malaria parasite egress and invasion. Science *358*, 518–522.

Nasirian, H., and Ladonni, H. (2006). Artificial bloodfeeding of *Anopheles stephensi* on a membrane apparatus with human whole blood. J. Am. Mosq. Control Assoc. *22*, 54–56.

Nguyen, W., Hodder, A.N., de Lezongard, R.B., Czabotar, P.E., Jarman, K.E., O'Neill, M.T., Thompson, J.K., Jousset Sabroux, H., Cowman, A.F., Boddey, J.A., and Sleebs, B.E. (2018). Enhanced antimalarial activity of plasmepsin V inhibitors by modification of the P_2 position of PEXEL peptidomimetics. Eur. J. Med. Chem. *154*, 182–198.

O'Donnell, R.A., Hackett, F., Howell, S.A., Treeck, M., Struck, N., Krnajski, Z., Withers-Martinez, C., Gilberger, T.W., and Blackman, M.J. (2006). Intramembrane proteolysis mediates shedding of a key adhesin during erythrocyte invasion by the malaria parasite. J. Cell Biol. *174*, 1023–1033.

Oduola, A.M., Omitowoju, G.O., Sowunmi, A., Makler, M.T., Falade, C.O., Kyle, D.E., Fehintola, F.A., Ogundahunsi, O.A., Piper, R.C., Schuster, B.G., and Milhous, W.K. (1997). *Plasmodium falciparum*: evaluation of lactate dehydrogenase in monitoring therapeutic responses to standard antimalarial drugs in Nigeria. Exp. Parasitol. *87*, 283–289.

Olivieri, A., Collins, C.R., Hackett, F., Withers-Martinez, C., Marshall, J., Flynn, H.R., Skehel, J.M., and Blackman, M.J. (2011). Juxtamembrane shedding of *Plasmodium falciparum* AMA1 is sequence independent and essential, and helps evade invasion-inhibitory antibodies. PLoS Pathog. *7*, e1002448.

Pino, P., Caldelari, R., Mukherjee, B., Vahokoski, J., Klages, N., Maco, B., Collins, C.R., Blackman, M.J., Kursula, I., Heussler, V., et al. (2017). A multistage antimalarial targets the plasmepsins IX and X essential for invasion and egress. Science *358*, 522–528.

Prado, M., Eickel, N., De Niz, M., Heitmann, A., Agop-Nersesian, C., Wacker, R., Schmuckli-Maurer, J., Caldelari, R., Janse, C.J., Khan, S.M., et al. (2015). Long-term live imaging reveals cytosolic immune responses of host hepatocytes against *Plasmodium* infection and parasite escape mechanisms. Autophagy *11*, 1561–1579.

Prommana, P., Uthaipibull, C., Wongsombat, C., Kamchonwongpaisan, S., Yuthavong, Y., Knuepfer, E., Holder, A.A., and Shaw, P.J. (2013). Inducible knockdown of *Plasmodium* gene expression using the glmS ribozyme. PLoS One *8*, e73783.

Reed, M.B., Caruana, S.R., Batchelor, A.H., Thompson, J.K., Crabb, B.S., and Cowman, A.F. (2000). Targeted disruption of an erythrocyte binding antigen in Plasmodium falciparum is associated with a switch toward a sialic acid-independent pathway of invasion. Proc. Natl. Acad. Sci. USA *97*, 7509–7514.

Riglar, D.T., Richard, D., Wilson, D.W., Boyle, M.J., Dekiwadia, C., Turnbull, L., Angrisano, F., Marapana, D.S., Rogers, K.L., Whitchurch, C.B., et al. (2011). Super-resolution dissection of coordinated events during malaria parasite invasion of the human erythrocyte. Cell Host Microbe *9*, 9–20.

Roth, A., Adapa, S.R., Zhang, M., Liao, X., Saxena, V., Goffe, R., Li, S., Ubalee, R., Saggu, G.S., Pala, Z.R., et al. (2018). Unraveling the *Plasmodium vivax* sporozoite transcriptional journey from mosquito vector to human host. Sci. Rep. *8*, 12183.

Salmon, B.L., Oksman, A., and Goldberg, D.E. (2001). Malaria parasite exit from the host erythrocyte: a two-step process requiring extraerythrocytic proteolysis. Proc. Natl. Acad. Sci. USA *98*, 271–276.

Scheinin, I., Sie, D., Bengtsson, H., van de Wiel, M.A., Olshen, A.B., van Thuijl, H.F., van Essen, H.F., Eijk, P.P., Rustenburg, F., Meijer, G.A., et al. (2014). DNA copy number analysis of fresh and formalin-fixed specimens by shallow whole-genome sequencing with identification and exclusion of problematic regions in the genome assembly. Genome Res. *24*, 2022–2032.

Schofield, L., Bushell, G.R., Cooper, J.A., Saul, A.J., Upcroft, J.A., and Kidson, C. (1986). A rhoptry antigen of Plasmodium falciparum contains conserved and variable epitopes recognized by inhibitory monoclonal antibodies. Mol. Biochem. Parasitol. *18*, 183–195.

Silmon de Monerri, N.C., Flynn, H.R., Campos, M.G., Hackett, F., Koussis, K., Withers-Martinez, C., Skehel, J.M., and Blackman, M.J. (2011). Global identification of multiple substrates for *Plasmodium falciparum* SUB1, an essential malarial processing protease. Infect. Immun. *79*, 1086–1097.

Singh, B., Kim Sung, L., Matusop, A., Radhakrishnan, A., Shamsul, S.S., Cox-Singh, J., Thomas, A., and Conway, D.J. (2004). A large focus of naturally acquired *Plasmodium knowlesi* infections in human beings. Lancet *363*, 1017–1024.

Singh, S., Alam, M.M., Pal-Bhowmick, I., Brzostowski, J.A., and Chitnis, C.E. (2010). Distinct external signals trigger sequential release of apical organelles during erythrocyte invasion by malaria parasites. PLoS Pathog. *6*, e1000746.

Sisquella, X., Nebl, T., Thompson, J.K., Whitehead, L., Malpede, B.M., Salinas, N.D., Rogers, K., Tolia, N.H., Fleig, A., O'Neill, J., et al. (2017). *Plasmodium falciparum* ligand binding to erythrocytes induce alterations in deformability essential for invasion. eLife *6*, e21083.

Sturm, A., Amino, R., van de Sand, C., Regen, T., Retzlaff, S., Rennenberg, A., Krueger, A., Pollok, J.M., Menard, R., and Heussler, V.T. (2006). Manipulation of host hepatocytes by the malaria parasite for delivery into liver sinusoids. Science *313*, 1287–1290.

Tham, W.H., Wilson, D.W., Lopaticki, S., Schmidt, C.Q., Tetteh-Quarcoo, P.B., Barlow, P.N., Richard, D., Corbin, J.E., Beeson, J.G., and Cowman, A.F. (2010). Complement receptor 1 is the host erythrocyte receptor for Plasmodium falciparum PfRh4 invasion ligand. Proc. Natl. Acad. Sci. USA *107*, 17327–17332.

Tham, W.H., Lim, N.T., Weiss, G.E., Lopaticki, S., Ansell, B.R., Bird, M., Lucet, I., Dorin-Semblat, D., Doerig, C., Gilson, P.R., et al. (2015). *Plasmodium falciparum* Adhesins Play an Essential Role in Signalling and Activation of Invasion into Human Erythrocytes. PLoS Pathog. *11*, e1005343.

Thomas, J.A., Tan, M.S.Y., Bisson, C., Borg, A., Umrekar, T.R., Hackett, F., Hale, V.L., Vizcay-Barrena, G., Fleck, R.A., Snijders, A.P., et al. (2018). A protease cascade regulates release of the human malaria parasite *Plasmodium falciparum* from host red blood cells. Nat. Microbiol. *3*, 447–455.

Tjhin, E.T., Spry, C., Sewell, A.L., Hoegl, A., Barnard, L., Sexton, A.E., Siddiqui, G., Howieson, V.M., Maier, A.G., Creek, D.J., et al. (2018). Mutations in the pantothenate kinase of *Plasmodium falciparum* confer diverse sensitivity profiles to antiplasmodial pantothenate analogues. PLoS Pathog. *14*, e1006918.

Tonkin, M.L., Roques, M., Lamarque, M.H., Pugnière, M., Douguet, D., Crawford, J., Lebrun, M., and Boulanger, M.J. (2011). Host cell invasion by apicomplexan parasites: insights from the co-structure of AMA1 with a RON2 peptide. Science *333*, 463–467.

Trager, W., and Jensen, J.B. (1976). Human malaria parasites in continuous culture. Science *193*, 673–675.

Triglia, T., Tham, W.H., Hodder, A., and Cowman, A.F. (2009). Reticulocyte binding protein homologues are key adhesins during erythrocyte invasion by Plasmodium falciparum. Cell. Microbiol. *11*, 1671–1687.

Triglia, T., Chen, L., Lopaticki, S., Dekiwadia, C., Riglar, D.T., Hodder, A.N., Ralph, S.A., Baum, J., and Cowman, A.F. (2011). *Plasmodium falciparum* merozoite invasion is inhibited by antibodies that target the PfRh2a and b binding domains. PLoS Pathog. *7*, e1002075.

Vaughan, A.M., Mikolajczak, S.A., Wilson, E.M., Grompe, M., Kaushansky, A., Camargo, N., Bial, J., Ploss, A., and Kappe, S.H. (2012). Complete *Plasmodium falciparum* liver-stage development in liver-chimeric mice. J. Clin. Invest. *122*, 3618–3628.

Volz, J.C., Yap, A., Sisquella, X., Thompson, J.K., Lim, N.T., Whitehead, L.W., Chen, L., Lampe, M., Tham, W.H., Wilson, D., et al. (2016). Essential Role of the PfRh5/PfRipr/CyRPA Complex during *Plasmodium falciparum* Invasion of Erythrocytes. Cell Host Microbe *20*, 60–71.

Wei, Z., Wang, W., Hu, P., Lyon, G.J., and Hakonarson, H. (2011). SNVer: a statistical tool for variant calling in analysis of pooled or individual next-generation sequencing data. Nucleic Acids Res. *39*, e132.

Weiss, G.E., Gilson, P.R., Taechalertpaisarn, T., Tham, W.H., de Jong, N.W., Harvey, K.L., Fowkes, F.J., Barlow, P.N., Rayner, J.C., Wright, G.J., et al. (2015). Revealing the sequence and resulting cellular morphology of receptor-ligand interactions during *Plasmodium falciparum* invasion of erythrocytes. PLoS Pathog. *11*, e1004670.

Weiss, D.J., Lucas, T.C.D., Nguyen, M., Nandi, A.K., Bisanzio, D., Battle, K.E., Cameron, E., Twohig, K.A., Pfeffer, D.A., Rozier, J.A., et al. (2019). Mapping the global prevalence, incidence, and mortality of Plasmodium falciparum, 2000-17: a spatial and temporal modelling study. Lancet *394*, 322–331.

Wong, W., Huang, R., Menant, S., Hong, C., Sandow, J.J., Birkinshaw, R.W., Healer, J., Hodder, A.N., Kanjee, U., Tonkin, C.J., et al. (2019). Structure of *Plasmodium falciparum* Rh5-CyRPA-Ripr invasion complex. Nature *565*, 118–121.

Zuccala, E.S., Gout, A.M., Dekiwadia, C., Marapana, D.S., Angrisano, F., Turnbull, L., Riglar, D.T., Rogers, K.L., Whitchurch, C.B., Ralph, S.A., et al. (2012). Subcompartmentalisation of proteins in the rhoptries correlates with ordered events of erythrocyte invasion by the blood stage malaria parasite. PLoS One *7*, e46160.

STAR★METHODS

KEY RESOURCES TABLE

REAGENT or RESOURCE	SOURCE	IDENTIFIER
Antibodies		
Rabbit polyclonal anti-PMX	This work	N/A
Rabbit polyclonal anti-SERA5	Fairlie et al., 2008	N/A
Mouse monoclonal anti-RAP1	Schofield et al., 1986	N/A
Rat monoclonal 3F10 Horseradish peroxidase anti-HA	Roche	Cat# 12 013 819 001: RRID:AB_2314622
Rabbit anti-AMA1	Coley et al., 2001	N/A
Rabbit polyclonal anti-msp1-19	This work	N/A
Rabbit polyclonal anti-Rh1(120kD)	Triglia et al., 2009	N/A
Mouse monoclonal 6F12 anti-Rh2	Triglia et al., 2011	N/A
Mouse monoclonal 2E8 anti-Rh4	Tham et al., 2010	N/A
Mouse monoclonal 2D6 anti-EBA140 tail	This work	N/A
Mouse monoclonal 1H5 anti-EBA140	This work	N/A
Rabbit polyclonal anti-EBA175 tail	Gilberger et al., 2003a	N/A
Rabbit polyclonal anti-EBA175	Reed et al., 2000	N/A
Rabbit polyclonal anti-EBA181 tail	Gilberger et al., 2003b	N/A
Mouse monoclonal 1F10 anti-Rh2	Triglia et al., 2011	N/A
Goat polyclonal anti-*Pb*UIS4	Biorbyt	Cat#orb11636
Rabbit polyclonal anti-*Py*$MSP1_{19}$ antiserum	BEI Resources	MRA-23, contributed by John H. Adams
Chicken polyclonal anti-Goat IgG, Alexa Fluor 488	ThermoFisher	Cat#A21467: RRID:AB_10055703
Goat polyclonal anti-Rabbit IgG, Alexa Fluor 633	ThermoFisher	Cat#A21070: RRID:AB_2535731
Biological Samples		
Human serum	Australian Red Cross Blood Service	19-05VIC-13
Human red blood cells	Australian Red Cross Blood Service	19-05VIC-13
Chemicals, Peptides, and Recombinant Proteins		
WM4	This work	N/A
WM5	This work	N/A
WM382	This work	N/A
WM856	This work	N/A
RPMI 1640 medium	GIBCO	CAT#23400021
Albumax II	ThermoFisher	CAT#11021029
amine-BCN	Sigma	CAT#745073
NHS-activated Sepharose 4 Fast Flow beads	Sigma	CAT#H8280
Ni-NTA resin	ThermoFisher	CAT#88222
MACS LS columns	Miltenyi Biotec	130-042-401
RPMI 1640 medium containing Pen/Strep	WEHI Media Preparation Service	N/A
Fetal Bovine Serum	Thermo	Cat#F9423-500ML Lot#17G477
XenoLight D-Luciferin	PerkinElmer	Cat#122799
Rh2N peptide: DABCYL-HSFIQEGKEE-EDANS	China Peptides	N/A
Rh2C peptide: DABCYL-GTASLVQITQYE-EDANS	China Peptides	N/A
Rh1 peptide: DABCYL-KPFFFIQLNTE-EDANS	China Peptides	N/A
Rh5 peptide: DABCYL-KNVNFLQYHFE-EDANS	China Peptides	N/A
Ripr peptide: DABCYL-GNISMLEIQNEE-EDANS	China Peptides	N/A
RAP1 peptide: DABCYL-GFSSESFLENKG-EDANS	China Peptides	N/A
SUB1 peptide: DABCYL-GSMLEVENDAEGE-EDANS	China Peptides	N/A

(*Continued on next page*)

Continued

REAGENT or RESOURCE	SOURCE	IDENTIFIER
SUB1 mutant peptide: DABCYL-GSMAAVENDAEGE-EDANS	China Peptides	N/A
RAP1 mutant peptide: DABCYL-GFSSESFAANKG-EDANS	China Peptides	N/A
EBA175 peptide: DABCYL-RILSFLDSRI-EDANS	China Peptides	N/A
EBA181 peptide: DABCYL-NRNSFVQRSYE-EDANS	China Peptides	N/A
EBA181 mutant peptide: DABCYL-NRNAFVARSYE-EDANS	China Peptides	N/A
Rh2N mutant peptide: DABCYL-HAFIAEGKEE-EDANS	China Peptides	N/A
ASP peptide: DABCYL-KFLSLLQLNLE-EDANS	China Peptides	N/A
Rh4 peptide: DABCYL-KILNSFVQINSSE-EDANS	China Peptides	N/A
RON3 peptide: DABCYL-KEISFLERRE-EDANS	China Peptides	N/A
Critical Commercial Assays		
flashBAC Baculovirus Expression System	Oxford Expression Technologies	Cat# 100150
Deposited Data		
Whole Genomic Sequences of WM4 and 5 resistant *P. falciparum* lines	European Nucleotide Archive	PRJEB36069
Experimental Models: Cell Lines		
HepG2	ATCC	Cat#HB-8065
Spodoptera frugiperda (sf21) insect cells	Sigma-Aldrich	Cat#05022801
Experimental Models: Organisms/Strains		
Plasmodium falciparum: 3D7-ASP-HA	Walter and Eliza Hall Institute	Zuccala et al., 2012
Plasmodium falciparum: 3D7-PMIX-HA	Walter and Eliza Hall Institute	This work
Plasmodium falciparum: 3D7-RON3-HA	Walter and Eliza Hall Institute	This work
Plasmodium falciparum: 3D7-MTRAP-HA	Walter and Eliza Hall Institute	This work
Plasmodium falciparum: 3D7-SUB2-HA	Walter and Eliza Hall Institute	Riglar et al., 2011
Plasmodium falciparum: 3D7-PMX-HA	Walter and Eliza Hall Institute	This work
Plasmodium falciparum: W2mef-PfRh1-HA	Walter and Eliza Hall Institute	Triglia et al., 2011
Plasmodium falciparum: 3D7-PfRh5-HA	Walter and Eliza Hall Institute	This work
Plasmodium falciparum: 3D7-PfRipr-HA	Walter and Eliza Hall Institute	This work
Plasmodium falciparum: 3D7-PfRh2a-HA	Walter and Eliza Hall Institute	Triglia et al., 2011
Plasmodium falciparum: $Pf3D7^{0087/N9}$	SwissTPH	Jimenez-Díaz et al., 2009
Plasmodium falciparum: NF54	Walter Reed Army Institute	N/A
Plasmodium knowlesi: YH1	Michael Blackman, Crick Institute	Moon et al., 2013
Plasmodium berghei: (ANKA) mcherry Luciferase (1868 clone 1)	Leiden University Medical Center	Prado et al., 2015
Plasmodium berghei: (ANKA) GFPcon 259cl2 (MRA-865)	Walter and Eliza Hall Institute	Franke-Fayard et al., 2004
Mouse: ASMU:Swiss	Monash Animal Research Platform	N/A
Mouse: NOD-*scid IL- 2Rγnull*	The Jackson Laboratory	RRID: BCBC_4142
Mouse: BALB/c	Clive and Vera Ramaciotti Laboratories	N/A
Mosquito: *Anopheles stephensi*	Johns Hopkins School of Public Health	N/A
Oligonucleotides		
Primers and guides, see Table S1	This paper	N/A
Software and Algorithms		
PlasmoDB	Bahl et al., 2003	https://plasmodb.org/plasmo/app/record/dataset/DS_fe9f5bc9d1
FastQC		https://www.bioinformatics.babraham.ac.uk/projects/fastqc/

(*Continued on next page*)

Continued

REAGENT or RESOURCE	SOURCE	IDENTIFIER
Trimmomatic	Langmead and Salzberg, 2012	http://www.usadellab.org/cms/?page=trimmomatic
MarkDuplicates (version 2.2.2).		http://broadinstitute.github.io/picard/
GRIDSS (versions 1.3 and 1.4)	Cameron et al., 2017	https://github.com/dotnet/standard/blob/master/docs/versions.md
Bowtie2 (version 2.2.5)[REMOVED HYPERLINK FIELD]	Langmead and Salzberg, 2012	https://sourceforge.net/projects/bowtie-bio/files/bowtie2/2.2.5/
SNVer (v0.5.3)[REMOVED HYPERLINK FIELD]	Wei et al., 2011	https://github.com/NCBI-Hackathons/FlowBio/wiki/Dependencies
VarScan (v2.4)[REMOVED HYPERLINK FIELD]	Koboldt et al., 2012	http://varscan.sourceforge.net/
QDNaseq (version 1.10.0)[REMOVED HYPERLINK FIELD]	Scheinin et al., 2014	http://bioconductor.org/packages/release/data/experiment/html/QDNAseq.hg19.html
Integrated Genome Viewer	N/A	http://software.broadinstitute.org/software/igv/
FlowJo for Mac, version 10.6	Becton Dickinson	N/A
Columbus Image Data Storage and Analysis System, version 2.8	PerkinElmer	N/A
Living Image version 4.7.2	PerkinElmer	N/A
GraphPad Prism (version 8.2.0)	N/A	www.graphpad.com

LEAD CONTACT AND MATERIALS AVAILABILITY

Further information and requests for resources and reagents should be directed to and will be fulfilled by the Lead Contact, Alan F. Cowman (cowman@wehi.edu.au).

All unique/stable reagents generated in this study are available from the Lead Contact with a completed Materials Transfer Agreement.

EXPERIMENTAL MODELS AND SUBJECT DETAILS

Ethics Statement

Use of human blood and serum was approved by the Walter and Eliza Hall Institute of Medical Research Human Ethics committee under approval number 19-05VIC-13. Use of animals was approved by the Walter and Eliza Hall Institute of Medical Research Animal Ethics Committee under approval numbers 2017.014 and 2019.013.

Plasmodium falciparum Culture

P. falciparum asexual blood stage parasite cultures and the parasite lines derived from these by genetic manipulation were grown in *in vitro* culture (Trager and Jensen, 1976). All parasites were supplied with O+ erythrocyte (Australian Red Cross Bloodbank, South Melbourne, Australia) at 4% hematocrit in Roswell Park Memorial Institute (RPMI) 1640 medium supplemented with 26 mM 4-(2-hydroxyethyl)piperazine-1-ethanesulfonic acid (HEPES), 50 μg/mL hypoxanthine, 20 μg/mL gentamicin, 2.9% $NaHCO_3$, 5% Albumax II^{TM} (GIBCO), and 5% heat-inactivated human serum. Cultures were incubated at 37°C in a gaseous mix of 94% N_2, 1% O_2 and 5% CO_2. Parasites were sub-cultured with new culture media every 2 days in order to maintain 4% hematocrit and keep the parasitemia below 5%

Plasmodium knowlesi Culture

Plasmodium knowlesi YH1 strain was cultured in RPMI-HEPES media supplemented with 2.3 g/L sodium bicarbonate, 5 g/L Albumax, 4 g/L dextrose and 0.30 g/L glutamine. *P. knowlesi* parasites were synchronized using nycodenz (Moon et al., 2013). All parasites were grown using O+ human erythrocytes (Australian Red Cross Bloodbank, South Melbourne, Australia)s

Plasmodium berghei Maintenance

P. berghei parasites were expanded in Swiss mice by infection intraperitoneally (IP) with 1×10^6 *P. berghei* ANKA-parasitised red blood cells from frozen stocks of infected blood or parasites withdrawn from a previously infected donor mouse.

P. falciparum Gametocyte Culture

NF54 *P. falciparum* cultures were synchronized and maintained as described earlier at 5%–10% parasitemia at 4% hematocrit. Once parasitemia reached 8%–10% rings, parasites were diluted to 0.65% parasitemia in desired volume for the gametocyte cultures (10 mL for Petri dish or 6 × 5 mL for 6-well plate). Gametocyte cultures were supplied with gametocyte media consisted of O+ erythrocyte at 4% hematocrit in Roswell Park Memorial Institute (RPMI) 1640 medium supplemented with 26 mM 4-(2-hydroxyethyl)piperazine-1-ethanesulfonic acid (HEPES), 50 μg/mL hypoxanthine, 2.9% $NaHCO_3$, and 10% heat-inactivated human serum. Sexual stage gametocytes were induced by allowing continuous growth cultures without the addition of fresh human erythrocytes (Vaughan et al., 2012). On day 4, a smear was taken from the parasite culture to observe gametocyte development, the crash of asexual stages and early gametocytes were observed during this period. The medium volume was increased (e.g., from 5 mL culture to 7.5 ml) and the gametocyte media was changed every day at 37°C on a slide warmer within the biosafety cabinet until the compounds to be tested were introduced on day 13. Stage V gametocytemia was determined by light microscopy examination of methanol fixed, Giemsa stained thin smear under 1,000 magnification. Stage V gametocytes were centrifuged at 13,000 rpm for 1 min to remove gametocyte media, 0.2% of stage V gametocytes were resuspended with the mixture of fresh uninfected erythrocytes and heat inactivated human serum at 2:3 ratio. During the process of blood meal preparation, all reagents used were maintained at 37°C slide warmer. Various concentration of compound WM382, starting from 0.5 nM, 1 nM, 2.5 nM, 25 nM, 50 nM, were included in the gametocyte media before being added to parasite culture daily from day 13 to day 17.

To prepare the mosquito feeding apparatus, Parafilm (Sigma-Aldrich) was stretched by hand in both the x and y planes and stretched across glass water-jacket feeders of appropriate size. Where large cages of mosquitoes were fed, 2 medium size feeders (Nasirian and Ladonni, 2006), as opposed to one large feeder were used to maintain 37°C throughout the bloodmeal. Glass feeders were connected to a circulating waterbath set to 38°C via plastic tubing or appropriate diameter, using step-up/step-down connectors where necessary. Feeders were positioned on the tops of cages and held in place with adhesive tape. The waterbath was turned on at least 10 min prior to feeding to attain the correct temperature. Mosquitoes were allowed to feed for 30 min.

On day 7 after feeding with gametocyte bloodmeal, infected mosquitoes were aspirated into a cup and anesthetized by incubating them at −20°C for 5 min until immobilized. The mosquitoes were killed by placing them in a Petri dish with 80% ethanol. Glass slides were prepared by transferring mosquitoes into a drop of PBS. Mosquitoes were stabbed at the thorax by needle tip and forceps were used to grab the last abdominal segment before pulling it off the mosquito abdomen. After the abdomen was removed, the midgut was detached from the mosquito thorax with forceps. Collected midguts were maintained in 1X phosphate buffered saline (PBS, GIBCO) until stained with mercurochrome for 20 min then placed on a glass slide and counted for oocysts by microscopy.

Mouse Models

ASMU-Swiss Outbred and BALB/c Mice

ASMU:Swiss outbred (pathogen-free), females, 4 weeks, 15 - 18 g or BALB/c (pathogen-free), 7-10 weeks, were kept in individually ventilated cages (IVC) or exhausted ventilated cages (EVC) with corncob bedding, but otherwise under standard conditions with 22°C and 40 – 70% relative humidity, Barastock Rat & Mouse pellet (WEHI Mouse Breeder Cubes, Irradiated (#102119) and Mouse custom Mash Irradiated (#102121), Ridley Agriproducts Pty Ltd) and water *ad libitum*. There is a maximum of 6 adults per cage with more than 1 mouse. The water provided to mice is filtered and acidified to pH3 and supplied in a Hydropac pouch or water bottle. Animal enrichment (e.g., tissues, domes) is provided each week when cages are serviced. Animal technicians are responsible for the daily husbandry of the mice and service each cage at least twice a week. Cages are cleaned on a rotating schedule (depending on the stocking density) or as often as needed. Typically, cages of 4 –6 mice require fortnightly cleaning and cages housing 1-3 mice require monthly cleaning.

Humanized NOD-scid IL2R_null Mice

SCID model (Angulo-Barturen et al., 2008). *NODscidIL2R*γ^{null} mice (severe combined immunodeficiency), females, 20 - 22 g were kept in individually ventilated cages (IVC), but otherwise under standard conditions with 22°C and 60 – 70% relative humidity, pellets (PAB45 – NAFAG 9009, Provimi Kliba AG, CH-4303, Kaiseraugst, Switzerland) and water *ad libitum*. Husbandry was the same as described above for ASMU-Swiss outbred and BALB/c mice.

Anopheles stephensi *Colony Maintenance and Sporozoite Production*

Anopheles stephensi mosquitoes were originally imported from Johns Hopkins School of Public Health, reared and maintained in the insectary at the Walter and Eliza Hall Institute according to standard methods. The mosquitoes were stored in BugDorm insect cages, mixed genders with an environment of 26-27°C, relative humidity 70%–80%, light cycle 12 h/12 h including 30 min of ramping up or down between dark and light to imitate dawn and dusk. They were fed on reverse osmosis filtered water via cotton wicks and sugar cubes (sucrose- CSR).

To begin an infection cycle, one ‘donor’ BALB/c mouse was injected i.p. with blood stage *P. berghei* parasites constitutively expressing mCherry and Luciferase reporters (*Pb*mCherryLuci) (Prado et al., 2015). Three days later, three ‘acceptor’ BALB/c mice were injected i.p. with 2.5 × 10^5 infected erythrocytes from the ‘donor’ mouse. Acceptor mice were anesthetised using ketamine/xylazine and fed to 5 to 7-day old female *A. stephensi* mosquitoes when they reached 1%–3% parasitemia. Midgut oocyst numbers were enumerated in 15 – 30 mosquitoes between day 14 - 17 post bloodmeal to determine the percent infected mosquitoes. Salivary gland sporozoites were dissected from mosquitoes into Schneider’s Insect Media (Sigma-Aldrich) pH 7.0 to enhance viability (Roth et al., 2018) between day 18 – 26 post bloodmeal.

HepG2 Cell Culture

HepG2 (supplied by ATCC, Cat#HB-8065) cells were maintained in RPMI1640 supplemented with pen/strep and 10% Fetal Bovine Serum ('complete medium') (Thermo Fisher) and seeded at 3 × 10^4 cells in black wall, ultra-thin bottom 96-well plates (Greiner μClear) using standard methods the day before infection. The cells were passaged when 90% confluent.

METHOD DETAILS

Construction of *P. falciparum* Lines Expressing HA-Tagged Proteins

All *P. falciparum* genetically engineered parasites made for this work (see below) were constructed using CRISPR/Cas9 methods as described (Ghorbal et al., 2014).

Construction of HA-glmS-tagged P. falciparum: 3D7-PMX-HA and 3D7-PMIX-HA *P. falciparum* lines were derived from 3D7 and designed to have a HA epitopes at the 3′ end of the gene with a *glmS* 3′ noncoding region to enable knockdown of enzyme expression. The gene *PfPMX* (accession number: PF3D7_0808200) was HA-glmS-tagged using the 1.2HLG3 vector. The 3′ homology region (496 bp) was amplified from 3D7 genomic DNA using the oligonucleotides TT969 and TT970 and cloned using the EcoRI/PstI restriction sites. An amplicon of 510 bp amplified using oligonucleotides TT967 and TT968 was fused to codon-optimized PMX for the rest of the gene. Oligonucleotides encoding the guide sequence TT979 were cloned into pUF1-cas9G by InFusion technology. The plasmids for homology-directed repair (HDR) and the guide plasmid were transfected simultaneously into *P. falciparum* PEMS obtained by E64 treatment following a previously published method (Volz et al., 2016).

HA-glmS-tagged *P. falciparum PfPMIX* (accession number: PF3D7_1430200) was generated following the same strategy. The 3′ homology region was amplified from 3D7 genomic DNA with the oligos PF3 and PF4. The amplicon (624 bp) was cloned into the 1.2HLG3 vector by the restriction sites EcoRI/PstI to create the pPMIXHA-glmS_3′ vector. The 5′ homology and codon-optimized *plasmepsin IX* gene region (939 bp) was produced by GeneArt (Life Technologies) and cloned into pPMIXHA-glmS_3′ vector using the *Not*I/*Xho*I restriction sites to produce the pPMIXHAglmS_CRISPR vector. The guide oligos PF1 and PF5 were designed to induce a double-strand break in PMIX at genomic position 2161 bp.

Construction of P. falciparum expressing HA-tagged RON3, MTRAP, Rh5 and Ripr: P. falciparum parasite lines expressing RON3-HA, MTRAP-HA, Rh5-HA or Ripr-HA were constructed in the same way as described above for PMX- and PMIX-HA tagged parasite lines using CRISPR/Cas9 (Ghorbal et al., 2014). *RON3* (accession number: PF3D7_1252100), *MTRAP* (accession number: PF3D7_1028700), *Rh5* (accession number: PF3D7_0424100) and *Ripr* (accession number: PF3D7_0323400) 5′ and 3′ flanks were obtained using the oligonucleotide primers described above and cloned into the 1.2HLG3 vector. Oligonucleotides encoding the guide sequences were cloned into pUF1-cas9G by InFusion technology. The plasmids for homology-directed repair (HDR) and the guide plasmid were transfected simultaneously into *P. falciparum* PEMS obtained by E64 treatment following a previously published method (Volz et al., 2016). Expression of HA-tagged proteins were confirmed for each parasite line using anti-HA antibodies.

The construction of 3D7-ASP-HA (Zuccala et al., 2012), 3D7-SUB2-HA (Riglar et al., 2011), W2mef-PfRh1-HA (Triglia et al., 2011) and 3D7-PfRh2a-HA (Triglia et al., 2011) have been described elsewhere.

High Throughput Screening and EC_{50} Determination of *P. falciparum* Growth

For High Throughput Screening (HTS) of compounds we used the lactate dehydrogenase (LDH) assay to detect parasitemia (Makler et al., 1993; Oduola et al., 1997) formatted in a 384 well assay using an Echo555 (Labcyte). The *P. falciparum* assay was conducted with minor modifications (Gamo et al., 2010). Briefly, an inoculum of parasitized red blood cells at 0.7% parasitemia and 2% hematocrit in RPMI-1640, 5% AlbuMAX, 2% d-sucrose, 0.3% glutamine and 150 μM hypoxanthine was used for the assay. Assay plates (Greiner #781098, 384 well, white, tissue culture treated) were prepared by dispensing 1 μl of compound from master plates at 20 μM (10% DMSO stock) into 9ul pre-dispensed growth media in each well. The parasite inoculum (30 μl) was dispensed into plates containing compounds using a Multidrop Combi dispenser (Thermo Scientific). Final assay volume was 40 μl and final compound concentration was 500 nM. The positive growth control was 0.25% DMSO and the negative growth control was 100 nM chloroquine. Plates were incubated at 37 °C for 72 h in an atmosphere of 5% CO2, 5% O2, 95% N2. Following 72 h growth, plates were sealed with parafilm and frozen at −80 °C overnight. Plates were thawed at room temperature for at least 4 h prior to LDH activity being measured. 45 μl of fresh LDH reaction mix (174 mM sodium l-lactate, 214 μM 3-acetyl pyridine adenine dinucleotide (APAD), 270 μM Nitro Blue tetrazolium chloride (NBT), 4.35 U ml-1 diaphorase, 0.7% Tween 20, 100 mM Tris-HCl pH 7.5) was dispensed into each well using a Multidrop Combi dispenser. Plates were shaken to ensure mixing and absorbance at 650 nm was measured in an EnVision (PerkinElmer) plate reader after 30 min of incubation at room temperature. Data were normalized to percent growth inhibition using positive and negative controls and analyzed using Dotmatics 5.3 and Spotfire 7.11.1 (Tibco) software.

To eliminate the selection of false positive hits inhibiting the biochemical readout system, the primary hits were assayed against bovine recombinant LDH activity (12.5 U ml-1, Sigma L3916) under identical reaction conditions.

To calculate active compounds for rescreening a 10-point dilution series of the compounds were prepared in 384 well assay plates using an Echo555 (Labcyte). Appropriate volumes of 10 mM compound stocks were transferred into the assay plates such that the starting concentration was 1 μM or 375 nM, with a 1:3 fold dilution series. All wells were backfilled with DMSO to 45 nL such that this remained constant (0.1% DMSO). The positive growth control was 0.1% DMSO and the negative growth control was 100 nM chloroquine. Parasite inoculum (40 μl) was dispensed into plates containing compounds as described above. All other culture and lactate dehydrogenase (LDH) reaction methods are as described previously (Makler et al., 1993; Oduola et al., 1997). EC_{50} values were

calculated by Dotmatics 5.3 and Spotfire 7.11.1 software using a nonlinear regression four-parameter fit analysis. The equation used is sigmoidal dose response (variable slope), Y = bottom + (top − bottom)/(1+10((log EC_{50} − X) × Hill Slope)).

For all other experiments determination of EC_{50} was obtained using ring stage parasites at 0.5% parasitemia which were dispensed in a 50 μL culture at 2% hematocrit in 96 well round bottom microtiter plates (Falcon) with doubling dilutions of each compound. After 72 h incubation at 37°C each well was fixed at room temperature for 30 min with 50 μL of 0.25% glutaraldehyde (ProSciTech) diluted in PBS. Following centrifugation at 1200 rpm for 1 min, supernatants were discarded and parasites were stained with 50 μL of 5X SYBR Green (Invitrogen) diluted in PBS. 50,000 cells were counted by flow cytometry using a Cell Lab Quanta SC – MPL Flow Cytometer (Beckman Coulter) (Bacon et al., 2007). Growth was expressed as a percentage of the parasitemia obtained using a vehicle control. All samples were tested in triplicate. EC_{50} values were determined using GraphPad Prism.

Determination of EC_{50} in Knock down Parasite Lines

Ring stage *P. falciparum* 3D7, 3D7-PMIX-HA and 3D7-PMIX-HA parasites were cultivated with increasing concentrations of GlcN (Sigma). After 72 h incubation at 37°C, trophozoite-infected erythrocytes were lysed in 0.06% saponin, pellets were solubilized in 2 times reducing SDS-PAGE sample buffer and analyzed by anti-HA immune-detection. EC_{50} of inhibition for WM4 and WM382 against *P. falciparum* 3D7, 3D7-PMIX-HA and 3D7-PMX-HA parasites, were determined in the absence and presence of 2.5 nM GlcN (normal and reduced protein expression of HA-tagged protein, respectively) by FACS determination and SYBR Green, as already described.

P. knowlesi Drug Sensitivity Assay

The drug sensitivity assays were performed in 96-well plates using 50 μL culture at 0.5% parasitemia and 2% hematocrit. The compounds were serially diluted in 1% DMSO. Parasite growth assays were performed for 48 h. Parasite cultures were then fixed with 50 μL 0.25% glutaraldehyde for 30 min at room temperature. The culture supernatants were removed and the cells were stained with 5% SYBR Green in the dark for 30 min. Parasitemia was defined by the proportion of Alexa 488-A positive cells in 50,000 recorded events using LSR II analyzer (BD). The percentage of parasite growth was normalized with the DMSO vehicle control group and the highest-dosage group, and plotted as a dose-response curve in Prism 7.

In Vitro Selection of Parasites Resistant to WM4 and WM5

Three replicate cultures of clonal 3D7 parasites were grown on incremental increases of compound beginning at a concentration of 2x $EC_{50.}$ When parasitemia was significantly reduced, compound pressure was removed and when parasitemia recovered, compound pressure was resumed. This was repeated until the parasites were adapted to a concentration of compound of 3-5x EC_{50}. The parent and resistant lines were then cloned by limiting dilution, the EC_{50} for each line was determined and genomic DNA was purified from clonal parasites (QIAGEN Blood and Tissue Kit).

Genome Sequencing and Bioinformatics Analysis

An input of 200 ng of *P. falciparum* genomic DNA was prepared and indexed for illumina sequencing using the TruSeq DNA sample Prep Kit (illumina) as per manufacturer's instruction. The library was quantified using the Agilent Tapestation. The indexed libraries were pooled and diluted to 1.5 pM for paired end sequencing (2x 81 cycles) on a NextSeq 500 instrument using the v2 150 cycle High Output kit (illumina) as per manufacturer's instructions. The base calling and quality scoring were determined using Real-Time Analysis on board software v2.4.6, while the FASTQ file generation and de-multiplexing utilized bcl2fastq conversion software v2.15.0.4.

The quality of sequencing was confirmed using FastQC, and where Illumina adaptor contamination was detected it was removed with Trimmomatic (v0.36). Resulting fastq files were aligned to the 3D7 reference genome (PlasmoDB-29_Pfalciparum3D7) using Bowtie2 (version 2.2.5)[REMOVED HYPERLINK FIELD] (Langmead and Salzberg, 2012) with parameter-sensitive-local. Duplicate reads were removed using Picard tools MarkDuplicates (version 2.2.2). Calling of single nucleotide variants (SNVs) and indels was performed with SNVer (v0.5.3)[REMOVED HYPERLINK FIELD] (Wei et al., 2011) and VarScan (v2.4)[REMOVED HYPERLINK FIELD] (Koboldt et al., 2012). Copy number analysis was performed using the R package QDNaseq (version 1.10.0)[REMOVED HYPERLINK FIELD] (Scheinin et al., 2014). Structural variant calling was performed using GRIDSS (versions 1.3 and 1.4)[REMOVED HYPERLINK FIELD] (Cameron et al., 2017). Regions of interest were inspected with Integrated Genome Viewer.

Southern Blot Analysis

Genomic DNA from clonal parent and resistant parasite lines was digested with restriction endonucleases *Hind* III and *Pac* I (NEB), fractionated by agarose electrophoresis and transferred to Hybond-N nylon membrane (GE Healthcare). D*igoxigenin* (*DIG*) labeled DNA probes to detect PMX and EBA175 were produced using a PCR DIG Probe Synthesis Kit (Roche). For PMX, primers CATCATGAGTCTCTAAAATTAGGGGACG and CACTCTCTACTAATCCAAAAGTCTG amplified a 790 bp probe to detect a 3.4 kb *Hind* III restriction fragment. For EBA175, primers CAAGAAGCAGTTCCTGAGGAAA and CCCAGAATTTCCCCCCCGATCCTG amplified a 1614 bp probe to detect a 4.6 kb *Hind* III/*Pac* I restriction fragment. Hybridization was carried out with both probes simultaneously for 16 h at 40°C in DIG Easy Hyb (Roche). A DIG Luminescent Detection Kit (Roche) was used for blocking and detection according to manufacturer's instructions followed by exposure to X-ray film (Fuji).

PMX Pulldown with WM856

The amine-BCN (Sigma #745073) was immobilized onto NHS-activated Sepharose 4 Fast Flow beads (Sigma #H8280) as described (Ferreri et al., 2010). Briefly, 650 μL slurry of NHS-Sepharose beads was washed twice with 5 mL DMSO, centrifuging at 80 x g for 3 min to pellet the matrix in between washes. One packed matrix volume (325 μL) was resuspended with DMSO as a 50% slurry. Amine-BCN (2 μM final) was added to the 650 μL slurry of NHS-beads followed by 15 μL of triethylamine and mixed by inversion. The reaction slurry was incubated overnight at room temperature on an end-over-end rotator protected from light. The following day, 20 μL ethanolamine was added to the reaction and incubated overnight at room temperature on an end-over-end rotator protected from light. The BCN-coupled NHS-Sepharose beads were washed twice with 5 mL DMSO and the matrix was resuspended in ethanol and stored at 4°C protected from light.

WM856 (azide-functionalized imino pyrimidinone compound) at 2 μM final in a mixture of CH_3CN/H_2O (3/1) was added to the BCN-coupled NHS-Sepharose beads and incubated overnight at room temperature on an end-over-end rotator protected from light. The WM856-triazole-coupled NHS-Sepharose beads were washed twice with 5 mL DMSO and the matrix was resuspended in ethanol and stored at 4°C protected from light.

WM856–coupled Sepharose beads were washed twice with TNET lysis buffer (150 mM NaCl, 50 mM Tris-HCl pH 7.4, 1% (v/v) Triton X-100, 10 mM EDTA) before protein enrichment. Samples for pulldown experiments were prepared using highly synchronous schizont stage *P. falciparum* 3D7-PMX_HA parasites. Late-stage parasites (40–44 h post-invasion) were purified by Percoll density gradient and incubated with 1 μM compound 1 (C1) inhibitor (synthesized in-house) (Hale et al., 2017). After 2–4 h incubation, mature schizonts were washed once with PBS and pelleted infected red blood cells were lysed in 20 volumes of 0.15% (w/v) saponin in PBS and incubated on ice for 20 min. Parasites were washed 3 times in ice-cold PBS and the final pellet was resuspended in 10 volumes of TNET lysis buffer and incubated for 30 min at 4°C on a rotator. The lysates were cleared by centrifugation at 16,000 g for 30 min and the supernatant containing soluble parasite proteins kept at −80°C until use. Three individual protein enrichments were performed (2 μM WM856 in DMSO vehicle, 2 μM WM856 + 0.2 μM WM382 as competitor, or DMSO vehicle only) with 160 μL of 50% WM856–coupled Sepharose beads incubated with 500 μL of whole-cell lysate. Incubations were performed for 3 h on a rotating wheel protected from light at 4°C. Following incubation, protein-WM856–couples Sepharose beads were washed 3 times with TNET buffer and eluted with 3 consecutive rounds of incubation with 2x reducing NuPAGE LDS Sample Buffer (200 μL, 100 μL, 100 μL) for 3 min at 60°C. Proteins were resolved on precast 4%–12% gradient gels (Thermo Fisher Scientific) with MES running buffer (Thermo Fisher Scientific) according to the manufacturer's directions. The proteins were electrophoretically transferred to nitrocellulose membrane using a dry-blotting system (iBlot, Thermo Fisher Scientific). After blocking the membrane in 5% non-fat milk in TBST (M-TBST), PMX_HA was detected with anti-HA mAb 12CA5 (produced in-house) followed by HRP-conjugated a-mouse IgG Abs (Merck). Blots were developed with ECL Western Blotting Substrate (Thermo Fisher Scientific), and the images were generated and analyzed using ChemiDoc Gel Imaging System and Image Lab Software (Bio-Rad).

PMX Expression and Purification

The sequence of the *P. falciparum* PMX was obtained from the NCBI Gene database and cloned into the pACGP67A vector containing a C-terminal 8xHis tag. Recombinant Baculovirus containing the *P. falciparum* PMX sequence was generated using the flashback system (Oxford Expression Technologies) by following the manufacturer's instructions. Recombinant PfPMX was expressed in *Spodoptera frugiperda* (sf21) insect cells as a secreted protein (cells were purchased commercially from Sigma Aldrich (Cat#05022801). For the expression culture with L-WM382, a 3 μM final compound concentration was supplemented with the addition of Baculovirus. The supernatant was harvested, centrifuged and partially purified using Ni-NTA resin (ThermoFisher). Protein elution was performed by applying 15 mM imidazole increments from 10 to 200 mM in (20 mM Tris pH 7.5, 300 mM NaCl). PMX-containing fractions were further purified by gel filtration using a Superdex 200 10/300 column (GE Healthcare) in 20 mM HEPES pH 7.5, 150 mM NaCl.

Identification of Potential PMX and PMIX Substrates

Plasmepsin X cleavage sites were identified in Rh2a/b N-terminal & Rh2b C-terminal proteins by purification and Mass Spectrometry (Figure S7) and N-terminal sequencing (data not shown). These data showed that the Rh2a/b proteins were cleaved at 'SFIQ & 'SLVQ' motifs respectively (Figure S4). These cleavage sites were similar to those that have now been published for AMA1 and SUB1 (Pino et al., 2017). Further affinity purification & Mass Spectrometry of processed Rh1, Ripr and Rh5 proteins (Figure S7), showed that these also were cleaved at a 4 amino acid motif with similarity to those previously seen in the Rh2 proteins (Figure S4). Since Rh2a/b proteins were assumed to be stored in the microneme prior to being transported to the merozoite apical tip, we reasoned that other micronemal proteins may also be PMX cleaved. This list included Rh1, Rh4, Rh5, Ripr, EBA140, EBA175, EBA181 and AMA1, which has now been shown to be PMX cleaved (Pino et al., 2017). Inspection of their protein sequences, showed that all these proteins had one or more potential PMX cleavage sites. Hence, we obtained or produced HA-tagged parasites in cases where we had no Abs to the proteins or used in-house Abs to other proteins. We used these parasite and Ab reagents to prove that these micronemal proteins were processed by PMX and this processing could be blocked by both WM4 and WM382 drugs.

FRET Based Assay for PMX Cleavage of Peptide Substrates

Synthetic fluorogenic peptides corresponding to *P. falciparum* sequences of PMX substrates were synthesized by ChinaPeptides. Substrate cleavage assays were performed by incubating fluorogenic peptides (5 μM) in 25 mM ammonium acetate buffer (pH 5.5) with and without PMX at 37°C for 2 h. Samples were excited at 340 nm and fluorescence emission was measured at

492 nm using an Envision fluorescence plate reader (Perkin-Elmer) heated at several time points over 2 h. The measurement at 45 min (within the linear kinetic phase) was used for presentation of data.

For PMX cleavage site identification, fluorogenic peptides (50 μM) were incubated in 25 mM ammonium acetate buffer (pH 5.5) with and without PMX (50 nM) at 37°C for 20 h. Reactions were halted by passing samples through 10 kDa spin columns (abcam), which collect the enzyme within the column. The eluant was analyzed on an Agilent LC-ESMS system composed of an Agilent G6120B Mass Detector, 1260 Infinity G1312B Binary pump, 1260 Infinity G1367E HiPALS autosampler and 1260 Infinity G4212B Diode Array Detector MS using an Orbitrap LTQ mass spectrometer. Conditions for LCMS were as follows, column: Poroshell 120 EC-C18, 2.1 × 50 mm 2.7 Micron at 20°C, injection volume of 2 μL, with a gradient of 5%–100% B over 5 min (solvent A: water 0.1% formic acid; solvent B: AcCN 0.1% formic acid), with a flow rate of 0.8 mL/min and detection at 214 or 224 nm.

CETSA Thermal Stability Assays

Lysate CETSA experiments were conducted essentially as described (Dziekan et al., 2019). Samples for CETSA studies were prepared using highly synchronous schizont stage *P. falciparum* 3D7-PMIX_HA and 3D7-PMX_HA parasites. Late-stage parasites (40–44 h post-invasion) were purified by Percoll density gradient and incubated with 1 μM compound 1 (C1) inhibitor (synthesized in-house) (Hale et al., 2017). After 2–4 h incubation, mature schizonts were washed once with PBS and pelleted infected red blood cells were lysed in 20 volumes of 0.15% (w/v) saponin in PBS and incubated on ice for 20 min. Parasites were washed 3 times in ice-cold PBS and the final pellet was resuspended in 10 volumes of lysis buffer (0.4% NP-40 (Roche) / PBS) and lysed by three freezing (dry ice/ethanol bath) - thawing cycles. The lysates were cleared by centrifugation at 16,000 g for 30 min and the supernatant containing soluble parasite proteins kept at −80°C until use. Compounds 601 (5 μM), WM4 (2 μM) and WM382 (1 μM) were added to 8x 50 μg protein lysate aliquots (protein concentration measured by the BCA Protein Assay (Thermo Fisher Scientific), incubated at room temperature (RT) for 3 min and heated at respective temperatures (temperature gradient 65-40°C) for 3min in a Biorad T100 thermocycler, followed by 3 min incubation at 4°C. The post-heating lysates were centrifuged at 13,000 g for 30 min at 4°C. Soluble proteins were resolved on precast 4%–12% gradient gels (Thermo Fisher Scientific) with MES running buffer (Thermo Fisher Scientific) according to the manufacturer's directions. The proteins were electrophoretically transferred to nitrocellulose membrane using a dry-blotting system (iBlot, Thermo Fisher Scientific). After blocking the membrane in 5% non-fat milk in TBST (M-TBST), PMIX_HA and PMX_HA were detected with anti-HA mAb 12CA5 (produced in-house) followed by HRP-conjugated a-mouse IgG Abs (Merck). Blots were developed with ECL Western Blotting Substrate (Thermo Fisher Scientific), and the images were generated and analyzed using ChemiDoc Gel Imaging System and Image Lab Software (Bio-Rad).

Time of Drug Killing

3D7 parasite cultures were synchronized using 5% sorbitol (Sigma) twice at 46 h intervals then again when the culture was a mix of late schizonts and early rings. Triplicate 10ml cultures containing either 80nM WM4, 40nM WM5, 5nM WM382 or DMSO (Sigma) vehicle control were set up at 3% rings and 4% hematocrit. The parasitemia of each culture was quantitated every 8 h for 48 h by collecting 50 μL samples for counting by flow cytometry (as previously described). The developmental stage of the parasites was confirmed at each time point by microscopic examination of Giemsa stained thin blood films. Media and compound were replaced at the 32 h time point.

Invasion Assay

3D7 parasite culture were synchronized by sorbitol treatment and WM4 (40 nM) and WM382 (2.5 nM), or DMSO control, added at ring stage. Late-stage parasites (> 40 h post invasion) were enriched by magnet separation (MACS; Miltenyi Biotec) and allowed to develop to fully segmented schizont-stage parasites. To prevent schizont rupture and merozoites release, control parasites were incubated with 1 μM compound 1 (C1). After 5–6 h of incubation, parasitophorous vacuole membrane enclosed merozoites (PEMS) were pelleted, resuspended in a small volume of complete culture medium (containing WM4, WM382 or C1) and filtered through a 1.2 μm syringe filters (Acrodisc; 32 mm; Pall). Filtrate containing purified merozoites was immediately added to fresh erythrocytes (70%–80% hematocrit), incubated in a shaker (1,100 rpm) at 37°C for 20 min to allow invasion of host cells, and then diluted to 2% hematocrit. After 24 h incubation at 37°C, invasion was evaluated by measuring parasitemia by microscopy (Giemsa-stained thin smears) and flow cytometry (100,000 cells counted with FACSCalibur, BD).

Ring Stage Survival Assay for Artesunate

The method for determining sensitivity to artesunate was as described (Kite et al., 2016). Parasites were synchronized twice with 5% sorbitol at 46 h intervals and again when they were a mixture of schizonts and early rings. Triplicate 1 mL cultures were set up in 24 well plates (Falcon) at 0.5% parasitemia and 2% hematocrit with either 700 nM artesunate (Sigma) or 0.1% DMSO vehicle control. Following 6 h incubation, the cells were washed and incubated in drug-free media for a further 66 h. A 50 μL sample of each culture was fixed with 0.25% glutaraldehyde and stained with 5X SYBR then parasitemia was determined by flow cytometry. Percentage survival is parasitemia relative to vehicle treated parasites.

The percentage survival of all parasite lines was high in our experiments (~5% for 3D7) was high compared to that described previously (Kite et al., 2016) most likely because we used a FACS based method was used for detection and quantitation. However, we were clearly able to distinguish the resistant CAM3 R539T line from the sensitive CAM3 Rev and 3D7 parasite lines.

Determination of Time to Resistance

For experiment 1, cultures of 10^5, 10^6, 10^7, 10^8 and 10^9 Dd2 parasites were exposed to either 10 nM atovaquone (Sigma) or 1.5 nM WM382 and monitored for 90 days. For experiment 2, triplicate cultures of 10^6, 10^7 and 10^8 Dd2 parasites were exposed to either 5 nM atovaquone, 80 nM WM5 or 1.5 nM WM382 and monitored for 62 days. Each experiment was monitored by weekly microscopic examination of thin blood films. Media and compound were replaced three times each week.

P. falciparum Competition Growth Assays

Competition growth assays were determined by two independent methods. First, the 3D7 parent was mixed 1:1 with a parasite line resistant to either compound WM4 or compound WM5 and grown for 28 days as previously described (Tjhin et al., 2018). At day 0, day 14 and day 28 the EC_{50} of the parent, the resistant parasite line and the mixed culture was determined by flow cytometry as described elsewhere.

Second, *P. falciparum* 3D7 DiCre and 3D7 PMX amplification parasites were synchronized with 5% sorbitol twice, 8 h apart. The next day, parasitemia of trophozoite infected RBCs was determined by fluorescence-activated cell sorter (FACS) using a FACSCalibur (BD) analyzer and was adjusted to 0.5% parasitemia and 4% hematocrit. Once the parasitemia had been adjusted to 0.5%, the parasitemia was then rechecked by FACS to confirm the correct parasitemia before the assay was set up. Trophozoite infected RBCs were used to set up the assay. Assays were set up in the same dish containing a parasitemia of 0.5% of 3D7 DiCre and 0.5% of 3D7 PMX amplification parasites. Three independent experiments were set up. The mixed cultures were maintained at 0.5% parasitemia and 4% hematocrit by adding fresh erythrocytes every 2 days at which time genomic DNA (gDNA) was extracted from the sample using standard methods. The mixed culture was maintained over 6 weeks. To measure the relative abundance of each parasite line in the mixed culture gDNA was analyzed by real-time PCR using a LightCycler 480 real-time PCR system (Roche). Fifty nanograms of gDNA, 300nM of each primer was used in a 10 μL PCR reaction using SensiFAST SYBR green (bioline) and all PCR reactions were performed in duplicate. The PCR conditions consisted of an initial incubation at 95°C for 3 min and then 45 cycles at 95°C for 5 s, 58°C for 10 s, and 72°C for 10 s. Fluorescence was acquired at the end of each extension phase, and melting curve analysis was performed on each PCR reaction to determine the specificity of amplification. Each PCR amplicon (Pf Aldolase, DiCre, and PMXamplification) was cloned into TOPO-TA (ThermoFisher) to generate standard curves for each gene (serial 10-fold dilutions across a 6 log range). The amount of each target gene was estimated using the standard curve and was used to measure the copy number of genes for each sample. The primers (sequences) for target genes were as follows: Pf Aldolase forward primer (5′-TTGAACACATGGCAA GGAAA-3′), Pf Aldolase reverse primer (5′- ATTTTCACCACCTGCACCTC-3′), DiCre forward primer (5′-CGGGTCAGAAA GAATGGTGT-3′), DiCre reverse primer (5′-TGATTTCAGGGATGGACACA-3′), Pf PMXamplification forward primer (5′- TTGAA GAATGCCTTTTCATTTT-3′), and Pf PMXamplification reverse primer (5′- TGGTTTAGGGATGAGGGTTA-3′). Primers for the DiCre gene were designed to amplify in 3D7 DiCre parasites only, and primers for Pf PMXamplification were designed to amplify the 3D7 PMX amplification parasites. The relative concentration of target genes was normalized to that of the Pf Aldolase gene at the day 3. Using the normalized data for the target gene over those for Pf Aldolase generated the relative concentration of the copy number for the different parasite lines.

Peters' 4-Day Suppressive Test for *P. berghei* Infection in Mice

For results shown in Figure 1 A male Swiss mice were infected intraperitoneally (IP) with 1 × 10^6 *P. berghei* ANKA-parasitised red blood cells withdrawn from a previously infected donor mouse. Test compounds were prepared in a vehicle consisting of 10% DMSO/90% Solutol (5% Solutol® HS-15 in 0.9% saline). Two h post infection, mice were treated on 4 consecutive days (*q.d.* regimen, once a day) with an IP dose of test compounds (WM4, WM5: 20 mpk) or chloroquine (10 mpk), or received an IP injection of vehicle as a control. Peripheral blood samples were taken 24 h after administration of the last dose, and parasitemia was measured by microscopic analysis of Giemsa-stained blood smears. Parasitemia values were averages for 6 mice per group and are expressed as percent parasitemia. For results shown in Figure 4A 'donor' female Swiss mice were infected intraperitoneally (IP) with blood stage *P. berghei parasites* constitutively expressing GFP *(P. berghei ANKA GFPcon 259cl2* (Franke-Fayard et al., 2004). Three days later, groups of 4 'acceptor' Swiss mice were infected intravenously (IV) with 1 × 10^7 parasitised erythrocytes from the 'donor' mice. Two h post infection, experimental mice (4 per cohort) were left untreated (control mice) or treated orally on 4 consecutive days with test drugs formulated in 20% DMSO/60% PG/20% water (v/v/v) or chloroquine dissolved in water. Mice were treated with WM382 for 4 days by a *b.i.d.* dosing regimen (twice a day) at 20 mpk/day, with the first dose given 2 h after infection. Peripheral blood samples were taken 12 h after the last treatment, and parasitemia measured by flow cytometry (proportion of GFP-positive cells in 100,000 recorded events using FACSCalibur, BD) and microscopic analysis of Giemsa-stained blood smears. Parasitemia values were averages for 4 mice per group and are expressed as percent parasitemia.

Dose Ranging Test for *P. berghei* Infection in Mice

In the dose ranging studies, mice were treated orally with WM382 for 4 days by a *b.i.d.* dosing regimen at 30, 10, 3 or 1 mpk/day (Figure 4B), or by a *q.d.* dosing regimen at 60, 30 or 10 mpk/day (Figure 4C), with the first dose given 2 h after infection. Control mice were treated orally with chloroquine for 4 days under *q.d.* dosing regimen at 10 mpk/day. From day 2 to 30 post infection, parasitemia was measured daily by flow cytometry and microscopy, as described above. Survival of animals to day 30 post infection, with no detectable parasites in the peripheral blood, were considered to be cured (i.e., 100% efficacy).

P. falciparum Humanized NOD-*scid* IL2R_null Mouse Model

Compounds were tested in the murine *P. falciparum* SCID model (Angulo-Barturen et al., 2008). Briefly, WM382, formulated in 20% DMSO, 60% propylene glycol and 20% water, was administered to a cohort of age-matched female immunodeficient NOD-*scid IL- 2Rγnull* mice (The Jackson Laboratory, Bar Harbor, ME) previously engrafted with human erythrocytes (generously provided by the Blood Bank in Zürich, Switzerland). The mice were intravenously infected with 2 × 10^7 *P. falciparum* Pf3D7-infected erythrocytes (day 0) (Angulo-Barturen et al., 2008). On day 3 after infection, mice were randomly allocated to treatments that were administered once a day for 4 consecutive days (n = 3 mice) by oral gavage at 10 mL/kg. Parasitemia was measured by microscopy. Chimerism was monitored by flow cytometry using anti-murine erythrocyte TER119 monoclonal antibody (PharMingen, San Diego, CA) and SYTO-16 and then analyzed by flow cytometry in serial 2 μL blood samples taken every 24 h until assay completion.

Bioanalytical Determination of W382 in Mouse Blood Samples by LC-MS/MS

Serial samples of peripheral blood (25 μL) were taken from the mice of the efficacy experiment in the murine *P. falciparum* SCID model by tail puncture at 1, 2, 4, 6 and 24 h post first and last drug administration. The samples were immediately lysed by mixing with 25 μL of water, immediately frozen on dry ice and stored at −80°C until bioanalysis (carried out at SBQ, Reinach, Switzerland). The compounds were extracted from 10 μL of each lysate with 50 μL acetonitrile containing the internal standards (reserpine at 100 ng/mL). After a centrifugation step at 50000 g for 10 min, an aliquot of 50 μL of the supernatant was transferred to an autosampler vial and analyzed. The compounds were quantified by LC-MS/MS in the selected reaction monitoring mode using HESI ionization in positive ion mode, using a TSQ Quantum Access Mass spectrometer (Thermo Fisher Scientific, San Jose, CA, USA). The compound concentration versus time data was determined.

In Vivo Analysis of *P. berghei* Liver Parasite Growth and Transition to Blood Infection

Female BALB/c mice were infected with *Pb*mCherryLuci sporozoites by either intravenous injection or infectious mosquito bite challenge. Mice (infected by either route) received either no treatment or were treated orally with WM382 (prepared as previously described) at doses and h post infection (hpi) as indicated. For i.v. injections, freshly isolated salivary gland sporozoites were filtered through glass wool to remove debris before 40,000 sporozoites were resuspended in 200 μL Schneider's Insect Media immediately prior to injection. For infection by mosquito bite, the percent of mosquitoes that contained oocysts was used to place 5 infected mosquitoes in individual feeding cups (for example, if 83% of mosquitoes in the batch had oocysts then 6 mosquitoes were placed in each cup). Mice were anesthetised using ketamine/xylazine and placed on individual feeding cups to begin the infection. Mosquitoes were allowed to feed on the mice for 15 min and mice were rotated between feeding cups every min to promote probing and ensure that all mice were equally exposed to infectious mosquito bites. From day 3 – 30 post infection mice were monitored daily for the presence of parasites in Giemsa stained thin blood smears and considered to be protected from blood infection if they remained negative through this period.

In addition to monitoring thin blood smears, luciferase activity was used to measure liver infection (52 hpi), liver egress (55 hpi) and transition to initial blood infection (65 hpi) in mice infected intravenously. Briefly, mice were injected i.p. with 200 μL XenoLight D-Luciferin (PerkinElmer) and returned to their cage for 5 min prior to anesthesia by isofluorane inhalation for a further 5 min. Between 10 – 15 min post injection of D-luciferin, mice were maintained under isofluorane anesthesia and bioluminescent signal collected using an IVIS Lumina S5 system (PerkinElmer) with a medium binning factor and 3 min integration time. Bioluminescence emanating from a region of interest (ROI) covering the liver region (52 and 55 hpi) or whole animal (65 hpi) was quantified using Living Image software version 4.7.2 (PerkinElmer) and expressed as the total flux in photons/second (p/s). Background flux was measured using an identically sized ROI placed over the lower pelvic region below the liver (52 and 55 hpi) or uninfected mice (65 hpi) and is expressed as the limit of detection (LOD). The parasitemia of the first round of erythrocytic infection was measured at 65 hpi using a LSRFortessa X20 (Becton Dickinson) flow cytometer. Briefly, a drop of whole blood was collected from the tail in 200 μL human tonicity (HT) PBS and immediately analyzed. To identify infected blood cells, mCherry signal intensity was plotted against GFP signal intensity as a decoy reporter. The number of $mCherry^+$ / GFP^- events are reported per 1 × 10^6 whole blood cells with blood from uninfected mice used as a negative control.

In Vitro Analysis of *P. berghei* Liver Parasite Detachment and Merosome Transfer to Mice

To infect cells, freshly isolated *Pb*mCherryLuci salivary gland sporozoites were resuspended in complete medium and 10^4 sporozoites added to each well (MOI 0.34) before spinoculation at 500 x g for 3 min. Sporozoites were allowed to infect cells for 3 h before washing with complete medium. From 24 hpi complete medium containing WM382 (1 nM or 100 nM), or DMSO vehicle (0.1% v/v final concentration) was added to infected wells in technical triplicate. The compound containing medium was changed the next morning before 48 hpi to prevent removal of detached cells and merosomes in the supernatant at later time points (Sturm et al., 2006). To measure inhibition of parasitised cell detachment in the treated cultures, each well was imaged for mCherry at 20x magnification using an Opera Phenix High Content Screening System (PerkinElmer) at 48 hpi (live cell microscopy) and again at 65 hpi following fixation with 4% formalin in PBS, and the number of $mCherry^+$ parasites counted using the Columbus Image Data Storage and Analysis System (PerkinElmer). Cell detachment was calculated as the percent of parasites lost from the monolayer between 48 – 65 hpi. The culture supernatant containing merosomes and detached parasitised cells from each well was collected at 65 hpi, washed once in complete media by centrifugation at 500 x g for 3 min, then i.v. injected into individual male Swiss mice. Daily Giemsa stained thin blood smears were monitored for patent blood infection as described previously.

Immunofluorescent Assay for *P. berghei* Liver Parasites

Following fixation at 65 hpi, infected hepatocyte monolayers were blocked and permeabilized in PBS containing 1% BSA and 0.1% Triton X-100 (blocking buffer) for 1 h before incubation with polyclonal Goat-anti-*Pb*UIS4 (1:500) and Rabbit-anti-*Py*MSP1_{19} antiserum (1:200) for 1 h. After 3 washes in PBS to remove primary antibodies, wells were incubated sequentially with secondary antibodies recognizing Goat IgG (Alexa Fluor 488; 1:500) and then Rabbit IgG (Alexa Fluor 633; 1:500) for 1 h, separated by 3 washes with PBS. All antibody incubations were performed in blocking buffer at room temperature protected from light, and 2 μg/mL DAPI was added to the last incubation with secondary antibody to stain DNA. The center of each well was then imaged across a 12 μm z stack at 63x magnification using an Opera Phenix (PerkinElmer) for the above fluorochromes as well as mCherry to reveal parasite cytoplasm. Data was transferred to Columbus (PerkinElmer) and at least 70 parasites per condition were then manually scored based on a previously described staging system (Graewe et al., 2011). Briefly, parasites without parasite plasma membrane invagination revealed by MSP1 staining were classified as 'schizont' stage while those with clear membrane invaginations surrounding multiple nuclei were scored as 'cytomere' stage. More extensive membrane invaginations surrounding individual nuclei with well segmented parasite cytoplasm and intact UIS4 staining were scored as 'merozoite' stage. 'Egressed' parasites were classified as either well segmented merozoite stages with evidence for PVM breakdown, or largely intact PVM vacuoles in which significant loss of parasite material was evident (Figure S6).

Peptide Cleavage Assays

For all cleavage assays, 10-point dilution series of the compounds (Compound EC_{50} determination on Figures 2B and 2C) were prepared in 384 well black low volume assay plates (Corning #4514) using an Echo555 (Labcyte). Appropriate volumes of 10 mM compound stocks were transferred into the assay plates such that the starting concentration was 90 μM (PMV), 11.25 μM (Renin, Cathepsin D and BACE assays) or 100 nM (PMX assay), all with a 1:3 fold dilution series. All wells were backfilled to 200 nL DMSO such that it remains constant across the assay plates (1% final). IC_{50} values were calculated by Dotmatics 5.3 and Spotfire 7.11.1 software using a nonlinear regression four-parameter fit analysis. The equation used is sigmoidal dose response (variable slope), $Y = bottom + (top - bottom)/(1+10((logEC_{50} - X) \times \text{Hill Slope}))$. Compound Ki values were calculated from the IC_{50}s using the Cheng-Prusoff equation, $K_i = IC_{50}/(1 + [S]/K_m)$.

All compound potency assays were conducted in 20 μl total volume. For each assay, 10 μl of recombinant enzymes in respective assay buffers (Table) were dispensed into compound containing assay plates using a Multidrop Combi dispenser and allowed to incubate for 15 min. The reactions were started with a further 10 μl addition of FRET peptide substrates and reactions incubated at 37 °C for various times (Table).Samples were excited at 340 nm and fluorescence emission measured at 492 nm using an Envision fluorescence plate reader (Perkin-Elmer). The 0% inhibition control contained DMSO (1% final) and the 100% inhibition control was minus enzyme.

Table. Peptide Cleavage Assays Parameters

	PMX	PMV Assay	Renin Assay	Cathepsin D Assay	BACE1 Assay
Enzyme quantity	0.05 nM enzyme (in-house)	2.5 nM enzyme (in-house)	Renin: 3 nM enzyme (Proteos #R-001)	0.25nM enzyme (Athens Research #16-12-030104)	6nM enzyme (Sigma #S4195)
Buffer	25mM Sodium Acetate, 0.005% Tween-20, pH 5.5	25mM MES, 25 mM Tris-HCl, 0.01% Tween-20, pH 6.4	50mM Tris, 100mM NaCl, 0.1% Brij-35, pH 8.0	100mM Sodium Acetate 0.02% Brij-35, pH 5.0	20mM Sodium Acetate, 0.05% Brij-35, pH 4.5
Substrate peptide	DABCYL-HSFIQEGKEE-EDANS	DABCYL-RNKRTLAQKQE-EDANS	DABCYL-(γ-Abu)-IHPFHLVIHTE-EDANS	Ac-E[E(EDANS)] KPILFFRLGK (DABCYL) E -NH2	RE(EDANS) EVNLDAEFK (DABCYL)R
Peptide final concentration	1.6 μM	12 μM	20 μM	0.5 μM	5 μM
Incubation time at 37 °C	4 h	90 min	45 min	30 min	240 min

PMX substrate's cleavage efficiency assay (Figure 2D) was performed against a panel of peptide substrates (Figure S4C) in 20 μl total volume with 10 nM PfPMX and 10 μM substrate and the same buffer as described above. Fluorescence signal was measured after 4 h on the Envision plate reader as described above.

K_m determination (Figure 2E) for Rh2N and SUB1 substrate was conducted in a 384 well black low volume assay plate (Corning # 4514) in 20 μl total volume with 0.3 nM PfPMX and peptide substrate concentration series starting at 12.5 μM in 1:2 fold dilution for 8 points. The enzyme kinetic assay was conducted at 37 °C for 1 h (Rh2N) and 6 h (SUB1) on the EnVision plate reader as described above. GraphPad Prism software was used to plot the kinetic data, determine the initial velocity (RFU/min) and calculate the substrate K_m based on Michaelis-Menten enzyme kinetics.

Rationale for Identification of Potential PMX and PMIX Protein Substrates

To identify the PMX cleavage site of Rh2a/b N-terminal & Rh2b C-terminal proteins they were affinity purified and analyzed by mass spectrometry (Figure S7) and N-terminal sequencing. This determined that Rh2a/b proteins were cleaved at 'SFIQ and 'SLVQ' motifs respectively (Figure S4C). These cleavage sites were similar to those that have now been published for AMA1 and SUB1 (Pino et al., 2017). Similar affinity purification and mass spectrometry of processed Rh1, Ripr and Rh5 proteins, showed that these were cleaved at a four amino acid motif with similarity to those identified in the Rh2 proteins (Figure S4C). Since Rh2a/b proteins were assumed to be stored in the microneme prior to being transported to the merozoite apical tip, we reasoned that other micronemal proteins may also be PMX cleaved. This included Rh1, Rh4, Rh5, Ripr, EBA140, EBA175, EBA181 and AMA1, which we showed were cleaved by PMX using the Processing Inhibition Assay (see below). Analysis of the protein sequences, showed they all had one or more potential PMX cleavage sites. Hence, we obtained or produced HA-tagged parasites in cases where we had no specific antibodies to the proteins or used available specific antibodies. These parasites and antibody reagents were used to prove that these proteins were processed by PMX or PMIX using the chemical tools WM4 and WM382. In addition we showed that the identified protease motifs were cleaved by PMX as Synthetic fluorogenic peptides corresponding to *P. falciparum* sequences.

Processing Inhibition Assays

Processing inhibition assays were performed as previously described (Boyle et al., 2010). Synchronized late trophozoite/early schizont cultures to which protease inhibitors had already been added, were passed over LD magnetic columns (Miltenyi Biotech) to remove uninfected erythrocytes. The cysteine protease inhibitor E64 (Sigma) was used at 10 μM. The inhibitors WM4 at 40 nM and WM382 at 2.5 nM final concentrations, were used at approximately 5xEC_{50} concentrations. A control dish without any protease inhibitor was also included. Parasites were eluted from columns with complete RPMI 1640 culture medium to which the appropriate inhibitor at the same concentration had been added. Eluted parasites were adjusted to 5x10^6 schizonts/mL and 150 ul added per well of a 96-well flat-bottomed culture dish. The assay dishes were further cultured for 16 h and a representative well from each condition smeared for Giemsa staining, to ensure either that rupture had occurred normally (control well) or that rupture had been blocked (E64, WM4, WM382 conditions). Parasites from each condition were spun at 10000 g/10 min so that merozoite and supernatant fractions could be separately collected. Proteins from both fractions were extracted with Reducing sample buffer and separated on 4%–12% or 3%–8% acrylamide gels (NuPAGE, Invitrogen).

P. falciparum Schizont Egress Inhibition Assay

Synchronized trophozoites (3D7) were treated with compounds WM4 or WM382 at various concentrations, or vehicle control (DMSO) and allowed to develop until late schizonts and early ring stage parasites were observed in the control plates. Parasites purified by magnet separation (MACS; Miltenyi Biotec) were eluted into complete RPMI containing 1 nM Compound 1 (C1) to prevent further egress and synchronize egress to within a 5 min window (Pino et al., 2017). When schizont preparations were fully mature, the C1 was washed out with warm complete RPMI-HEPES medium and schizonts transferred to a microscopy chamber for live video time lapse (1 frame per 0.5 s) imaging under DIC conditions using a Zeiss Live Cell Axio observer microscope with x63 oil immersion objective and Zen Blue software used for image capture. Videos (Videos S1 and S2) were processed using Fiji (https://www.nature.com/articles/nmeth.2019). Total parasite numbers and egress events were calculated over 5 min window and expressed as average ± SD of 3 replicate experiments. Because the purified late-stage parasites cannot be fully synchronized to egress within the observation time window, only a proportion of the schizonts will egress during the period of observation. Because all treatment groups originated from the same culture (ie those treated with compound and DMSO vehicle control) the developmental stage is consistent between treatments and the difference in egress quantitation can be attributable to the drug treatment.

QUANTIFICATION AND STATISTICAL ANALYSIS

All graphs of experimental data and statistical analyses were generated with GraphPad Prism 8.2, Dotmatics 5.3 and Spotfire 7.11.1 softwares. Statistical details of experiments are indicated in the figure legends and Method Details.

DATA AND CODE AVAILABILITY

The accession number for the sequencing data reported in this paper is ENA: PRJEB36069. This study did not generate any novel code.

Cell Host & Microbe, Volume 27

Supplemental Information

Dual Plasmepsin-Targeting Antimalarial Agents Disrupt Multiple Stages of the Malaria Parasite Life Cycle

Paola Favuzza, Manuel de Lera Ruiz, Jennifer K. Thompson, Tony Triglia, Anna Ngo, Ryan W.J. Steel, Marissa Vavrek, Janni Christensen, Julie Healer, Christopher Boyce, Zhuyan Guo, Mengwei Hu, Tanweer Khan, Nicholas Murgolo, Lianyun Zhao, Jocelyn Sietsma Penington, Kitsanapong Reaksudsan, Kate Jarman, Melanie H. Dietrich, Lachlan Richardson, Kai-Yuan Guo, Sash Lopaticki, Wai-Hong Tham, Matthias Rottmann, Tony Papenfuss, Jonathan A. Robbins, Justin A. Boddey, Brad E. Sleebs, Hélène Jousset Sabroux, John A. McCauley, David B. Olsen, and Alan F. Cowman

Supplemental Information

A

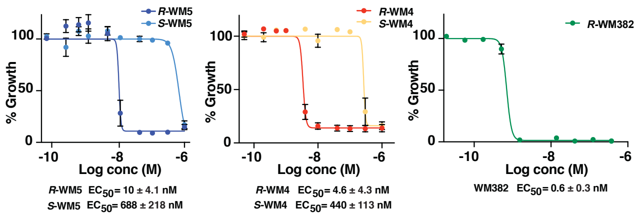

B

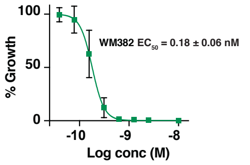

C

Parasite line	Start of amplicon	End of amplicon	Size of amplicon (kb)	Sequence end, start	Copy Number	EC_{50} (nM)	
						WM5	WM4
3D7	-	-	-	-	1	11.1±3.7*	14.2±6.5#
3D7-WM5.1	08: 409,250	08: 429,220	20	45xT, 31xT	8-10	106.8±2.2*	115.0±18.8#
3D7-WM5.2	08: 393,290	08: 426,970	34	51xT, 30xA	7-8	116.9±6.4*	141.7±5.5#
3D7-WM5.3	08: 409,250	08: 429,210	20	45xT, 31xT	8-10	117.9± 4.7*	135.3±3.8#
3D7-WM4.1	08: 398,500	08: 429,200	31	27xT, 31xT	6	51.82±19.7#	65.9±34.8#
3D7-WM4.2	08: 415,500	08: 429,200	14	20xT, 31xT	8	143.77± 46.6#	139.5± 52.7#

* ± SEM # ± SD

D

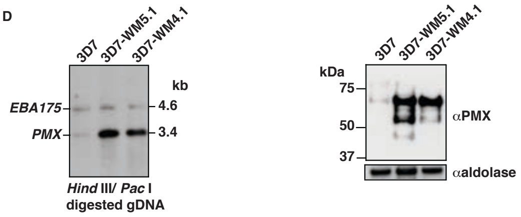

Supplementary Figure 1. Related to Figure 1. Growth inhibition of WM4, WM5 and WM382 enantiomers against *P. falciparum* and analysis of WM4 and WM5-resistant parasite lines. A. The 3D7 parasite line was used to derive growth inhibition plots for *R* and *S* enantiomers of WM5 (left), *R* and *S* enantiomers of WM4 (middle) and the *R* enantiomer of WM382 (right). Each experiment was performed in triplicate; mean with SEM are shown. **B.** *P. knowlesi* blood stage parasites were used to derive a growth inhibition plot for WM382. Dots represent the average of 3 repeats carried out in triplicate; error bars represent SD. **C.** The breakpoints in the genome of *P. falciparum* parasites selected for resistance to WM5 and WM4. The genomes of the WM5-resistant parasites 3D7-WM5.1, 3D7-WM5.2, 3D7-WM5.3 and WM4-resistant parasites 3D7-WM4.1 and 3D7-WM5.2 were sequenced and amplification events defined. The specific breakpoints for each were determined to define the start and end of the amplicon as well as the size of each amplification unit. Also determined were the EC_{50} of each parasite line for both WM4 and WM5. **D.** Left panel: southern blot of genomic DNA from 3D7-WM5.1 and 3D7-WM4.1 to confirm amplification of the *PMX* gene, compared to 3D7 wild type parasites. gDNA was digested with *Hind* III/*Pac* I and probed with both *PMX* and EBA175 genes. The expected 3.4 kb band for *PMX* and the expected 4.6 kb band for the EBA175 gene are shown. Right panel: immuno-blot of 3D7 WT, 3D7-WM5.1 and 3D7-WM4.1 schizont parasites with anti-PMX antibodies to show that this enzyme was overexpressed in the WM5- and WM4-resistant parasite lines, compared to 3D7 wild type parasites. An aldolase panel is shown at the bottom as a loading control. Aldolase was detected with anti-aldolase antibodies.

A

	EC_{50} (nM)				
Parasite	Chloroquine	Atovaquone	Mefloquine	WM4	WM382
3D7	14.5 ± 0.7	1.95 ± 0.1	10.4 ± 2.2	7 ± 0.5	0.48 ± 0.3
3D7-WM4.1	10.65 ± 3.3	0.97 ± 0.03	5.9 ± 1.13	58 ± 1.4	0.68 ± 0.1
CAM3R539T	114.5 ± 21.9	2.05 ± 0.06	9.15 ± 2.6	13 ± 1.4	0.25 ± 0.04
CAM3 REV	102.5 ± 38.8	1.05 ± 0.07	3.8 ± 0.0	12 ± 1.4	0.22 ± 0.01
Dd2	99.5 ± 14.8	1.85 ± 0.07	11.5 ± 2.1	8.55 ± 0.3	0.26 ± 0.01
Dd2-ATV	120 ± 28.2	28.5 ± 3.5	3.2 ± 0.1	5.85 ± 0.3	0.23 ± 0.02
K1	160 ± 14.1	2.4 ± 0.4	4.75 ± 2.7	13.5 ± 2.1	0.65 ± 0.4
K1Mef[2]	120 ± 14.1	5.45 ± 4.1	19.5 ± 13.4	11 ± 0	0.36 ± 0.09
W2Mef[3]	99.5 ± 28.9	2.95 ± 0.35	25 ± 2.8	9.4 ± 2.2	0.25 ± 0
PNG1776	69 ± 21.2	1.1 ± 0.14	1.95 ± 0.2	5.6 ± 1.4	0.21 ± 0

B

		EC_{50} (nM)	
	RSA survival	WM5	WM4
3D7	7.77%	12	13
3D7-WM4.1	9.96%	103	102
3D7-WM5.1	9.72%	36	69
Cam3 R539T	32.81%	90	29
Cam3 Rev	11.43%	73	33

C

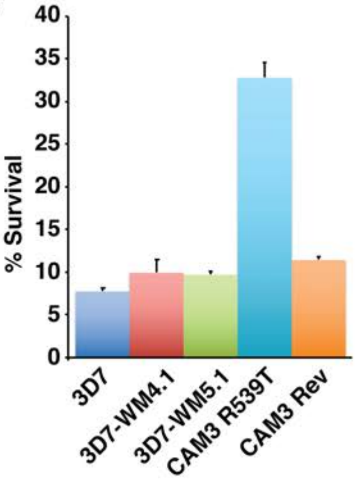

Supplementary Figure 2. Related to Figure 1. WM4 and WM382 do not show cross-resistance with *P. falciparum* lines resistant to chloroquine, atovaquone, mefloquine and artemisinin. A. EC_{50} determination for different *P. falciparum* lines shown (Parasite) to chloroquine, atovaquone, mefloquine, WM4 and WM382. Experiments have been done twice independently with three replicas per experiment. Shown is mean and standard deviation. **B** and **C.** Ring-stage survival assay to determine resistance to artemisinin. Shown are experiments done three independent times.

A

Parasite lines	Day 0 IC_{50} (nM)	Day 14 IC_{50} (nM)	Day 28 IC_{50} (nM)
3D7	10.96	13.95	11.65
3D7-WM5.1	202.8	228.5	177.5
3D7 + 3D7-WM5.1	87.9	120.6	17.33
3D7	5.9	5.2	5.4
3D7-WM4.1	78.8	89.9	79.4
3D7 + 3D7-WM4.1	34.1	42.1	7.3

B

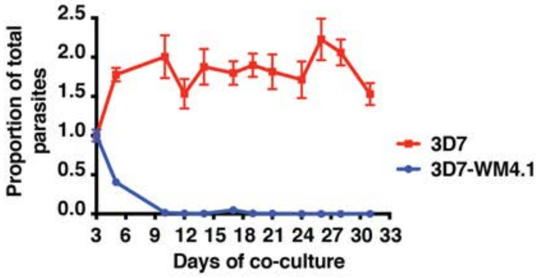

C

Experiment 1 (90 days)

Compound	Selection Pressure (nM)	Initial Inoculum	Outcome
Atovaquone	10	1x10^9<br>1x10^8<br>1x10^7<br>1x10^6<br>1x10^5	-<br>-<br>1 of 1 (day 56)<br>-<br>-
WM382	1.5	1x10^9<br>1x10^8<br>1x10^7<br>1x10^6<br>1x10^5	-<br>-<br>-<br>-<br>-

Experiment 2 (60 days)

Compound	Selection Pressure (nM)	Initial Inoculum	Outcome
Atovaquone	10	1x10^8<br>1x10^7<br>1x10^6	1 of 3 (day 41)<br>-<br>-
WM4	80	1x10^8<br>1x10^7<br>1x10^6	-<br>-<br>-
WM382	1.5	1x10^8<br>1x10^7<br>1x10^6	-<br>-<br>-

Supplementary Figure 3. Related to Figure 1. Time to resistance and fitness studies. A. 3D7-WM5.1 and 3D7-WM4.1 parasites are less fit then the 3D7 parental parasite line. 3D7 and 3D7-WM5.1 or 3D7-WM4.1 were mixed together in a 1:1 ratio and EC_{50} determined at day 0, 14 and 28 as a measure of the proportion of each parasite in the mixture over time. **B.** 3D7 and 3D7-WM4.1 were mixed together in a 1:1 ratio and the level of each parasite in the population monitored over 33 days by real time quantitative polymerase chain reaction. The proportion of each population was determined relative to the control RNA actin. **C.** Time to resistance studies comparing the selection of atovaquone resistance to that of WM4 and WM382. The initial inoculum of *P. falciparum* parasites was varied from 1x10^5 to 1x10^9 and these were selected on a concentration equivalent to the EC_{90} of WM4, WM382 and atovaquone and recrudescence of parasitemia followed over 96 and 60 days for each experiment respectively. Shown are two independent experiments.

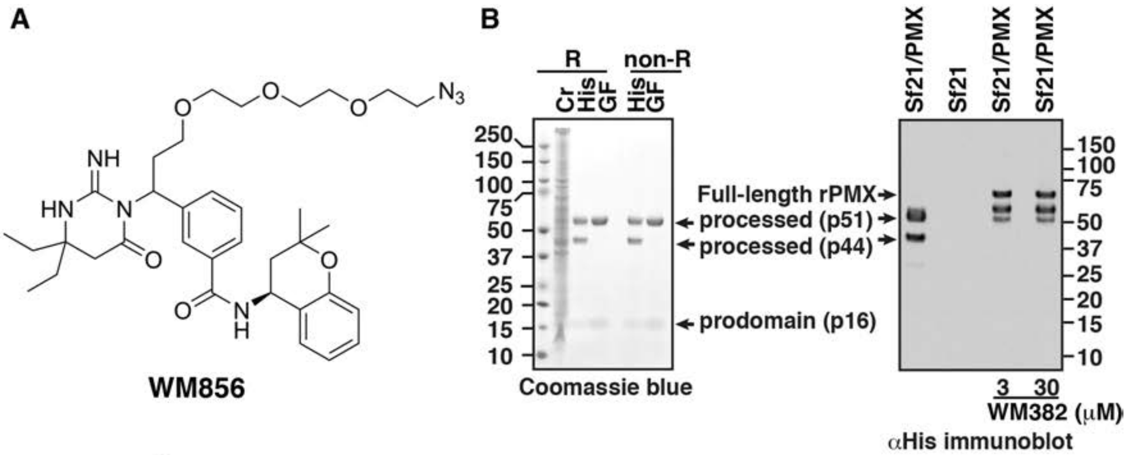

C

PROTEASE	PROTEIN	SEQUENCE									
PMX	SUB1	D	Y	K	S	M	L	E	V	E	N
PMX	AMA1	N	L	F	S	S	I	E	I	V	E
PMX	Rh2N	S	F	H	S	F	I	Q	E	G	K
PMX	Rh2C	H	T	A	S	L	V	Q	I	T	Q
PMX	Rh1	K	P	F	F	F	I	Q	L	N	T
PMX	Rh4	I	L	N	S	F	V	Q	I	N	S
PMX	Rh5	K	N	V	N	F	L	Q	Y	H	F
PMIX	Ripr	N	N	I	S	M	L	E	I	Q	N
PMX	EBA181	N	R	N	S	F	V	Q	R	S	Y
PMX	EBA175	R	I	L	S	F	L	D	S	R	I
PMX	EBA140	K	F	N	S	L	S	E	N	E	D
PMX	PMX	K	N	F	I	A	L	E	N	K	N
PMX	PMX	S	D	N	S	S	I	E	K	N	F
PMX	SUB2	S	K	R	S	N	I	E	K	E	I
PMIX	PMIX	D	N	T	N	T	S	Q	E	N	F
PMIX	RON3	K	E	I	S	F	L	E	R	R	E
PMIX	RAP1	S	S	E	S	F	L	E	N	K	S
PMIX	ASP	K	F	L	S	L	L	Q	L	N	L

Supplementary Figure 4. Related to Figure 2. Expression and purification of active recombinant *P. falciparum* PMX enzyme and analysis of cleavage site consensus sequence. A. Chemical structure of WM856. The compound was used for cross-linking to sepharose beads and affinity purification of PMX. **B.** Expression and purification of *P. falciparum* PMX from insect cells. PMX was purified from insect cell crude extract (Cr) by Histidine affinity purification (His) and size exclusion chromatography (GF). Three bands were identified by Coomassie stained gel electrophoresis. Mass spectrometry of each band identified them as the full-length enzyme with prodomain and catalytic domain (pro-cat) of approximately 60 kDa, the catalytic domain (cat) of 45 kDa and the prodomain (prodom) of 16 kDa. R, reduced; non-R, nonreduced. **C.** PMX and PMIX cleavage sites for SUB1 and RAP (Pino *et al.*, 2017) and potential cleavage sites of other proteins from *P. falciparum*. The conserved amino acids that make up the consensus sequence are shown in red.

A

Substrates	Parent	Processed by PMX	
SUB1	DABCYL-G-SMLEVENDAE-GE-EDANS $[M+H]^+$ 1878 (Expected 1878)	DABCYL-G-SM $[M+H]^+$ 545 (Expected 545)	LEVENDAE-GE-EDANS $[M+H]^+$ 1352 (Expected 1352)
RAP1	DABCYL-G-FSSESFLENK-E-EDANS $[M+H]^+$ 1873 (Expected 1873)	DABCYL-G-FSSESF $[M+H]^+$ 1011 (Expected 1011)	LENK-E-EDANS $[M+H]^+$ 879 (Expected 879)
Rh2N	DABCYL-HSFIQEGKE-E-EDANS $[M+H]^+$ 1703 (Expected 1703)	DABCYL-HSF $[M+H]^+$ 641 (Expected 641)	IQEGKE-E-EDANS $[M+H]^+$ 1080 (Expected 1080)
RON3	DABCYL-KEISFLERR-E-EDANS $[M+2H]^{2+}$ 903 (Expected 903)	DABCYL-KEISF $[M+H]^+$ 874 (Expected 874)	LERR-E-EDANS $[M+H]^+$ 950 (Expected 950)
EBA175	DABCYL-RILSFLDSRI-E-EDANS $[M+2H]^{2+}$ 924 (Expected 924)	DABCYL-RILSF $[M+H]^+$ 886 (Expected 886)	LDSRI-E-EDANS $[M+H]^+$ 980 (Expected 980)
EBA181	DABCYL-NRNSFVQRSY-E-EDANS $[M+2H]^{2+}$ 950 (Expected 950)	DABCYL-NRNSF $[M+H]^+$ 888 (Expected 888)	VQRSY-E-EDANS $[M+2H]^{2+}$ 514 (Expected 514)

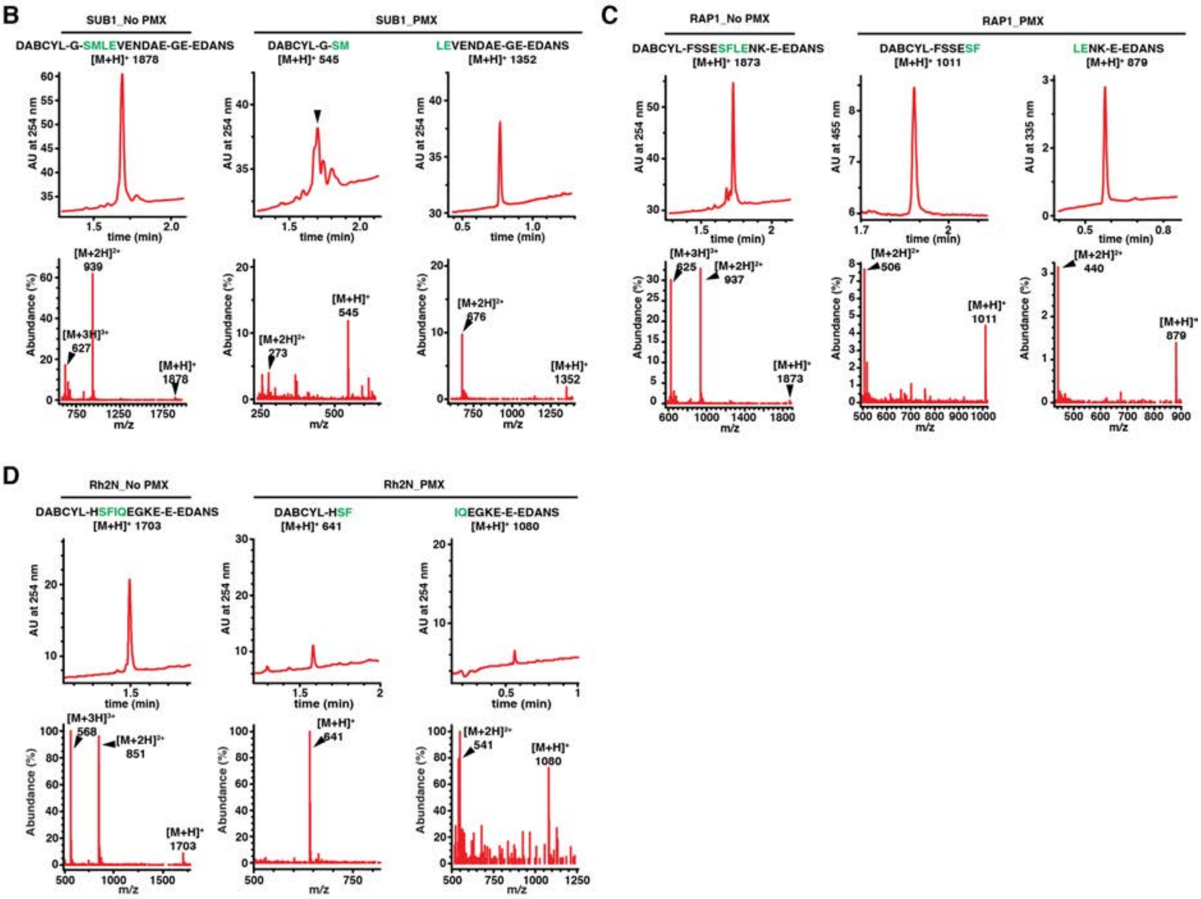

Supplementary Figure 5. Related to Figure 2. Mass spectrometry identification of *P. falciparum* peptide species before and after recombinant PMX cleavage. A. LC-ES/MS analysis of peptides and cleavage products after incubation with recombinant PMX (rPMX). Shown are the experimental and expected (in brackets) mass of each molecular species processed by rPMX. **B.** SUB1peptide without and with rPMX and analysed by LC-ES/MS to identify the molecular species present. **C.** RAP1 peptide incubated without and with rPMX and analysed by LC-ES/MS to identify the molecular species present. **D.** Rh2N peptide incubated without and with rPMX and analysed by LC-ES/MS to identify the molecular species present. For all substrates, SUB1, RAP1 and Rh2N, RON3, EBA175 and EBA181 fluorogenic peptides (50 µM) were incubated with and without rPMX (50 nM) and the products detected LC-ES/MS, indicating cleavage occurs on the C-terminal side of a hydrophobic amino acid (usually Phe).

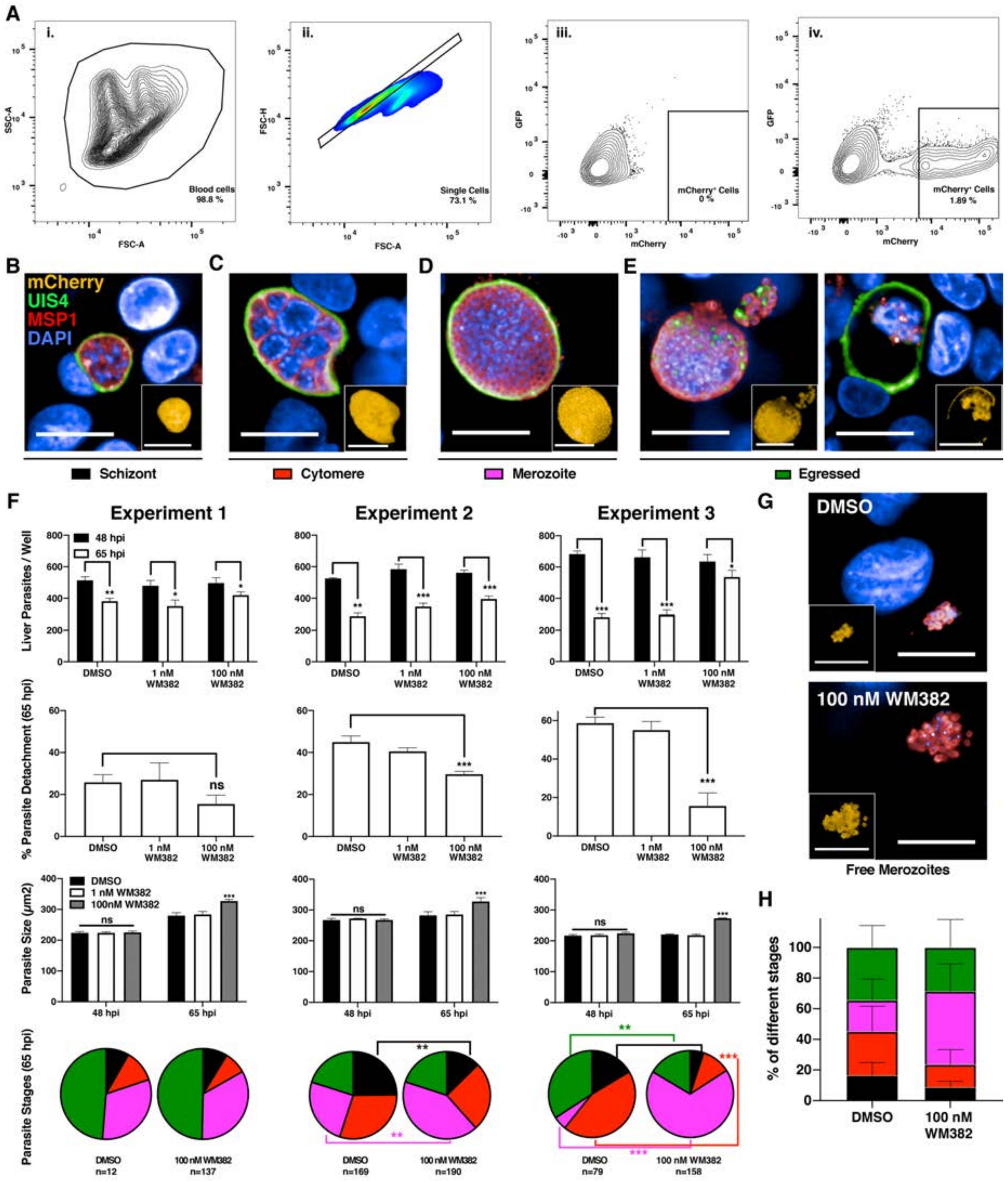

Supplementary Figure 6. Related to Figure 5. Flow cytometry analysis of *Pb*mCherryLuci infected whole blood and *in vitro* analysis of WM382 activity against *P. berghei* liver parasites (data supporting Fig. 5). A. Using a drop of tail blood, at least 1 x 10^6 events in the whole blood cells gate were collected (i) from which single cells were gated for analysis (ii). The mCherry$^+$ gate was set using an uninfected sample (iii) with a positive control shown (iv). **B-H.** Classification of *Pb*mCherryLuci liver parasites from infected HepG2 cultures was performed at 65 hpi and results from 3 independent experiments are shown. **B.** Example of a schizont stage without plasma membrane invaginations (MSP1, red) and intact UIS4$^+$ parasitophorous vacuole membrane (PVM, green) that was quantitated below (black) in the pie charts (F, bottom panels); inset shows mCherry fluorescence (orange, cytoplasmic).

C. Example of a cytomere stage with intact PVM (UIS4, green) and clear plasma membrane invaginations (MSP1, red) surrounding multiple nuclei (DAPI, blue) that was quantitated below (blueblue) in the pie charts (F, bottom panels). **D.** Example of a merozoite stage with extensive membrane invaginations (MSP1, red) surrounding single nuclei (DAPI, blue) with well-defined cytoplasm (mCherry, orange) and intact PVM (UIS4, green) that was quantitated below (magenta) in the pie charts (F, bottom panels) **E.** Example of egressed stages with evidence for PVM degradation (UIS4, left) or significant loss of parasite material within UIS4^{+} vacuoles (right) that was quantitated below (yellowyellow) in the pie charts (F, bottom panels). **F.** Three independent experiments examining the effects of DMSO (control) and WM382 (1 nM and 100 nM) treatment from 24 hpi on *Pb*mCherryLuci liver parasite growth and egress using HepG2 cells *in vitro.* **1st row:** Treatment with 1 nM or 100 nM WM382 did not reduce the number of liver parasites at 48 hpi, indicating no parasite killing by WM382. The number of parasites per well decreased from 48 hpi to 65 hpi in all conditions, indicating that WM382 treated parasites are able to detach from the monolayer to be released into the supernatant. **2nd row:** Quantitation of parasite detachment and loss from the monolayer between 48 and 65 hpi suggests a decrease in detachment when treated with 100 nM WM382. **3rd row:** There was no difference in parasite size at 48 hpi consistent with there being no cidal or static effects of WM382 on liver parasite growth. There was however an increased size of parasites treated with 100 nM WM382 at 65 hpi consistent with an inhibitory effect on egress and detachment of mature *P. berghei*-infected hepatocytes. **4th row:** Quantification of schizont, cytomere, mature merozoite and egressed forms shows that developmentdevelopment to merozoite stages is normal in 100 nM WM382 treated cultures, and that at least some parasites treated with 100 nM WM382 successfully egress. Consistent with a decrease in cell detachment (second row) and increase in parasite size (third row), 100 nM WM382 had an increased proportion of merozoite stage parasites indicating an inhibition of parasite egress that precedes cell detachment. **G.** Free merozoites (DAPI, blue; MSP1, red; inset, mCherry cytoplasm) were seen in both control and 100 nM WM382 treated wells, demonstrating a level of successful exoerythrocytic merozoite egress was taking place in the presence of 100 nM WM382. **H.** Averaged liver parasite staging data from 3 independent experiments with mean ± SD shown. Data in (F) represent the mean ± SD from technical replicates with analysis by t-test (top row), One-way ANOVA with Dunnett's multiple comparison test (middle rows) and Fisher's exact test (bottom row). $*p<0.05$, $**p<0.01$, $***p<0.001$; scale bar 20 µm. This analysis shows that there is an incomplete inhibitory effect of WM382 on egress of *P. berghei* exoerythrocytic merozoites from hepatocytes.

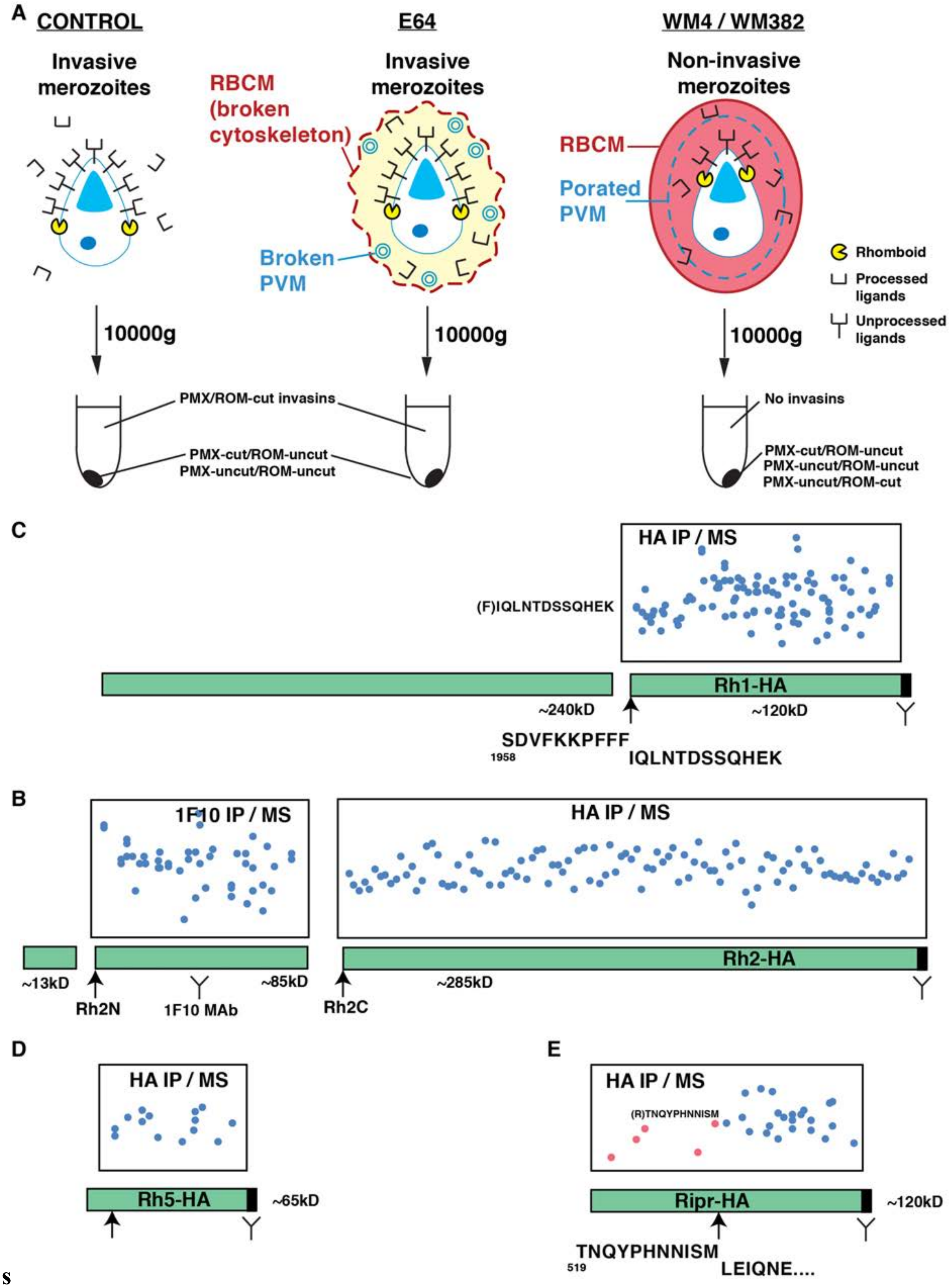

s

Supplementary Figure 7. Related to Figure 6. Model of drug treated schizonts and mass spectrometry analysis of affinity purified *P. falciparum* parasites. A. Untreated (control) or drug-treated schizont parasites were purified using magnetic columns and then placed in 96 well trays with medium and incubated for a further 16 hr. Merozoites egress from untreated control schizonts (left of panel) with release of PMX and PMIX processed apical organelle contents as well as shedding of proteins from the surface due to the action of SUB2 and

rhomboid (ROM) protease. This releases the ligands into the supernatant and proteins remaining in the merozoite are centrifuged in the pellet. E64-treated schizonts (middle of panel) have a broken parasitophorous vacuole (PVM) but an intact – but leaky- red blood cell membrane (RBCM), as a result of blocking of SERA6 activity, consequently, ligands are able to move through to the supernatant including ROM and PMX- cleaved proteins. Proteins uncleaved by PMX and ROM are not released from the merozoite and are centrifuged to the pellet. WM4 or WM382 schizonts have an intact RBCM, since SUB1 (which is required to activate multiple proteins including not only SERA6, but also possibly pore-forming proteins and phospholipases implicated in egress of other parasite stages) (Thomas *et al.*, 2018) is not proteolytically active when it has not been cleaved by PMX, so that most proteins are retained and centrifuged to the pellet. PMX or PMIX inhibit processing of some proteins and these are detected in the pellet in immunoblots. **B.** Rh2a/b processed proteins were affinity purified with monoclonal antibody 1F10 and Rh2b with anti-HA antibodies and specific protein bands separated by SDS/PAGE and trypsin digested and analysed by mass spectrometry. The dots refer to individual peptides identified. The arrows refer to the site of the PMX consensus cleavage sequence for both Rh2N and Rh2C peptides that are cleaved by recombinant PMX (Fig. 2 and Fig. S4). **C.** The Rh1120 kDa protein band was affinity purified with anti-HA antibodies and separated by SDS/PAGE, trypsin and chymotrypsin digested and analysed by mass spectrometry. The dots refer to individual peptides identified. The arrow refers to the site of the cleavage identified from both a semi-tryptic and a semi-chymotryptic peptide by mass spectrometry and corresponds to the PMX consensus cleavage sequence for Rh1. The amino acid position in the Rh1 protein is indicated below the peptide. **D.** Rh5 was affinity purified with anti-HA antibodies and the 45 kDa processed protein separated by SDS/PAGE, trypsin digested and analysed by mass spectrometry. The dots refer to individual peptides identified. The arrow refers to the approximate site of the cleavage identified by mass spectrometry and corresponds to the PMX consensus cleavage sequence for Rh5. **E.** The Ripr 60 kDa processed protein band was affinity purified with anti-HA antibodies and separated by SDS/PAGE, trypsin digested and analysed by mass spectrometry. The dots refer to individual peptides identified. The two processed bands of 60 kDa co-migrate so that there are some peptides from the N-terminus from which a semi-tryptic peptide was identified and shown with an arrow and corresponds to the PMX consensus cleavage sequence for Ripr. The amino acid position in the Rh5 protein is indicated below the peptide.

References

Pino, P., Caldelari, R., Mukherjee, B., Vahokoski, J., Klages, N., Maco, B., *et al*. (2017). A multistage antimalarial targets the plasmepsins IX and X essential for invasion and egress. *Science* **358,** 522-528.

Thomas, J.A., Tan, M.S.Y., Bisson, C., Borg, A., Umrekar, T.R., Hackett, F., *et al*. (2018). A protease cascade regulates release of the human malaria parasite *Plasmodium falciparum* from host red blood cells. *Nat Microbiol* **3,** 447-455.
